# *In-silico Leishmania* Target Selectivity of Antiparasitic Terpenoids

**DOI:** 10.3390/molecules18077761

**Published:** 2013-07-03

**Authors:** Ifedayo Victor Ogungbe, William N. Setzer

**Affiliations:** 1 Department of Chemistry and Biochemistry, Jackson State University, Jackson, MS 39217, USA; 2 Department of Chemistry, University of Alabama in Huntsville, Huntsville, AL 35899, USA; E-Mail: wsetzer@chemistry.uah.edu

**Keywords:** antileishmanial activity, terpenoids, drug targets, docking, *Leishmania*

## Abstract

Neglected Tropical Diseases (NTDs), like leishmaniasis, are major causes of mortality in resource-limited countries. The mortality associated with these diseases is largely due to fragile healthcare systems, lack of access to medicines, and resistance by the parasites to the few available drugs. Many antiparasitic plant-derived isoprenoids have been reported, and many of them have good *in vitro* activity against various forms of *Leishmania* spp. In this work, potential *Leishmania* biochemical targets of antiparasitic isoprenoids were studied *in silico*. Antiparasitic monoterpenoids selectively docked to *L. infantum* nicotinamidase, *L. major* uridine diphosphate-glucose pyrophosphorylase and methionyl t-RNA synthetase. The two protein targets selectively targeted by germacranolide sesquiterpenoids were *L. major* methionyl t-RNA synthetase and dihydroorotate dehydrogenase. Diterpenoids generally favored docking to *L. mexicana* glycerol-3-phosphate dehydrogenase. Limonoids also showed some selectivity for *L. mexicana* glycerol-3-phosphate dehydrogenase and *L. major* dihydroorotate dehydrogenase while withanolides docked more selectively with *L. major* uridine diphosphate-glucose pyrophosphorylase. The selectivity of the different classes of antiparasitic compounds for the protein targets considered in this work can be explored in fragment- and/or structure-based drug design towards the development of leads for new antileishmanial drugs.

## 1. Introduction

Several closely-related protozoan parasites in the genus *Leishmania* are etiological agents for a number of clinical forms of leishmaniasis. These clinical forms of leishmaniasis are characterized as either cutaneous, diffuse cutaneous, disseminated cutaneous, mucocutaneous, visceral or post-kala-azar dermal. Species causing this protozoan disease have been reported in several tropical and Neotropical regions including Africa, the Americas, Eastern Europe, central and western Asia, the Indian subcontinent as well as in Australia. There are over 350 million people at risk of infection in *Leishmania*-endemic regions. There are several treatment options for leishmaniasis, although the effectiveness of the available drugs depends on which clinical form is being treated, and also on the specific geographical location. For a review of the current treatment options please see reference [1]. There remains a need for better chemotherapy for cutaneous, visceral and post-kala-azar dermal leishmaniasis, as well as, *Leishmania*-HIV co-infection.

Current chemotherapy of visceral and cutanoeus leishmaniasis includes miltefosine, a compound that has been demonstrated to inhibit P13K/Akt signaling pathway, and fluconazole, a sterol 14α-demethylase inhibitor. In addition to currently targeted *Leishmania* proteins, several other proteins have also been identified, or suggested as potential drug targets in *Leishmania* [2,3,4,5]. Most of these targets include enzymes that are critical to the metabolism of glucose, sterols, nucleotides and glycosylphosphatidylinositol, as well as enzymes important for the maintenance of trypanothione and polyamine levels. Many of these proteins have been shown to be important to the survival of the parasites. Other targets include cyclin-dependent- and mitogen-activated protein kinases, topoisomerases and cathepsin-like proteases.

Some of the enzymes that are involved in glucose metabolism and are potential drug targets in some species of *Leishmania* include pyruvate kinase (PYK) [6,7], phosphoglucose isomerase (PGI) [8,9], uridine diphosphate-glucose pyrophosphorylase (UGPase) [10], glyceraldehyde-3-phosphate dehydrogenase (GAPDH) [11,12,13], glycerol-3-phosphate dehydrogenase (GPDH) [14,15], triosephosphate isomerase (TIM) [16,17,18], thiol-dependent reductase I (TDR1) [19] and phosphomannomutase (PMM). TDR1 is involved in the regulation of the terminal steps of glycolysis and the enzyme fortuitously catalyzes the activation of antiparasitic antimonial prodrugs while PMM catalyzes the conversion of mannose-6-phosphate to mannose-1-phosphate, which is essential for the biosynthesis of glycoconjugates, and it has been suggested to be a potential drug target [20]. The enzyme that catalyzes the trypanothione-coupled conversion of methylglyoxal, a toxic byproduct of glycolysis, to lactate in *Leishmania*, glyoxalase II (GLO2), has also been suggested as a drug target [21,22] although modeling studies of the enzyme have suggested that inhibition of glyoxalase II will have little effect on toxic glyoxal build up in the cell [23]. In addition to these, the cysteine protease, cathepsin B (CatB) [24,25], as well as oligopeptidase B (OPB) [26,27,28] are also being investigated as potential drug targets. A number of proteins involved in nucleoside and nucleotide metabolism in *Leishmania* have also been investigated as druggable targets. These includes dihydroorotate dehydrogenase (DHODH), an enzyme involved in the *de novo* synthesis of pyrimidine [29,30], deoxyuridine triphosphate nucleotidohydrolase (dUTPase), an enzyme involved in controlling intracellular dUTP levels [31,32,33], nicotinamidase (PnC1), an essential enzyme for the production of NAD^+^ [34], nucleoside hydrolase (NH) [35,36,37], nuceloside diphosphate kinase b (NDKb) [38] as well as phosphodiesterase 1 (PDE1) [39,40,41] and pteridine reductase 1 (PTR1) [42,43,44,45]. Also, proteins involved in co-/post-translational protein processing like *N*-myristoyltransferase (NMT) [46,47,48] and cyclophilins (Cyp) [49,50] have being actively investigated as antileishmanial drug targets as well as charged-tRNA synthesizing enzymes, methionyl-tRNA synthetase [51] and tyrosyl-tRNA synthetase [52].

Numerous phytochemical agents have exhibited either *in vitro* or *in vivo* antileishmanial activity [5,53,54,55,56,57,58,59,60,61]. While the activities of many of these compounds are notable, what is generally unknown are the biochemical targets of these agents. In this study, we have carried out a molecular docking analysis of known antiparasitic plant-derived isoprenoids with established drug targets with known structures available from the Protein Data Bank.

**Figure 1:**
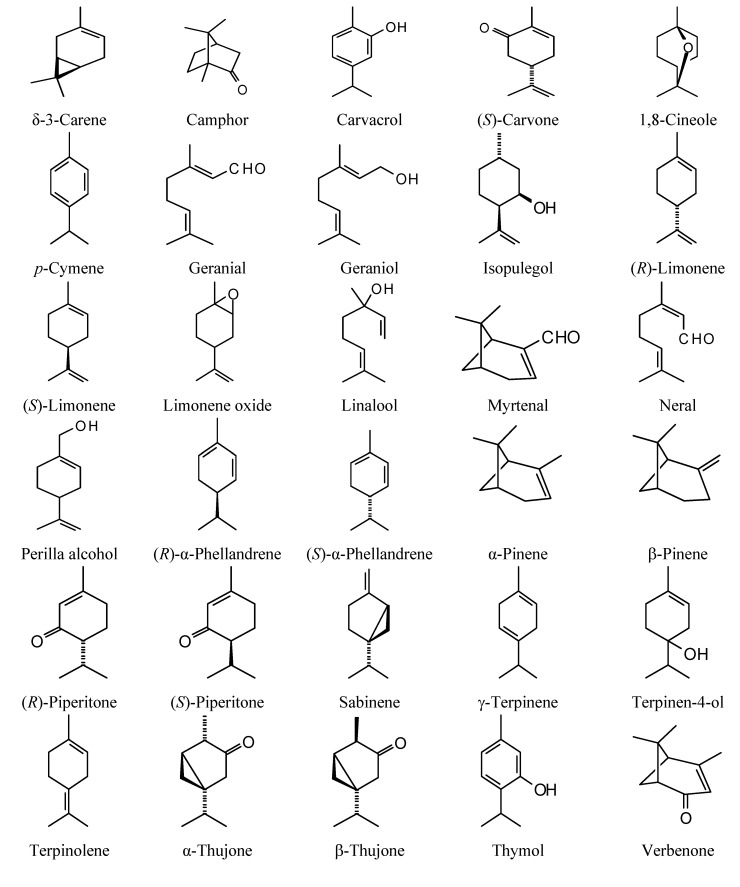
Monoterpenoids examined in this study.

**Table 1:** MolDock docking energies (kJ/mol) of monoterpenoids with *Leishmania major* protein targets.

Monoterpenoids	LmajCatB	LmajDHODH	LmajdUTPase	LmajNDKb	LmajNH	LmajNMT	LmajOPB	LmajPDE1	LmajPTR1	LmajMetRS	LmajTyrRS	LmajUGPase
δ-3-Carene	−43.8	−63.0	−50.0	−56.3	−51.0	−52.9	−62.7	−56.9	−54.9	−60.8	−63.2	−65.1
Camphor	−43.2	−61.3	−39.2	−52.4	−50.4	−50.0	−49.1	−58.1	−43.6	−52.0	−44.6	−60.9
Carvacrol	−46.5	−64.8	−58.9	−61.9	−52.7	−69.9	−66.6	−62.6	−62.1	−70.9	−66.7	−66.2
(*S*)-Carvone	−46.7	−68.9	−56.0	−63.6	−53.2	−55.8	−56.0	−63.6	−56.6	−69.2	−61.5	−66.5
1,8-Cineole	−37.1	−57.6	−37.8	−50.6	−43.5	−45.7	−42.5	−53.6	−39.7	−46.8	−45.4	−59.6
*p*-Cymene	−40.8	−55.7	−54.6	−54.8	−51.4	−63.0	−57.6	−58.1	−56.5	−61.9	−63.6	−60.7
Geranial	−52.8	−69.0	−67.3	−77.1	−65.8	−75.6	−63.8	−66.7	−72.8	−76.8	−73.7	−76.9
Geraniol	−55.9	−69.7	−68.9	−73.5	−67.5	-72.8	−68.0	−65.1	−70.2	−76.5	−72.1	−74.1
Isopulegol	−46.3	−64.8	−51.1	−59.0	−55.4	−63.6	−54.7	−57.0	−53.8	−61.0	−65.0	−62.6
(*R*)-Limonene	−46.1	−61.6	−49.6	−59.5	−47.5	−52.4	−56.4	−57.6	−57.2	−63.4	−58.4	−65.6
(*S*)-Limonene	−44.3	−58.7	−56.4	−56.2	−50.0	−65.9	−59.5	−58.6	−56.3	−64.3	−66.0	−62.0
Limonene oxide	−44.1	−65.2	−48.2	−57.7	−52.5	−52.5	−59.6	−59.0	−50.1	−59.0	−52.0	−64.8
Linalool	−52.3	−67.4	−63.9	−70.1	−62.0	−71.0	−63.2	−65.5	−65.7	−73.7	−72.5	−71.9
Myrtenal	−47.1	−64.3	−45.7	−56.5	−48.8	−51.5	−60.8	−61.8	−51.7	−57.3	−55.7	−66.2
Neral	−49.6	−72.2	−68.3	−76.6	−68.0	−75.2	−64.6	-67.3	−71.8	−75.3	−71.0	−75.6
Perilla alcohol	−49.0	−63.1	−58.2	−60.9	−52.3	−72.9	−68.1	−64.0	−63.5	−73.6	−72.9	−63.5
(*R*)-α-Phellandrene	−41.7	−61.6	−53.2	−59.8	−51.0	−53.8	−56.4	−55.9	−59.2	−66.7	−60.8	−66.4
(*S*)-α-Phellandrene	−42.2	−60.3	−54.5	−57.8	−51.9	−65.1	−58.6	−58.3	−55.0	−65.3	−64.7	−63.6
α-Pinene	−40.0	−54.9	−39.3	−52.0	−45.1	−44.9	−53.4	−53.4	−42.0	−53.4	−49.4	−60.6
β-Pinene	−39.6	−55.5	−39.8	−52.6	−47.0	−45.3	−52.9	−53.7	−41.3	−54.3	−47.5	−60.4
(*R*)-Piperitone	−45.9	−63.0	−54.9	−60.2	−51.9	−67.0	−60.2	−63.1	−62.0	−63.8	−65.0	−67.9
(*S*)-Piperitone	−46.8	−67.4	−54.6	−61.2	−50.8	−60.8	−61.2	−62.3	−59.7	−65.6	−64.9	−68.0
Sabinene	−43.7	−63.4	−54.6	−63.3	−51.4	−55.8	−58.9	−57.5	−58.4	−66.5	−62.0	−67.0
γ-Terpinene	−41.1	−57.4	−56.0	−56.0	−52.4	−65.5	−58.8	−59.7	−57.5	−63.5	−65.5	−62.0
Terpinen-4-ol	−47.5	−62.5	−57.4	−59.7	−53.2	−60.8	−58.9	−61.0	−61.7	−66.9	−69.8	−64.4
Terpinolene	−46.2	−59.8	−52.6	−55.3	−49.1	−58.8	−55.3	−58.5	−57.0	−62.8	−62.3	−61.2
α-Thujone	−48.4	−73.0	−55.8	−62.2	−51.7	−59.0	−58.6	−64.3	−63.5	−73.8	−62.1	−67.5
β-Thujone	−47.6	−69.7	−58.2	−60.2	−56.9	−59.7	−62.8	−65.0	−62.3	−71.0	−62.5	−67.9
Thymol	−45.8	−61.7	−55.7	−60.9	−52.3	−68.7	−59.2	−64.2	−62.9	−65.6	−69.5	−63.8
Verbenone	−41.4	−62.0	−44.3	−57.5	−49.4	−51.0	−48.7	−58.2	−47.0	−53.0	−52.7	−63.2

**Table 2:** MolDock docking energies (kJ/mol) of monoterpenoids with *Leishmania donovani* and *L. mexicana* protein targets.

Monoterpenoids	LdonCatB	LdonCyp	LdonDHODH	LdonNMT	LmexGAPDH	LmexGPDH	LmexPGI	LmexPMM	LmexPYK	LmexPYK	LmexPYK	LmexTIM
									Site 1	Site 2	Site 3	
δ-3-Carene	−50.9	−56.1	−59.4	−49.5	−53.7	−59.1	−43.5	−51.8	−58.9	−50.8	−59.4	−58.0
Camphor	−42.7	−48.0	−62.2	−47.6	−44.4	−58.8	−40.9	−50.4	−50.2	−45.7	−55.1	−45.9
Carvacrol	−58.3	−63.8	−57.9	−64.9	−56.0	−61.2	−48.6	−57.9	−63.9	−53.4	−65.5	−56.3
(*S*)-Carvone	−54.0	−65.6	−60.3	−59.7	−49.6	−61.2	−49.5	−58.3	−62.4	−55.5	−62.9	−54.1
1,8-Cineole	−36.3	−46.8	−53.6	−38.0	−39.8	−51.1	−37.6	−47.1	−52.5	−42.7	−50.3	−48.3
*p*-Cymene	−51.7	−54.5	−51.4	−54.7	−48.4	−56.1	−50.2	−52.9	−58.0	−53.4	−60.4	−55.2
Geranial	−68.7	−67.2	−65.2	−75.0	−66.6	−70.4	−74.4	−63.9	−71.1	−61.2	−75.2	−71.4
Geraniol	−69.3	−68.2	−65.8	−71.7	−64.2	−72.4	−70.1	−63.1	−71.3	−61.6	−75.5	−70.1
Isopulegol	−52.9	−61.1	−57.6	−65.2	−57.9	−60.9	−44.9	−57.6	−63.1	−55.3	−59.3	−59.4
(*R*)-Limonene	−52.1	−58.6	−57.2	−51.8	−49.5	−58.1	−43.4	−53.3	−56.9	−51.1	−58.1	−53.1
(*S*)-Limonene	−54.5	−57.2	−50.5	−62.3	−51.9	−58.7	−43.5	−55.3	−61.3	−54.8	−62.2	−56.9
Limonene oxide	−46.2	−58.4	−63.9	−33.4	−54.1	−61.1	−44.3	−53.9	−58.3	−52.2	−61.2	−57.6
Linalool	−65.1	−65.1	−70.5	−65.6	−61.4	−67.8	−58.6	−66.9	−68.7	−60.1	−74.8	−65.9
Myrtenal	−47.7	−59.2	−60.1	−36.7	−49.1	−60.2	−45.0	−52.6	−57.7	−50.4	−55.8	−53.6
Neral	−64.4	−69.2	−70.5	−71.7	−66.1	−72.9	−58.7	−63.2	−71.8	−65.2	−74.2	−65.5
Perilla alcohol	−58.7	−65.2	−57.5	−71.0	−60.3	−63.1	−50.4	−57.7	−67.0	−57.8	−68.7	−60.7
(*R*)-α-Phellandrene	−52.8	−58.6	−58.9	−54.9	−53.6	−57.0	−52.9	−51.0	−60.9	−50.9	−59.7	−55.8
(*S*)-α-Phellandrene	−53.4	−59.1	−55.0	−59.2	−52.5	−57.2	−47.7	−53.3	−61.9	−52.7	−62.1	−55.2
α-Pinene	−40.5	−52.5	−56.2	−37.5	−43.7	−53.0	−39.9	−47.1	−50.7	−42.8	−49.6	−46.4
β-Pinene	−39.7	−53.2	−57.4	−39.5	−45.2	−52.4	−39.2	−47.3	−49.9	−41.8	−50.7	−48.2
(*R*)-Piperitone	−57.5	−66.1	−54.1	−65.1	−57.1	−60.2	−50.4	−58.0	−62.7	−54.8	−61.9	−58.4
(*S*)-Piperitone	−55.3	−63.2	−64.2	−59.3	−53.8	−61.2	−46.7	−57.4	−63.8	−56.1	−62.2	−56.4
Sabinene	−49.3	−59.0	−57.9	−59.4	−50.9	−59.9	−46.0	−52.1	−61.0	−51.6	−61.2	−55.8
γ-Terpinene	−53.1	−56.2	−54.0	−59.5	−50.6	−57.9	−49.2	−54.0	−60.4	−55.7	−62.2	−56.8
Terpinen-4-ol	−52.4	−63.8	−55.1	−64.6	−51.4	−61.7	−51.8	−56.9	−64.5	−56.9	−65.6	−58.7
Terpinolene	−54.1	−57.8	−52.6	−57.7	−48.0	−57.2	−45.3	−51.9	−58.9	−51.3	−58.9	−54.7
α-Thujone	−51.3	−63.8	−63.6	−53.2	−56.1	−65.0	−49.8	−56.6	−65.2	−54.2	−62.2	−57.7
β-Thujone	−56.3	−61.6	−60.7	−58.1	−53.2	−65.6	−48.2	−58.0	−65.1	−54.7	−61.0	−59.9
Thymol	−55.8	−61.3	−54.5	−64.1	−52.1	−62.3	−47.2	−56.6	−65.2	−56.3	−61.7	−57.1
Verbenone	−40.9	−58.0	−61.6	−45.4	−48.1	−59.6	−42.4	−52.2	−56.9	−49.2	−58.0	−48.9

**Table 3:** MolDock docking energies (kJ/mol) of monoterpenoids with *Leishmania infantum* protein targets.

Monoterpenoids	LinfCYP51	LinfGLO2	LinfPnC1	LinfTDR1	LinfTR
δ-3-Carene	−50.4	−48.0	−66.1	−45.9	−56.3
Camphor	−46.1	−37.8	−56.8	−43.8	−52.1
Carvacrol	−59.5	−53.8	−71.0	−53.5	−60.9
(*S*)-Carvone	−54.9	−47.9	−73.6	−52.1	−57.6
1,8-Cineole	−43.3	−34.9	−54.4	−39.5	−50.6
*p*-Cymene	−53.2	−52.1	−64.1	−49.6	−57.8
Geranial	−63.0	−61.7	−75.0	−64.3	−70.1
Geraniol	−64.5	−59.1	−74.8	−64.2	−69.1
Isopulegol	−50.8	−49.6	−67.6	−49.8	−58.6
(*R*)-Limonene	−51.6	−51.4	−68.8	−48.4	−54.0
(*S*)-Limonene	−53.0	−52.7	−65.2	−49.1	−60.0
Limonene oxide	−51.6	−46.6	−64.8	−45.4	−54.0
Linalool	−61.6	−56.1	−72.7	−62.2	−66.1
Myrtenal	−48.7	−45.2	−63.2	−47.6	−54.1
Neral	−60.7	−57.1	−76.4	−61.4	−68.1
Perilla alcohol	−61.9	−60.0	−69.3	−57.8	−65.5
(*R*)-α-Phellandrene	−51.1	−50.9	−68.1	−48.4	−56.1
(*S*)-α-Phellandrene	−53.9	−51.2	−66.3	−50.6	−59.4
α-Pinene	−42.4	−38.4	−56.9	−41.0	−47.3
β-Pinene	−43.0	−37.5	−56.9	−41.6	−48.3
(*R*)-Piperitone	−54.9	−50.2	−70.9	−51.3	−62.8
(*S*)-Piperitone	−54.2	−51.6	−73.0	−54.2	−60.3
Sabinene	−52.9	−53.8	−68.2	−54.4	−58.8
γ-Terpinene	−54.8	−53.0	−65.8	−49.7	−59.2
Terpinen-4-ol	−53.2	−51.3	−69.4	−50.4	−63.3
Terpinolene	−52.4	−51.3	−67.6	−49.6	−59.2
α-Thujone	−53.8	−53.9	−74.5	−54.0	−60.4
β-Thujone	−54.5	−50.8	−69.4	−52.0	−64.7
Thymol	−60.2	−53.5	−71.5	−52.9	−59.6
Verbenone	−50.2	−42.2	−63.0	−48.0	−50.5

## 2. Results and Discussion

### 2.1. Monoterpenoid Docking

The structures of the monoterpenoids examined in this study are shown in Figure 1, while the corresponding docking energies are summarized in Table 1, Table 2 and Table 3. The overall strongest docking monoterpenoid ligands were the acyclic geranial, geraniol, and neral, probably owing to their flexibility. These ligands, however, did not show docking selectivity to any of the *Leishmania* protein targets, but rather docked strongly to most of the proteins investigated. The protein targets that showed predominantly strong docking by monoterpenoids were *L. major* uridine diphosphate-glucose pyrophosphorylase (LmajUGPase), *L. major* methionyl t-RNA synthetase (LmajMetRS), and *L. infantum* nicotinamidase (LinfPnC1). Geranial had a docking energy of −76.9 kJ/mol with LmajUGPase, comparable in docking energy with several other proteins. Both enantiomers of piperitone showed significantly stronger docking to Lmaj UGPase (−68.0 kJ/mol) than the other targets, suggesting selectivity for that protein. Geranial was also the strongest docking ligand with LmajMetRS (−76.8 kJ/mol), but perilla alcohol (−73.6 kJ/mol) was selective for that protein target. Carvone, piperitone, and α-thujone showed significantly selective docking to LinfPnC1 (docking energies less than −73 kJ/mol). Interestingly, although the monoterpenoids showed a docking propensity for LinfPnC1, higher terpenoids (sesquiterpenoids, diterpenoids, and triterpenoids) showed very little inclination to dock to this protein, generally with positive docking energies (see below).

Monoterpenoids represents a very small percentage of terpene-derived compounds that have been reported to have antileishmanial activity, and the docking energies of monoterpenoids were generally weaker than those obtained for limonoids, withanolides, triterpenoids, steroids and diterpenoids with these same targets (see below). Their docking energies were much higher than the energies obtained for the co-crystallized ligands of those protein targets. The higher docking energies of these compounds correlate with their small size (and molecular weight), and the minimal intermolecular interactions they are able to have with the protein targets. So, comparatively, it appears that monoterpenoids will not be prime leads for structure-based antileishmanial drug discovery. However, they may be useful in fragment-based drug discovery [62,63]. Additionally, several terpene-derived compounds are used in topical formulations. Therefore, those monoterpenoids that have antileishmanial activity and no reported toxicity at physiologically relevant concentration/dosage should be evaluated as possible components of topical polytherapy for leishmaniasis.

### 2.2. Sesquiterpenoid Docking

**Figure 2:**
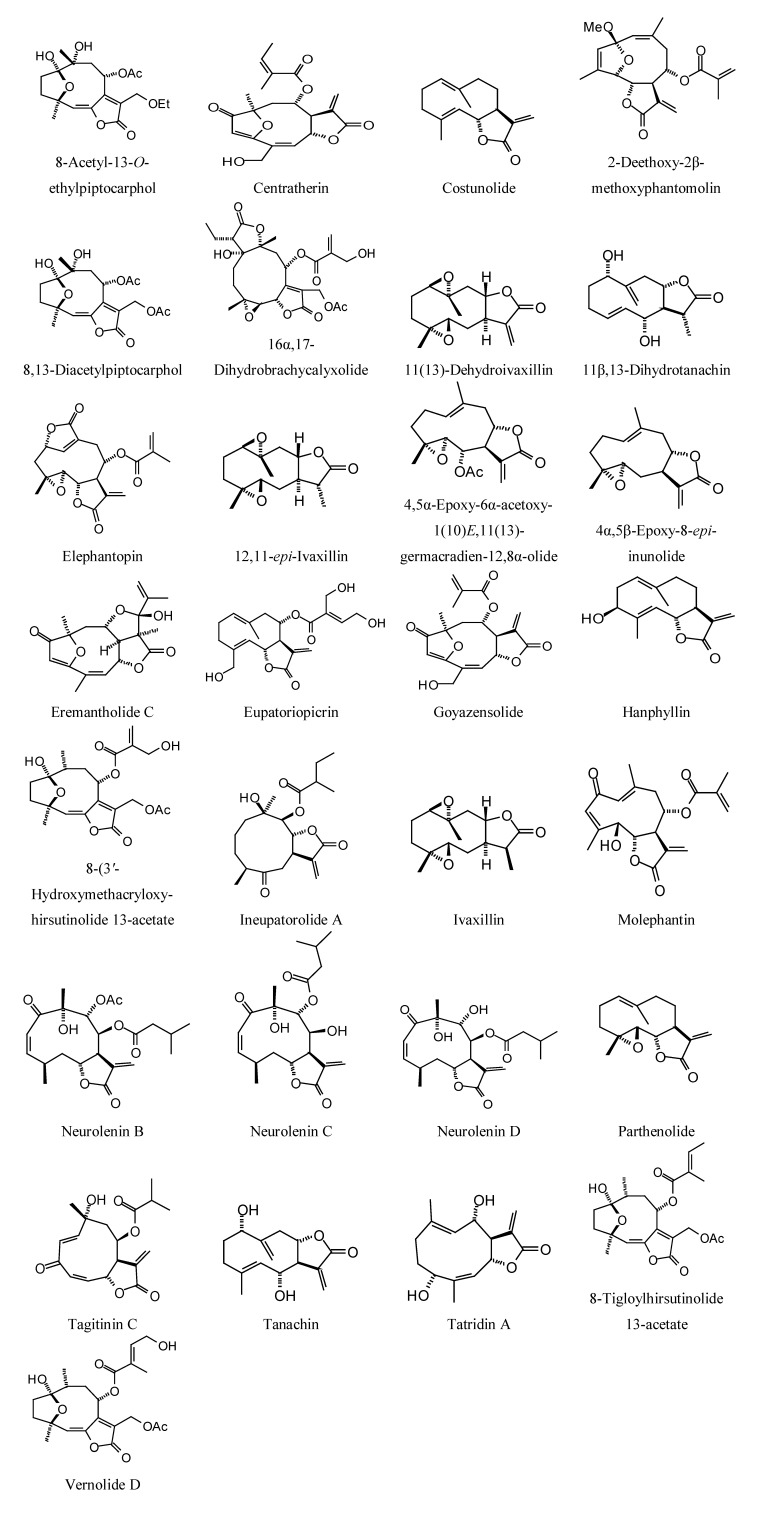
Germacranolide sesquiterpenoids examined in this work.

**Figure 3:**
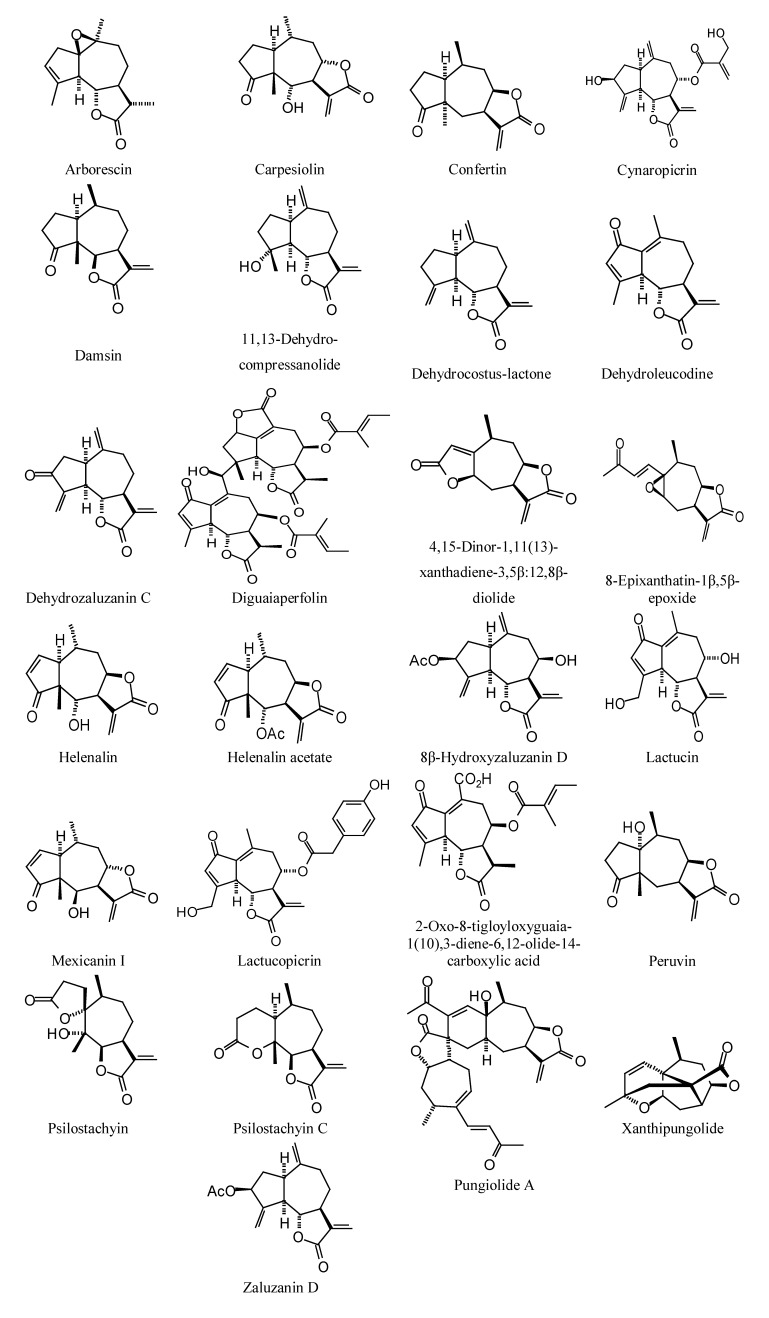
Guaianolide sesquiterpenoids examined in this work.

**Figure 4:**
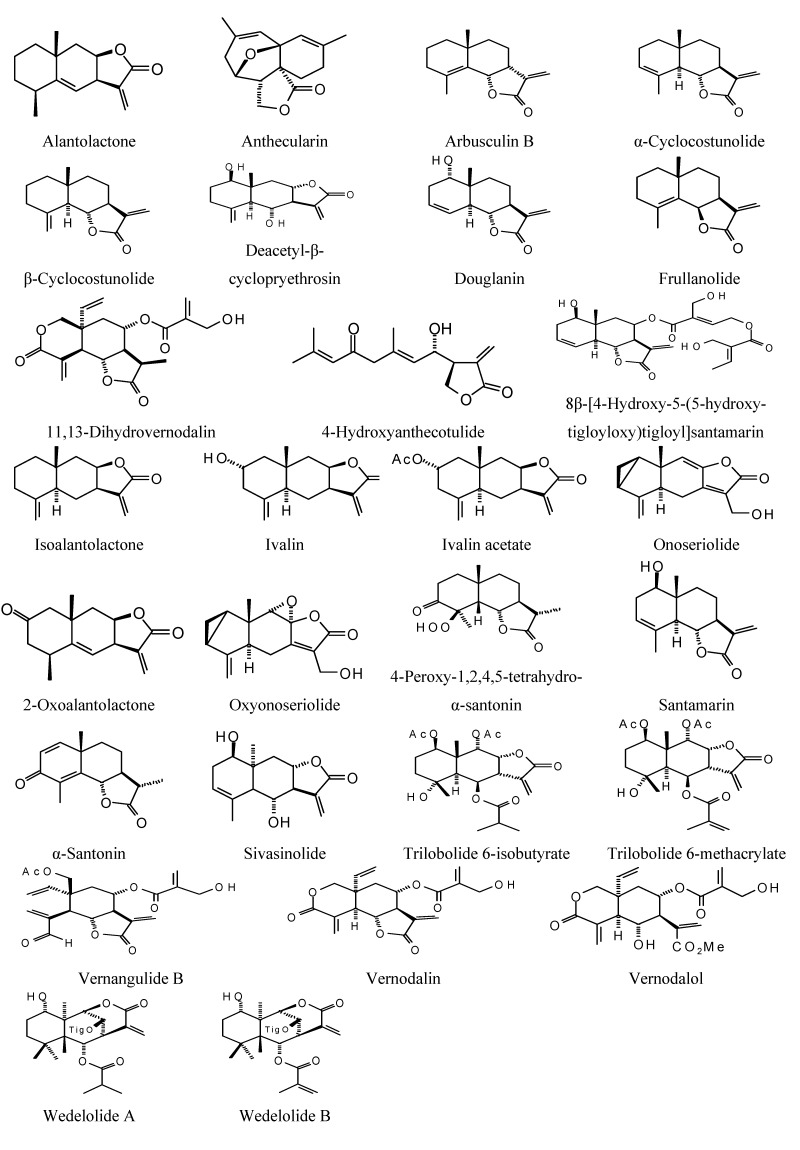
Eudesmanolide sesquiterpenoids examined in this work.

**Figure 5:**
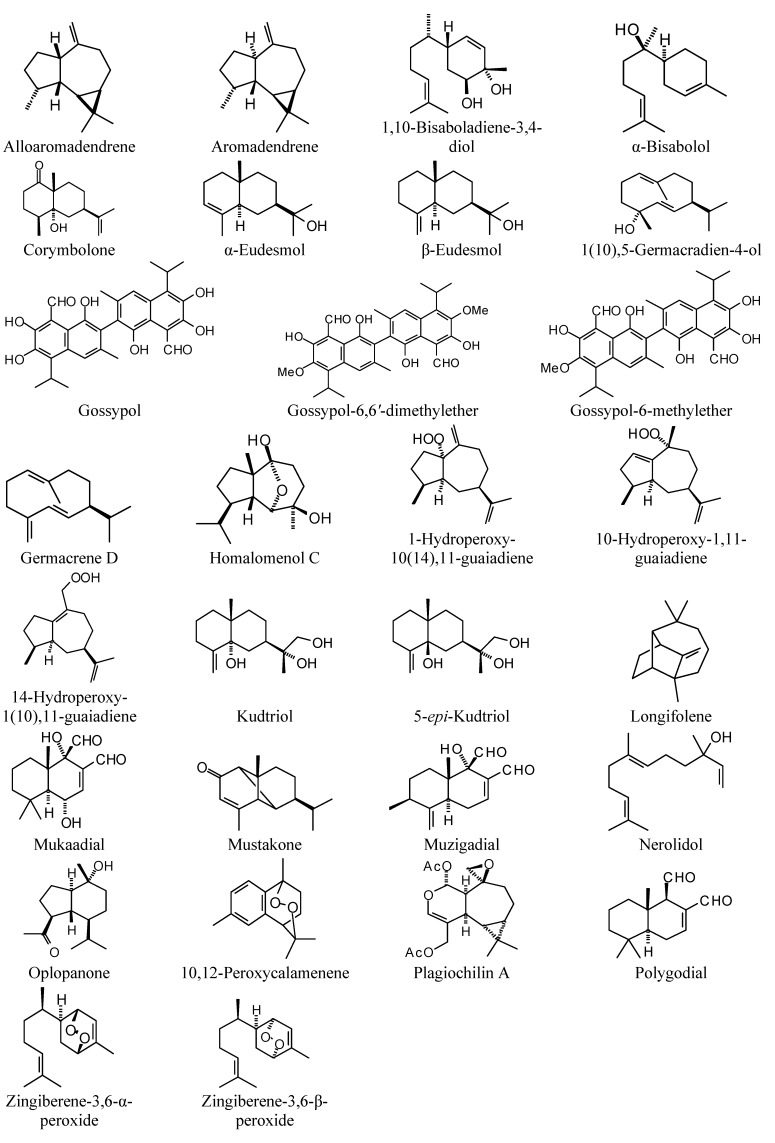
Miscellaneous sesquiterpenoids examined in this work.

Sesquiterpenoids examined in this work are shown in Figure 2, Figure 3, Figure 4 and Figure 5; docking energies of sesquiterpenoids are summarized in Table 4, Table 5, Table 6, Table 7, Table 8, Table 9, Table 10, Table 11, Table 12, Table 13, Table 14 and Table 15. The germacranolide sesquiterpenoids exhibited the overall strongest docking energies toward the *Leishmania* protein targets, with 16,17-dihydrobrachycalyxolide the strongest-docking germacranolide. This ligand showed docking selectivity toward LmajMetRS (docking energy = −152.9 kJ/mol) and *L. mexicana* phosphor-mannomutase (LmexPMM) (docking energy = −136.3 kJ/mol). The two proteins most selectively targeted by the germacranolides in terms of docking energies were LmajMetRS and *L. major* dihydroorotate dehydrogenase (LmajDHODH). Although most germacranolides did not dock with LinfPnC1, tatridin A did show docking selectivity for this protein target. Based on molecular weight, the strongest-docking germacranolide was 4α,5β-epoxy-8-*epi*-inunolide, and this ligand showed docking selectivity toward both LmajMet RS and LmajDHODH.

**Table 4:** MolDock docking energies (kJ/mol) of germacranolide sesquiterpenoids with *Leishmania major* protein targets.

Germacranolides	LmajCatB	LmajDHODH	LmajdUTPase	LmajNDKb	LmajNH	LmajNMT	LmajOPB	LmajPDE1	LmajPTR1	LmajMetRS	LmajTyrRS	LmajUGPase
8-Acetyl-13-*O*-ethylpiptocarphol	−90.8	−113.1	−99.0	−87.6	−84.0	−108.3	−114.1	−98.2	−105.3	−127.1	−99.1	−105.1
Centratherin	−85.4	−115.1	−92.2	−95.7	−96.6	−106.8	−101.2	−102.8	−101.5	−125.8	−112.9	−108.0
Costunolide	−62.3	−86.5	−73.8	−71.8	−68.1	−79.8	−78.4	−75.0	−79.9	−75.6	−76.3	−81.8
2-Deethoxy-2β-methoxyphantomolin	−94.9	−121.1	−96.1	−85.7	−88.2	−95.4	−96.6	−101.1	−102.4	−114.6	−105.2	−95.3
8,13-Diacetylpiptocarphol	−92.2	−115.1	−105.5	−93.4	−88.9	−106.7	−100.2	−102.6	−107.3	−131.5	−99.6	−110.6
16α,17-Dihydrobrachycalyxolide	−100.1	−129.1	−112.5	−117.3	−111.0	−121.5	−100.2	−122.8	−105.0	−152.9	−117.7	−115.7
11(13)-Dehydroivaxillin	−78.0	−108.3	−78.6	−80.6	−81.5	−84.4	−91.4	−81.9	−89.1	−101.9	−88.3	−95.7
11β,13-Dihydrotanachin	−66.9	−96.3	−73.5	−67.8	−79.6	−78.7	−77.2	−75.3	−66.8	−77.2	−78.9	−89.6
Elephantopin	−86.7	−114.6	−98.8	−90.3	−91.5	−105.0	−97.8	−106.8	−103.9	−119.3	−105.0	−105.7
11-*epi*-Ivaxillin	−76.9	−109.9	−80.5	−81.7	−83.5	−85.1	−94.9	−86.9	−90.1	−102.4	−80.7	−85.9
4,5α-Epoxy-6α-acetoxy-1(10)*E*,11(13)-germacradien-12,8α-olide	−77.1	−92.4	−94.2	−80.4	−82.5	−99.6	−95.4	−89.1	−86.5	−94.8	−87.9	−92.5
4α,5β-Epoxy-8-*epi*-inunolide	−74.1	−100.5	−76.5	−76.8	−80.3	−82.5	−86.2	−81.0	−88.5	−99.1	−84.8	−88.2
Eremantholide C	−89.2	−103.4	−86.5	−100.2	−86.2	−96.6	−95.3	−98.1	−83.1	−100.9	−87.2	−101.8
Eupatoriopicrin	−83.7	−107.8	−106.2	−99.2	−104.9	−114.5	−111.1	−105.7	−100.3	−120.7	−97.3	−102.0
Goyazensolide	−73.5	−107.0	−90.0	−97.6	−93.1	−108.4	−101.1	−107.7	−98.2	−116.4	−110.9	−107.4
Hanphyllin	−71.2	−91.5	−73.8	−76.9	−77.7	−80.8	−79.5	−76.3	−80.7	−85.7	−84.5	−84.4
8-(3*'*-Hydroxymethacryloxy)-hirsutinolide 13-acetate	−94.0	−107.3	−106.8	−105.6	−104.4	−107.8	−105.0	−110.7	−109.6	−134.9	−105.1	−108.6
Ineupatorolide A	−83.1	−117.3	−90.0	−92.4	−91.0	−98.7	−100.4	−97.2	−112.8	−119.4	−112.1	−105.9
Ivaxillin	−76.6	−104.2	−79.3	−78.3	−75.2	−83.2	−90.6	−82.6	−83.4	−98.1	−82.8	−88.3
Molephantin	−80.8	−105.0	−87.4	−80.0	−87.4	−101.6	−87.9	−99.0	−94.7	−106.0	−99.3	−91.9
Neurolenin B	−86.0	−116.4	−88.0	−100.0	−88.7	−95.6	−77.3	−103.0	−86.7	−99.8	−90.3	−99.5
Neurolenin C	−82.7	−116.3	−93.0	−94.4	−86.2	−102.1	−89.6	−95.8	−102.6	−112.6	−99.0	−117.6
Neurolenin D	−90.5	−111.1	−95.9	−99.2	−92.4	−97.5	−87.9	−101.4	−94.6	−104.7	−90.7	−100.6
Parthenolide	−65.8	−98.7	−80.6	−75.2	−81.4	−78.8	−83.1	−80.1	−84.4	−89.3	−79.3	−89.6
Tagitinin C	−87.4	−102.0	−90.4	−89.0	−96.1	−96.8	−95.1	−105.2	−102.8	−111.3	−94.5	−102.2
Tanachin	−71.3	−94.1	−71.1	−78.3	−76.4	−80.6	−82.5	−78.9	−75.1	−91.4	−80.0	−86.8
Tatridin A	−74.1	−96.6	−69.7	−75.7	−76.3	−80.7	−88.6	−78.4	−89.8	−91.2	−80.8	−90.7
8-Tigloylhirsutinolide 13-acetate	−93.8	−116.4	−105.4	−98.0	−99.9	−114.6	−110.3	−113.6	−113.6	−124.8	−105.5	−119.9
Vernolide D	−97.8	−124.5	−110.9	−106.3	−121.8	−117.9	−112.4	−118.3	−114.4	−128.1	−111.8	−121.5

**Table 5:** MolDock docking energies (kJ/mol) of germacranolide sesquiterpenoids with *Leishmania donovani* and *L. mexicana* protein targets.

Germacranolides	LdonCatB	LdonCyp	LdonDHODH	LdonNMT	LmexGAPDH	LmexGPDH	LmexPGI	LmexPMM	LmexPYK	LmexPYK	LmexPYK	LmexTIM
									Site 1	Site 2	Site 2	
8-Acetyl-13-O-ethylpiptocarphol	−92.9	−97.6	−79.4	−84.2	−93.2	−99.2	−88.6	−113.3	−102.5	−94.9	−99.2	−95.1
Centratherin	−89.0	−−88.7	−86.0	−90.9	−82.2	−126.6	−87.5	−108.9	−100.3	−96.0	−107.5	−100.6
Costunolide	−78.9	−80.5	−61.1	−65.8	−63.5	−80.6	−64.5	−76.0	−84.8	−73.8	−87.1	−77.6
2-Deethoxy-2β-methoxyphantomolin	−101.6	−88.0	−80.7	−87.6	−88.7	−110.2	−87.4	−110.3	−103.1	−85.8	−105.8	−79.2
8,13-Diacetylpiptocarphol	−86.4	−99.7	−88.0	−92.8	−96.2	−102.6	−97.4	−118.1	−103.0	−100.4	−94.7	−100.0
16α,17-Dihydrobrachycalyxolide	−104.2	−104.4	−100.0	−96.1	−108.9	−130.0	−107.9	−136.3	−125.1	−108.8	−110.3	−114.6
11(13)-Dehydroivaxillin	−78.1	−85.0	−64.3	−83.7	−72.9	−99.4	−68.0	−90.6	−91.0	−79.8	−106.6	−81.8
11β,13-Dihydrotanachin	−73.4	−77.2	−52.3	−75.2	−69.6	−81.9	−67.2	−79.1	−82.3	−74.9	−78.1	−66.1
Elephantopin	−83.4	−90.2	−86.6	−88.1	−82.6	−116.1	−89.7	−115.8	−110.5	−92.9	−103.0	−92.4
11-*epi*-Ivaxillin	−77.0	−84.8	−66.5	−88.4	−74.1	−96.3	−66.4	−88.9	−93.2	−79.4	−106.9	−78.0
4,5α-Epoxy-6α-acetoxy-1(10)*E*,11(13)-germacradien-12,8α-olide	−77.0	−90.7	−66.4	−82.3	−73.0	−98.9	−79.5	−91.7	−97.8	−88.0	−88.3	−81.5
4α,5β-Epoxy-8-*epi*-inunolide	−73.3	−85.8	−76.9	−78.8	−67.6	−90.7	−62.3	−86.8	−87.8	−75.2	−95.4	−78.6
Eremantholide C	−87.6	−98.8	−86.4	−76.2	−77.4	−114.2	−79.9	−103.2	−102.0	−104.2	−85.1	−94.2
Eupatoriopicrin	−87.0	−94.3	−98.3	−96.1	−99.5	−116.5	−92.5	−102.7	−119.4	−103.5	−96.0	−97.7
Goyazensolide	−86.2	−85.7	−83.9	-82.3	−87.8	−124.1	−88.4	−107.9	−99.0	−95.9	−103.4	−98.7
Hanphyllin	−85.4	−85.0	−32.6	−67.3	−69.8	−81.3	−66.5	−80.5	−86.6	−75.1	−87.5	−83.5
8-(3*'*-Hydroxymethacryloxy-hirsutinolide 13-acetate	−98.6	−98.4	−80.0	−98.5	−88.4	−113.9	−96.1	−117.6	−112.4	−101.4	−110.7	−103.4
Ineupatorolide A	−97.4	−95.2	−94.2	−86.5	−72.5	−102.4	−82.2	−99.7	−100.1	−91.2	−100.4	−104.6
Ivaxillin	−73.9	−84.9	−74.6	−78.3	−77.9	−90.4	−68.4	−91.3	−93.5	−77.9	−105.0	−80.7
Molephantin	−87.9	−94.5	−36.3	−89.4	−79.4	−92.9	−78.8	−96.6	−106.8	−88.8	−99.8	−99.9
Neurolenin B	−88.1	−82.6	−66.6	−76.5	−77.7	−98.4	−80.1	−110.7	−103.3	−92.0	−100.4	−85.9
Neurolenin C	−93.6	−89.8	−49.0	−94.7	−86.5	−94.4	−79.3	−106.0	−95.0	−89.9	−91.6	−95.2
Neurolenin D	−94.8	−78.1	−77.6	−95.3	−87.1	−94.1	−80.8	−111.4	−97.7	−89.1	−92.4	−92.0
Parthenolide	−73.2	−81.2	−75.6	−75.4	−63.6	−89.6	−64.8	−84.4	−88.5	−79.3	−88.9	−78.1
Tagitinin C	−88.0	−84.6	−52.0	−87.5	−85.2	−92.8	-78.0	−112.3	−101.4	−91.5	−98.0	−91.4
Tanachin	−77.7	−82.1	−58.7	−71.0	−70.6	−89.8	−69.9	−81.7	−86.0	−83.4	−77.9	−83.4
Tatridin A	−84.7	−84.5	−67.6	−72.8	−59.3	−85.7	−61.6	−83.8	−83.5	−81.2	−89.7	−76.9
8-Tigloylhirsutinolide 13-acetate	−104.5	−83.9	−94.8	−97.3	−91.0	−110.3	−99.2	−117.5	−111.3	−98.3	−108.0	−102.8
Vernolide D	−107.5	−94.3	−89.6	−102.3	−94.8	−120.3	−96.8	−125.4	−117.4	−113.7	−99.8	−109.1

**Table 6:** MolDock docking energies (kJ/mol) of germacranolide sesquiterpenoids with *Leishmania infantum* protein targets.

Germacranolides	LinfCYP51	LinfGLO2	LinfPnC1	LinfTDR1	LinfTR
8-Acetyl-13-*O*-ethylpiptocarphol	−100.4	−89.8	no dock	−82.3	−99.7
Centratherin	−109.0	−87.1	no dock	−88.5	−104.9
Costunolide	−74.4	−63.6	no dock	−69.9	−71.2
2-Deethoxy-2β-methoxyphantomolin	−101.4	−83.7	no dock	−83.2	−95.3
8,13-Diacetylpiptocarphol	−97.8	−88.8	no dock	−91.7	−101.6
16α,17-Dihydrobrachycalyxolide	−126.4	−108.8	no dock	−104.2	−112.9
11(13)-Dehydroivaxillin	−81.4	−69.4	−86.5	−79.4	−81.2
11β,13-Dihydrotanachin	−73.7	−63.2	−86.6	−72.2	−76.3
Elephantopin	−110.7	−90.5	no dock	−92.2	−104.4
11-*epi*-Ivaxillin	−83.4	−73.9	−79.8	−77.7	−81.2
4,5α-Epoxy-6α-acetoxy-1(10)*E*,11(13)-germacradien-12,8α-olide	−92.9	−79.5	no dock	−80.9	−89.6
4α,5β-Epoxy-8-*epi*-inunolide	−77.1	−67.0	−80.3	−70.2	−77.2
Eremantholide C	−91.3	−70.7	no dock	−81.6	−95.3
Eupatoriopicrin	−100.1	−91.3	no dock	−96.5	−105.1
Goyazensolide	−104.7	−83.9	no dock	−84.3	−98.8
Hanphyllin	−79.3	−64.9	−64.6	−75.7	−76.4
8-(3*'*-Hydroxymethacryloxy)-hirsutinolide 13-acetate	−120.2	−90.6	no dock	−100.1	−114.7
Ineupatorolide A	−102.5	−84.2	no dock	−89.1	−95.0
Ivaxillin	−82.2	−70.1	no dock	−75.0	−84.9
Molephantin	−101.0	−82.4	no dock	−82.3	−101.2
Neurolenin B	−95.7	−76.0	no dock	−81.0	−92.5
Neurolenin C	−95.9	−76.9	no dock	−82.0	−93.3
Neurolenin D	−99.7	−76.0	no dock	−80.7	−86.9
Parthenolide	−82.1	−63.5	−84.6	−75.2	−76.9
Tagitinin C	−97.0	−79.4	no dock	−77.6	−90.7
Tanachin	−82.0	−61.0	−62.5	−73.7	−74.5
Tatridin A	−87.0	−65.2	−97.2	−70.9	−77.8
8-Tigloylhirsutinolide 13-acetate	−124.6	−88.1	no dock	−91.5	−109.1
Vernolide D	−131.9	−94.9	no dock	−100.0	−110.4

**Table 7:** MolDock docking energies (kJ/mol) of guaianolide sesquiterpenoids with *Leishmania major* protein targets.

Guaianolides	LmajCatB	LmajDHODH	LmajdUTPase	LmajNDKb	LmajNH	LmajNMT	LmajOPB	LmajPDE1	LmajPTR1	LmajMetRS	LmajTyrRS	LmajUGPase
Arborescin	−66.2	−93.6	−78.3	−83.0	−72.7	−78.2	−85.3	−77.7	−95.1	−90.4	−91.8	−79.5
Carpesiolin	−73.3	−97.3	−73.5	−79.0	−70.7	−79.4	−84.1	−75.6	−87.3	−86.7	−86.0	−88.0
Confertin	−62.4	−90.0	−72.4	−73.5	−75.8	−72.7	−81.5	−70.1	−74.8	−94.4	−78.3	−83.7
Cynaropicrin	−78.6	−117.2	−104.1	−101.0	−106.4	−109.8	−97.6	−109.4	−103.0	−115.0	−107.2	−108.6
Damsin	−64.9	−102.1	−74.1	−77.0	−77.4	−83.2	−79.4	−78.2	−74.8	−89.1	−77.5	−85.1
11,13-Dehydrocompressanolide	−72.6	−94.1	−77.6	−78.6	−74.9	−76.8	−78.7	−80.1	−85.0	−93.3	−81.7	−92.6
Dehydrocostuslactone	−63.2	−86.9	−76.2	−73.1	−72.7	−69.5	−75.1	−73.5	−82.1	−86.5	−83.8	−79.8
Dehydroleucodine	−66.9	−89.5	−85.2	−84.2	−80.6	−79.9	−83.7	−84.2	−94.2	−90.9	−93.3	−83.2
Dehydrozaluzanin C	−73.3	−90.4	−74.9	−78.7	−69.8	−79.0	−86.3	−77.7	−85.4	−92.2	−88.4	−85.4
Diguaiaperfolin	−127.8	−154.5	−130.6	−116.8	−135.7	−129.4	−114.3	−129.7	−124.3	−129.9	−144.6	−151.8
4,15-Dinor-1,11(13)-xanthadiene-3,5β:12,8β-diolide	−76.3	−92.7	−88.4	−83.9	−70.7	−80.2	−87.5	−84.9	−95.0	−95.8	−83.4	−92.6
8-Epixanthatin-1β,5β-epoxide	−78.0	−108.0	−77.8	−89.0	−84.5	−88.3	−87.1	−88.3	−92.0	−106.8	−89.5	−95.4
Helenalin	−67.2	−92.8	−74.6	−74.6	−80.7	−77.5	−80.1	−79.0	−84.0	−86.4	−87.5	−87.2
Helenalin acetate	−72.4	−88.8	−84.1	−81.6	−86.2	−88.9	−92.6	−91.0	−76.8	−98.7	−90.3	−89.8
8β-Hydroxyzaluzanin D	−81.2	−102.8	−95.6	−87.0	−89.7	−91.1	−98.7	−90.2	−88.3	−94.1	−93.9	−98.9
Lactucin	−68.8	−100.7	−84.2	−91.9	−93.4	−87.9	−89.7	−91.7	−104.1	−92.2	−103.6	−89.3
Lactucopicrin	−97.7	−123.3	−113.8	−107.4	−110.9	−112.6	−116.8	−122.0	−129.9	−137.7	−116.5	−120.1
Mexicanin I	−75.9	−92.6	−77.9	−80.4	−79.7	−80.1	−83.8	−76.5	−83.8	−88.1	−89.1	−87.0
2-Oxo-8-tigloyloxyguaia-1(10),3-diene-6,12-olide-14-carboxylic acid	−82.1	−117.0	−99.2	−96.9	−95.9	−96.7	−90.1	−105.0	−108.3	−114.1	−103.1	−110.8
Peruvin	−62.0	−91.6	−71.8	−72.0	−78.4	−81.3	−79.5	−76.2	−79.1	−85.4	−78.2	−101.9
Psilostachyin	−71.8	−89.5	−75.0	−83.3	−75.7	−78.7	−78.1	−79.8	−76.7	−90.3	−81.7	−87.2
Psilostachyin C	−72.4	−91.5	−74.3	−75.8	−79.5	−72.8	−82.2	−78.3	−79.9	−92.5	−82.3	−89.7
Pungiolide A	−91.6	−96.7	−96.8	−116.1	−106.0	−109.6	−111.8	−122.8	−108.3	−122.6	−107.3	−117.5
Xanthipungolide	−52.4	−78.4	−50.0	−55.4	−62.7	−64.5	−62.6	−66.8	−48.3	−69.8	−60.4	−74.8
Zaluzanin D	−68.6	−103.0	−93.1	−86.7	−80.0	−93.1	−90.8	−93.7	−89.1	−96.5	−97.1	−95.3

**Table 8:** MolDock docking energies (kJ/mol) of guaianolide sesquiterpenoids with *Leishmania donovani* and *L. mexicana* protein targets.

Guaianolides	LdonCatB	LdonCyp	LdonDHODH	LdonNMT	LmexGAPDH	LmexGPDH	LmexPGI	LmexPMM	LmexPYK	LmexPYK	LmexPYK	LmexTIM
									Site 1	Site 2	Site 3	
Arborescin	−74.7	−82.7	−71.6	−73.6	−68.3	−−91.9	−65.5	−82.7	−88.1	−75.1	−86.5	−73.8
Carpesiolin	−76.7	−78.5	−61.5	−69.7	−66.5	−91.8	−61.9	−83.4	−85.7	−88.6	−94.1	−82.1
Confertin	−67.1	−85.2	−12.0	−68.1	−62.1	-85.1	−61.5	−79.0	−85.9	−77.1	−82.1	−70.8
Cynaropicrin	−80.5	−99.7	−57.3	−92.8	−88.0	−121.8	−97.2	−114.7	−127.0	−91.8	−94.1	−90.1
Damsin	−73.3	−88.7	−0.9	−73.6	−69.4	−85.7	−64.6	−79.5	−88.1	−79.9	-84.8	−68.3
11,13-Dehydrocompressanolide	−74.2	−87.8	−73.4	−72.8	−70.3	−93.1	−64.9	−86.0	−84.0	−82.1	−92.3	−79.6
Dehydrocostuslactone	−71.2	−83.5	-64.8	−66.8	−60.4	−84.8	−57.7	−76.1	−82.5	−74.6	−88.7	−69.5
Dehydroleucodine	−78.0	−84.2	−63.9	−72.1	−73.5	−88.0	−66.2	−83.0	−91.4	−74.7	−89.7	−72.3
Dehydrozaluzanin C	−74.4	−86.1	−79.0	−76.0	−68.3	−91.0	−70.8	−82.9	−85.4	−80.5	−99.5	−77.9
Diguaiaperfolin	−126.1	−124.8	−121.6	−135.5	−122.1	−138.1	−129.7	−145.6	−146.9	−141.4	−138.3	−116.5
4,15-Dinor-1,11(13) -xanthadiene−3,5β:12,8β-diolide	−80.7	−89.5	−78.7	−75.7	−69.9	−84.6	−67.7	−82.2	−86.4	−81.3	−90.6	−76.7
8-Epixanthatin-1β,5β-epoxide	−87.2	−90.1	−64.8	−78.4	−71.9	−103.4	−72.8	−85.4	−97.2	−90.3	−90.9	−80.6
Helenalin	−70.7	−90.8	−47.7	−76.1	−69.5	−79.3	−64.9	−80.8	−83.1	−85.2	−83.6	−79.0
Helenalin acetate	−74.2	−77.2	−76.9	−74.9	−78.5	−105.5	−74.7	−87.6	−99.3	−86.9	−78.5	−79.9
8β-Hydroxyzaluzanin D	−82.3	−85.7	−84.0	−84.3	−76.7	−101.7	−77.6	−101.1	−110.6	−82.4	−90.4	−79.3
Lactucin	−87.0	−87.4	−70.5	−72.2	−84.2	−94.7	−74.0	−92.4	−100.7	−87.8	−94.2	−79.1
Lactucopicrin	−114.3	−111.2	−65.0	−114.4	−99.5	−126.7	−108.5	−119.9	−120.4	−115.0	−116.1	−107.3
Mexicanin I	−76.3	−80.5	−74.7	−71.3	−70.4	−88.9	−63.0	−81.9	−86.6	−88.8	−92.3	−76.5
2-Oxo-8-tigloyloxyguaia-1(10),3-diene-6,12-olide-14-carboxylic acid	−97.4	−97.0	−91.1	−101.5	−81.8	−117.3	−82.4	−111.8	−118.9	−96.8	−115.4	−86.6
Peruvin	−66.7	−83.6	−64.1	−74.6	−62.7	−83.0	−61.5	−83.0	−88.5	−79.1	−83.6	−74.1
Psilostachyin	−69.6	−85.7	−6.0	−73.5	−65.8	−91.5	−63.4	−85.3	−92.7	−78.1	−88.8	−70.5
Psilostachyin C	−71.9	−81.4	−14.5	−67.6	−64.8	−85.2	−62.0	−81.6	−85.1	−77.9	−85.6	-70.9
Pungiolide A	−97.3	−102.9	−83.7	−93.9	−89.1	−110.0	−98.8	−105.0	−123.5	−111.6	−81.4	−98.4
Xanthipungolide	-50.4	−63.3	−3.8	−55.4	−57.3	−75.0	−43.1	−67.5	−66.9	−57.2	-83.7	−54.3
Zaluzanin D	−83.6	−87.2	−59.0	−79.4	−77.1	−102.6	−75.1	−96.2	−106.8	−83.6	−97.7	−86.4

**Table 9:** MolDock docking energies (kJ/mol) of guaianolide sesquiterpenoids with *Leishmania infantum* protein targets.

Guaianolides	LinfCYP51	LinfGLO2	LinfPnC1	LinfTDR1	LinfTR
Arborescin	−79.3	−64.1	−59.5	−70.7	−95.1
Carpesiolin	−81.0	−64.2	−65.0	−72.2	−82.4
Confertin	−79.8	−61.0	−41.9	−67.9	−79.8
Cynaropicrin	−106.2	−85.8	no dock	−91.6	−105.4
Damsin	−78.9	−68.5	−70.3	−68.3	−79.2
11,13-Dehydrocompressanolide	−77.9	−66.7	−68.8	−74.1	−78.2
Dehydrocostuslactone	−73.5	−61.4	−68.8	−69.1	−82.4
Dehydroleucodine	−79.6	−65.7	−72.1	−71.5	−92.8
Dehydrozaluzanin C	−81.1	−67.1	−81.8	−73.2	−85.3
Diguaiaperfolin	−141.0	−118.8	no dock	−118.6	−147.5
4,15-Dinor-1,11(13)-xanthadiene-3,5β:12,8β-diolide	−79.7	−67.8	−70.8	−76.8	−84.4
8-Epixanthatin-1β,5β-epoxide	−84.2	−77.5	−52.1	−80.4	−88.8
Helenalin	−89.0	−62.4	−24.6	−70.1	−79.7
Helenalin acetate	−85.9	−71.7	no dock	−76.1	−81.6
8β-Hydroxyzaluzanin D	−97.3	−75.2	no dock	−79.1	−93.3
Lactucin	−88.9	−72.7	no dock	−85.0	−99.8
Lactucopicrin	−114.8	−103.6	no dock	−106.7	−101.6
Mexicanin I	−79.1	−64.4	−83.3	−73.2	−79.3
2-Oxo-8-tigloyloxyguaia-1(10),3-diene-6,12-olide-14-carboxylic acid	−100.1	−94.1	no dock	−92.0	−100.8
Peruvin	−85.4	−59.4	no dock	−68.9	−75.4
Psilostachyin	−85.7	−69.7	no dock	−67.2	−84.5
Psilostachyin C	−74.1	−59.9	−55.5	−67.0	−82.1
Pungiolide A	−109.4	−97.5	−69.9	−105.6	−114.8
Xanthipungolide	−64.8	−52.0	−32.1	−47.9	−59.0
Zaluzanin D	−92.4	−75.7	no dock	−77.4	−86.5

**Table 10:** MolDock docking energies (kJ/mol) of eudesmanolide sesquiterpenoids with *Leishmania major* protein targets.

Eudesmanolides	LmajCatB	LmajDHODH	LmajdUTPase	LmajNDKb	LmajNH	LmajNMT	LmajOPB	LmajPDE1	LmajPTR1	LmajMetRS	LmajTyrRS	LmajUGPase
Alantolactone	−58.1	−90.7	−61.4	−79.0	−73.8	−72.4	−70.1	−67.2	−65.8	−83.2	−74.2	−87.9
Anthecularin	−56.1	−77.7	−62.7	−68.7	−67.2	−65.7	−73.7	−72.3	−59.6	−70.9	−72.6	−85.9
Arbusculin B	−54.8	−79.8	−67.6	−64.4	−66.2	−71.8	−75.8	−64.1	−66.6	−75.9	−73.3	−79.8
α−Cyclocostunolide	−63.7	−85.3	−75.1	−72.6	−69.7	−70.6	−74.7	−69.1	−78.5	−86.1	−77.1	−77.2
β−Cyclocostunolide	−64.6	−85.9	−67.0	−73.5	−67.9	−69.7	−76.1	−66.7	−81.9	−83.2	−73.0	−77.9
Deacetyl−β−cyclopryethrosin	−69.1	−97.8	−73.4	−76.4	−72.9	−80.2	−82.3	−76.9	−83.3	−93.6	−76.0	−91.1
11,13−Dihydrovernodalin	−78.0	−110.6	−91.9	−100.3	−89.7	−99.7	−99.0	−106.2	−103.4	−114.7	−104.0	−94.5
Douglanin	−66.7	−90.4	−76.9	−71.6	−71.9	−69.6	−77.8	−74.7	−79.4	−88.2	−77.1	−78.5
Frullanolide	−60.6	−81.1	−71.3	−73.2	−69.2	−73.1	−76.9	−77.3	−73.5	−80.4	−75.1	−82.6
4−Hydroxyanthecotulide	−76.1	−104.1	−90.1	−103.0	−88.3	−101.7	−95.0	−100.5	−100.8	−103.2	−98.1	−112.3
8β−[4−Hydroxy−5−(5−hydroxytigloyloxy)-tigloyl]santamarin	−109.5	−128.9	−116.3	−125.3	−117.8	−115.9	−113.3	−117.5	−130.2	−153.1	−128.3	−127.1
Isoalantolactone	−60.2	−89.6	−65.0	−82.0	−74.4	−74.4	−70.3	−72.2	−66.4	−87.3	−74.2	−88.6
Ivalin	−66.1	−94.6	−68.6	−78.4	−74.3	−80.7	−74.8	−74.6	−73.3	−88.8	−73.7	−92.0
Ivalin acetate	−77.4	−93.6	−87.4	−87.4	−83.2	−89.5	−90.8	−89.6	−91.6	−104.5	−87.8	−83.4
Onoseriolide	−72.4	−92.4	−82.8	−81.4	−78.5	−79.8	−88.5	−83.8	−88.6	−101.4	−81.3	−82.5
2−Oxoalantolactone	−59.1	−94.0	−64.8	−79.6	−76.3	−78.0	−75.0	−69.7	−68.4	−85.5	−75.0	−91.1
Oxyonoseriolide	−77.5	−94.5	−82.3	−80.4	−81.5	−88.9	−88.7	−88.0	−87.8	−104.7	−92.7	−99.1
4−Peroxy−1,2,4,5−tetrahydro−α−santonin	−63.3	−97.6	−72.6	−71.0	−72.5	−74.1	−87.3	−80.3	−90.1	−85.2	−76.6	−82.4
Santamarin	−65.3	−86.0	−74.9	−72.7	−72.6	−71.6	−80.5	−73.1	−79.0	−89.3	−79.4	−79.4
α−Santonin	−63.8	−92.0	−73.1	−73.4	−68.6	−71.3	−84.1	−78.0	−82.7	−85.6	−85.1	−84.1
Sivasinolide	−69.8	−90.0	−68.9	−84.5	−72.9	−78.7	−84.5	−72.6	−86.8	−98.1	−80.6	−82.9
Trilobolide 6−isobutyrate	−86.0	−108.8	−90.3	−93.8	−95.6	−103.7	−102.2	−97.6	−82.6	−87.4	−95.1	−94.7
Trilobolide 6−methacrylate	−78.4	−103.8	−87.9	−85.0	−94.2	−111.3	−87.6	−97.6	−78.9	−87.5	−92.1	−92.2
Vernangulide B	−91.2	−118.3	−108.7	−105.8	−105.6	−113.0	−99.8	−107.1	−122.9	−125.3	−105.0	−111.9
Vernodalin	−80.8	−107.5	−94.4	−101.0	−92.4	−105.7	−89.4	−92.4	−104.5	−116.3	−95.8	−102.2
Vernodalol	−82.5	−112.8	−100.1	−99.1	−96.4	−97.3	−94.5	−99.4	−100.0	−107.8	−94.6	−90.2
Wedelolide A	−88.3	−109.9	−95.0	−83.5	−94.0	−109.6	−84.2	−103.7	−83.1	−131.6	−96.2	−98.4
Wedelolide B	−88.0	−113.0	−91.2	−87.0	−94.2	−107.4	−99.0	−107.4	−82.8	−134.0	−102.8	−94.9

**Table 11:** MolDock docking energies (kJ/mol) of eudesmanolide sesquiterpenoids with *Leishmania donovani* and *L. mexicana* protein targets.

Eudesmanolides	LdonCatB	LdonCyp	LdonDHODH	LdonNMT	LmexGAPDH	LmexGPDH	LmexPGI	LmexPMM	LmexPYK	LmexPYK	LmexPYK	LmexTIM
									Site 1	Site 2	Site 3	
Alantolactone	−67.1	−78.3	−55.0	−64.4	−62.6	−79.8	−57.8	−74.6	−78.6	−72.2	−79.7	−77.9
Anthecularin	−62.4	−65.2	−57.0	−63.9	−60.1	−80.3	−59.8	−70.9	−79.7	−64.4	−81.3	−69.7
Arbusculin B	−60.0	−74.6	−58.2	−63.5	−64.3	−84.1	−55.6	−75.3	−73.9	−69.6	−74.8	−70.3
α−Cyclocostunolide	−67.2	−78.6	−62.6	−62.9	−60.5	−83.6	−57.3	−73.7	−78.8	−73.8	−80.1	−68.0
β−Cyclocostunolide	−69.9	−78.0	−69.9	−67.0	−60.2	−85.0	−58.9	−78.3	−76.3	−75.2	−87.9	−73.4
Deacetyl−β−cyclopryethrosin	−73.4	−82.9	−63.3	−72.8	−71.5	−92.2	−63.3	−84.0	−84.6	−78.1	−88.1	−81.4
11,13−Dihydrovernodalin	−89.3	−94.8	−59.5	−84.3	−79.3	−107.0	−82.3	−102.5	−104.4	−94.9	−100.7	−87.1
Douglanin	−69.0	−80.6	−71.4	−66.3	−63.7	−88.6	−58.9	−76.6	−82.7	−77.2	−82.1	−65.1
Frullanolide	−61.5	−77.8	−65.2	−63.1	−67.0	−79.4	−58.7	−75.6	−82.1	−72.5	−86.1	−72.8
4−Hydroxyanthecotulide	−87.9	−94.0	−93.9	−86.1	−86.4	−103.7	−85.0	−100.3	−98.8	−91.5	−98.0	−97.0
8β−[4−Hydroxy−5−(5−hydroxytigloyloxy)-tigloyl]santamarin	−112.4	−115.3	−37.4	−121.4	−96.5	−128.2	−113.8	−132.1	−124.3	−119.9	−112.8	−105.4
Isoalantolactone	−69.9	−83.1	−70.6	−64.4	−59.3	−82.4	−56.8	−77.4	−80.0	−69.7	−85.1	−74.0
Ivalin	−77.3	−84.5	−74.6	−73.2	−62.5	−84.1	−62.0	−80.9	−83.6	−73.4	−88.9	−70.0
Ivalin acetate	−91.5	−92.5	−63.2	−66.9	−73.8	−97.0	−70.5	−92.4	−97.2	−80.3	−81.4	−86.8
Onoseriolide	−73.8	−88.1	−84.8	−77.0	−72.8	−93.9	−66.8	−84.1	−87.9	−78.0	−95.0	−77.1
2−Oxoalantolactone	−64.0	−80.8	−58.1	−64.1	−65.3	−85.5	−61.4	−77.8	−81.7	−74.7	−83.5	−77.9
Oxyonoseriolide	−77.9	−84.5	−61.8	−78.5	−78.5	−95.4	−70.4	−84.6	−98.4	−80.6	−103.9	−79.3
4−Peroxy−1,2,4,5−tetrahydro−α−santonin	−68.0	−72.4	−68.6	−72.3	−62.0	−92.4	−58.1	−82.3	−85.2	−73.3	−83.0	−73.0
Santamarin	−69.3	−79.8	−56.3	−67.4	−65.6	−89.5	−61.2	−81.2	−82.6	−75.9	−82.0	−71.6
α−Santonin	−68.9	−78.3	−71.0	−68.6	−65.5	−85.6	−57.8	−82.5	−80.7	−69.1	−85.4	−72.0
Sivasinolide	−71.6	−85.1	−61.8	−73.5	−68.1	−94.9	−62.3	−84.7	−88.4	−77.9	−91.5	−76.4
Trilobolide 6−isobutyrate	−82.4	−83.4	−71.3	−80.7	−79.8	−99.2	−84.5	−103.2	−96.5	−89.4	−92.1	−72.4
Trilobolide 6−methacrylate	−83.4	−80.8	−20.7	−82.7	−70.3	−99.2	−88.3	−107.3	−94.9	−92.7	−87.2	−74.7
Vernangulide B	−93.8	−106.6	−126.8	−96.1	−90.5	−121.5	−100.6	−120.1	−116.6	−105.6	−107.5	−104.7
Vernodalin	−84.6	−94.2	−79.2	−87.4	−80.6	−110.8	−81.5	−106.2	−104.5	−106.1	−99.5	−94.0
Vernodalol	−88.4	−96.3	−50.6	−83.9	−84.1	−105.2	−93.5	−99.8	−103.5	−99.1	−103.4	−83.2
Wedelolide A	−87.9	−68.5	−85.5	−91.6	−84.0	−96.0	−90.3	−114.9	−103.2	−107.4	−92.9	−92.7
Wedelolide B	−81.5	−64.3	−88.3	−97.5	−79.2	−96.0	−92.2	−127.4	−102.4	−108.8	−102.6	−96.0

**Table 12:** MolDock docking energies (kJ/mol) of eudesmanolide sesquiterpenoids with *Leishmania infantum* protein targets.

Eudesmanolides	LinfCYP51	LinfGLO2	LinfPnC1	LinfTDR1	LinfTR
Alantolactone	−71.3	−60.0	no dock	−66.1	−75.5
Anthecularin	−70.8	−47.5	−58.4	−62.3	−71.0
Arbusculin B	−71.0	−58.8	−12.7	−62.1	−73.6
α−Cyclocostunolide	−70.4	−60.3	−82.7	−69.1	−79.0
β−Cyclocostunolide	−75.9	−62.0	−79.0	−71.6	−73.1
γ−Cyclocostunolide	−71.0	−57.5	−12.1	−62.2	−67.4
Deacetyl−β−cyclopryethrosin	−74.8	−64.8	−75.5	−71.5	−82.2
11,13−Dihydrovernodalin	−105.4	−83.0	no dock	−85.8	−95.4
Douglanin	−76.4	−60.7	−10.6	−67.9	−80.1
Frullanolide	−75.2	−61.3	−55.4	−70.5	−74.5
4−Hydroxyanthecotulide	−90.2	−82.6	−68.4	−85.4	−92.1
8β−[4−Hydroxy−5−(5−hydroxytigloyloxy)-tigloyl]santamarin	−117.8	−98.9	no dock	−103.4	−114.9
Isoalantolactone	−69.1	−57.8	−31.1	−64.3	−74.2
Ivalin	−72.9	−65.0	−16.1	−65.6	−80.5
Ivalin acetate	−88.7	−72.0	no dock	−76.6	−91.2
Onoseriolide	−82.0	−70.0	−24.6	−71.8	−82.4
2−Oxoalantolactone	−73.4	−57.6	no dock	−68.1	−79.4
Oxyonoseriolide	−86.7	−71.3	−11.8	−78.4	−87.9
4−Peroxy−1,2,4,5−tetrahydro−α−santonin	−80.6	−73.2	−66.0	−66.5	−78.2
Santamarin	−67.8	−59.5	−85.2	−71.3	−81.3
α−Santonin	−74.2	−69.4	−44.0	−65.5	−86.4
Sivasinolide	−75.2	−63.3	−72.1	−72.5	−81.1
Trilobide 6−isobutyrate	−97.9	−82.0	no dock	−80.3	−90.2
Trilobide 6−methacrylate	−96.0	−86.3	no dock	−77.2	−90.2
Vernangulide B	−105.2	−98.6	no dock	−99.3	−99.0
Vernodalin	−105.9	−88.9	no dock	−86.6	−96.9
Vernodalol	−103.1	−79.4	no dock	−88.9	−96.8
Wedelolide A	−97.6	−80.8	no dock	−90.4	−99.4
Wedelolide B	−105.8	−77.6	no dock	−92.8	−96.6

**Table 13:** MolDock docking energies (kJ/mol) of miscellaneous sesquiterpenoids with *Leishmania major* protein targets.

Miscellaneous Sesquiterpenoids	LmajCatB	LmajDHODH	LmajdUTPase	LmajNDKb	LmajNH	LmajNMT	LmajOPB	LmajPDE1	LmajPTR1	LmajMetRS	LmajTyrRS	LmajUGPase
Alloaromadendrene	−56.8	−81.7	−67.0	−68.2	−66.1	−71.1	−74.3	−68.2	−91.9	−70.7	−70.0	−76.6
Aromadendrene	−56.9	−78.5	−68.4	−66.9	−64.7	−70.2	−73.2	−67.9	−99.4	−73.2	−72.4	−72.2
1,10−Bisaboladiene−3,4−diol	−67.4	−92.7	−81.0	−82.8	−79.8	−87.5	−89.2	−82.9	−102.6	−91.1	−80.7	−89.5
α−Bisabolol	−75.2	−92.5	−76.3	−77.5	−75.6	−89.9	−74.7	−79.9	−110.6	−93.8	−80.3	−83.2
Corymbolone	−56.5	−82.2	−59.1	−67.5	−70.2	−66.5	−69.5	−65.5	−59.5	−77.2	−68.3	−85.9
α−Eudesmol	−60.9	−80.3	−64.8	−70.7	−68.1	−66.0	−66.0	−74.2	−86.8	−83.1	−76.9	−74.7
β−Eudesmol	−58.8	−83.4	−69.6	−69.6	−66.5	−68.2	−72.7	−67.3	−89.6	−76.9	−68.1	−79.1
1(10),5−Germacradien−4−ol	−62.0	−88.1	−68.8	−69.1	−73.3	−70.1	−76.0	−72.2	−100.0	−87.1	−75.0	−88.6
Germacrene D	−57.9	−81.4	−66.6	−65.1	−70.8	−68.8	−69.7	−69.9	−96.7	−77.7	−70.6	−81.8
Gossypol	−59.1	−106.1	−85.2	−89.5	−83.9	−104.6	−98.4	−111.5	−120.6	−92.7	−108.0	−90.7
Gossypol−6,6*'*−dimethylether	−90.8	−108.3	−84.1	−95.0	−85.6	−95.5	−84.6	−114.3	−117.5	−88.0	−108.6	−100.3
Gossypol−6−methylether	−93.1	−109.1	−85.9	−93.7	−103.2	−116.8	−102.1	−113.3	−122.2	−94.1	−111.6	−95.6
Homalomenol C	−67.8	−88.7	−65.1	−69.4	−72.6	−72.4	−76.6	−74.7	−92.6	−83.9	−75.5	−88.6
1−Hydroperoxy−10(14),11−guaiadiene	−60.7	−85.4	−70.4	−70.7	−69.0	−74.0	−77.5	−78.5	−96.5	−83.6	−76.5	−83.7
10−Hydroperoxy−1,11−guaiadiene	−64.6	−82.2	−76.5	−73.3	−74.6	−77.5	−77.5	−82.7	−109.1	−86.4	−80.1	−79.9
14−Hydroperoxy−1(10),11−guaiadiene	−71.5	−88.0	−88.1	−79.2	−78.4	−78.5	−80.0	−82.3	−111.6	−87.9	−79.1	−86.6
Kudtriol	−60.6	−86.7	−65.3	−68.9	−72.5	−71.5	−66.2	−69.2	−81.5	−82.3	−68.0	−80.5
5−*epi*−Kudtriol	−59.9	−86.5	−69.2	−68.4	−70.4	−78.2	−75.8	−75.8	−65.9	−77.9	−65.1	−83.0
Longifolene	−52.1	−75.8	−50.0	−62.3	−59.6	−58.9	−63.0	−66.7	−61.3	−62.3	−63.1	−70.8
Mukaadial	−67.0	−89.5	−71.1	−72.7	−67.3	−75.0	−76.6	−72.0	−80.7	−79.2	−82.3	−81.8
Mustakone	−47.5	−78.0	−61.9	−62.0	−64.5	−64.2	−67.6	−67.5	−66.1	−71.7	−65.4	−81.7
Muzigadial	−64.6	−93.9	−66.8	−70.6	−65.0	−70.6	−74.1	−66.3	−81.5	−81.9	−68.5	−76.8
Nerolidol	−69.5	−91.4	−86.4	−86.5	−81.2	−93.0	−84.3	−89.8	−117.0	−100.5	−87.9	−94.2
Oplopanone	−59.4	−81.9	−69.4	−66.1	−68.2	−70.8	−73.0	−71.7	−106.4	−76.9	−74.0	−74.2
10,12−Peroxycalamenene	−41.0	−71.0	−57.9	−57.4	−60.8	−65.9	−66.3	−74.3	−70.6	−69.3	−68.4	−70.7
Plagiochilin A	−83.4	−103.9	−94.1	−94.1	−93.6	−88.5	−95.7	−92.7	−132.3	−102.9	−106.0	−105.9
Polygodial	−61.3	−86.0	−70.7	−71.6	−64.8	−71.0	−76.9	−66.6	−91.1	−86.3	−71.5	−80.4
Zingiberene−3,6−α−peroxide	−62.3	−89.4	−75.8	−75.4	−76.0	−82.6	−68.3	−84.9	−87.4	−92.0	−76.8	−88.2
Zingiberene−3,6−β−peroxide	−62.2	−86.9	−73.6	−75.4	−74.9	−78.7	−72.4	−84.7	−89.9	−82.0	−73.7	−84.9

**Table 14:** MolDock docking energies (kJ/mol) of miscellaneous sesquiterpenoids with *Leishmania donovani* and *L. mexicana* protein targets.

Miscellaneous Sesquiterpenoids	LdonCatB	LdonCyp	LdonDHODH	LdonNMT	LmexGAPDH	LmexGPDH	LmexPGI	LmexPMM	LmexPYK	LmexPYK	LmexPYK	LmexTIM
									Site 1	Site 2	Site 3	
Alloaromadendrene	−66.6	−77.8	−46.3	−58.8	−61.3	−78.3	−57.1	−71.4	−79.0	−62.3	−79.7	−68.9
Aromadendrene	−66.1	−72.0	−56.6	−62.4	−57.1	−75.0	−54.4	−71.9	−79.4	−65.5	−77.1	−65.1
1,10−Bisaboladiene−3,4−diol	−73.0	−82.3	−74.6	−85.5	−70.9	−94.2	−71.9	−78.6	−88.2	−76.9	−84.5	−80.0
α−Bisabolol	−75.2	−76.8	−68.1	−81.0	−65.2	−85.2	−67.0	−84.8	−90.3	−75.7	−79.7	−77.7
Corymbolone	−60.9	−72.2	−62.0	−63.5	−60.6	−74.4	−56.1	−70.5	−74.6	−72.4	−80.2	−69.4
6α,9α−Dihydroxypolygodial	−65.8	−86.7	−61.9	−72.3	−65.5	−88.4	−58.7	−77.1	−83.6	−74.4	−91.4	−69.6
α−Eudesmol	−62.3	−74.4	−49.2	−57.4	−57.2	−78.4	−53.9	−76.3	−73.5	−64.3	−82.9	−67.2
β−Eudesmol	−66.1	−74.1	−57.0	−63.4	−58.8	−75.1	−56.5	−77.8	−72.4	−65.5	−83.0	−68.3
1(10),5−Germacradien−4−ol	−69.7	−78.5	−49.2	−70.5	−62.2	−81.5	−58.6	−75.2	−78.1	−68.4	−84.1	−74.8
Germacrene D	−65.0	−74.6	−64.4	−68.3	−60.1	−77.0	−58.3	−70.1	−73.5	−64.9	−82.0	−71.3
Gossypol	−79.2	−86.4	−88.4	−64.5	−81.7	−117.4	−76.4	−116.4	−110.5	−96.9	−93.4	−86.4
Gossypol−6,6*'*−dimethylether	−89.6	−82.6	−90.2	−82.3	−71.9	−112.2	−81.8	−119.8	−113.9	−103.5	−96.5	−84.3
Gossypol−6−methylether	−82.2	−90.0	−87.5	−82.4	−69.4	−113.0	−75.3	−115.2	−111.7	−103.6	−101.0	−83.4
Homalomenol C	−66.5	−75.6	−26.8	−65.5	−64.4	−89.4	−63.4	−74.5	−87.0	−68.4	−84.5	−73.1
1−Hydroperoxy−10(14),11−guaiadiene	−59.3	−78.1	−59.8	−61.2	−63.7	−85.3	−61.7	−73.4	−81.5	−74.5	−79.2	−68.5
10−Hydroperoxy−1,11−guaiadiene	−77.0	−82.0	−67.9	−66.3	−66.3	−83.7	−60.1	−80.2	−86.0	−73.5	−85.7	−78.9
14−Hydroperoxy−1(10),11−guaiadiene	−78.4	−92.8	−85.2	−69.2	−70.4	−93.9	−67.8	−79.5	−87.7	−75.2	−91.0	−79.6
Kudtriol	−69.5	−78.5	−66.2	−65.6	−61.3	−79.2	−58.9	−73.8	−78.6	−69.7	−93.0	−70.4
5−*epi*−Kudtriol	−59.0	−78.2	−65.4	−66.2	−69.3	−79.6	−55.7	−76.8	−77.6	−70.7	−83.7	−68.5
Longifolene	−52.4	−69.7	−35.7	−56.3	−57.6	−74.0	−52.4	−63.5	−68.1	−63.1	−66.8	−60.8
Mukaadial	−65.9	−86.9	−61.9	−72.3	−72.0	−92.6	−58.7	−77.1	−83.7	−74.5	−91.5	−69.6
Mustakone	−57.7	−57.0	−61.7	−59.3	−56.1	−76.2	−53.7	−69.0	−72.7	−63.2	−73.7	−63.2
Muzigadial	−70.3	−85.5	−15.2	−62.1	−66.3	−78.6	−57.9	−76.5	−80.4	−73.1	−81.2	−71.5
Nerolidol	−79.6	−82.0	−87.3	−76.3	−74.5	−91.1	−79.5	−84.3	−87.3	−76.5	−91.7	−85.8
Oplopanone	−68.5	−78.9	−64.1	−65.2	−59.8	−78.2	−58.4	−71.5	−77.0	−67.2	−78.5	−68.5
10,12−Peroxycalamenene	−51.6	−70.8	−62.9	−54.6	−58.3	−71.8	−49.9	−71.3	−66.5	−61.4	−69.9	−62.4
Plagiochilin A	−96.2	−89.1	−95.1	−81.9	−78.8	−105.9	−75.5	−99.4	−99.8	−97.9	−87.5	−89.2
Polygodial	−60.0	−80.5	−57.6	−68.6	−62.1	−81.9	−55.2	−73.3	−78.4	−69.2	−84.7	−76.3
Zingiberene−3,6−α−peroxide	−75.1	−71.7	−80.1	−74.3	−67.0	−83.7	−67.1	−80.7	−79.9	−74.0	−81.1	−76.1
Zingiberene−3,6−β−peroxide	−71.6	−75.1	−77.1	−76.2	−60.7	−84.3	−66.0	−74.9	−83.0	−67.0	−87.3	−74.6

**Table 15:** MolDock docking energies (kJ/mol) of miscellaneous sesquiterpenoids with *Leishmania infantum* protein targets.

Miscellaneous Sesquiterpenoids	LinfCYP51	LinfGLO2	LinfPnC1	LinfTDR1	LinfTR
Alloaromadendrene	−66.8	−57.0	−52.9	−61.7	−67.2
Aromadendrene	−60.8	−59.7	−60.8	−61.6	−76.8
1,10−Bisaboladiene−3,4−diol	−75.6	−70.8	−63.5	−72.1	−85.0
α−Bisabolol	−76.5	−74.1	−73.4	−75.0	−74.7
Corymbolone	−65.9	−54.6	−49.1	−59.4	−65.3
6α,9α−Dihydroxypolygodial	−75.7	−62.3	−23.0	−63.6	−78.0
α−Eudesmol	−62.6	−58.1	−28.2	−59.1	−69.5
β−Eudesmol	−68.0	−59.6	−23.9	−64.3	−69.7
1(10),5−Germacradien−4−ol	−70.4	−58.4	−52.6	−63.6	−70.2
Germacrene D	−64.6	−57.4	−62.7	−68.0	−70.2
Gossypol	−109.9	−99.6	no dock	−86.6	−97.0
Gossypol−6,6*'*−dimethylether	−109.5	−95.1	no dock	−88.7	−101.1
Gossypol−6−methylether	−113.1	−99.6	no dock	−94.5	−100.9
Homalomenol C	−72.3	−57.3	−41.9	−64.4	−68.5
1−Hydroperoxy−10(14),11−guaiadiene	−74.5	−56.7	−15.4	−62.9	−72.8
10−Hydroperoxy−1,11−guaiadiene	−79.3	−66.5	−38.7	−67.3	−75.4
14−Hydroperoxy−1(10),11−guaiadiene	−81.4	−67.2	−69.9	−74.2	−74.4
Kudtriol	−68.1	−51.5	no dock	−65.3	−72.4
5−*epi*−Kudtriol	−67.9	−61.6	no dock	−63.0	−71.5
Longifolene	−64.9	−49.4	−48.0	−54.6	−61.5
Mukaadial	−74.9	−61.5	−21.0	−63.7	−78.0
Mustakone	−63.5	−57.5	−18.1	−55.3	−63.3
Muzigadial	−72.6	−56.5	−66.6	−65.1	−84.6
Nerolidol	−79.3	−72.3	−72.0	−79.1	−82.5
Oplopanone	−65.7	−59.4	−55.6	−63.2	−69.2
10,12−Peroxycalamenene	−65.2	−46.6	no dock	−58.2	−70.5
Plagiochilin A	−95.9	−79.6	no dock	−80.5	−89.3
Polygodial	−72.1	−57.6	−45.3	−62.8	−72.3
Zingiberene−3,6−α−peroxide	−77.7	−65.4	−65.8	−66.7	−75.1
Zingiberene−3,6−β−peroxide	−69.8	−65.5	−51.2	−62.7	−72.9

The guaianolide with the strongest docking energy was diguaiaperfolin, probably owing to its dimeric structure and larger molecular weight (716.77 amu). This ligand did show notable docking (−154.5 kJ/mol) with LmajDHODH as well as with LmajUGPase (docking energy = −151.8 kJ/mol). 8β-[4-Hydroxy-5-(5-hydroxytigloyloxy)tigloyl]santamarin was the strongest-docking eudesmanolide, and this ligand showed docking selectivity to LmajMetRS (docking energy = −153.1 kJ/mol). The proteins most strongly targeted by both the guaianolides and the eudesmaolides were also LmajMetRS and LmajDHODH. Interestingly, the miscellaneous sesquiterpenoids preferentially targeted *L. major* pteridine reductase 1 (LmajPTR1), and plagiochilin A showed notable selectivity (docking energy = −132.2 kJ/mol) for this protein.

**Figure 6:**
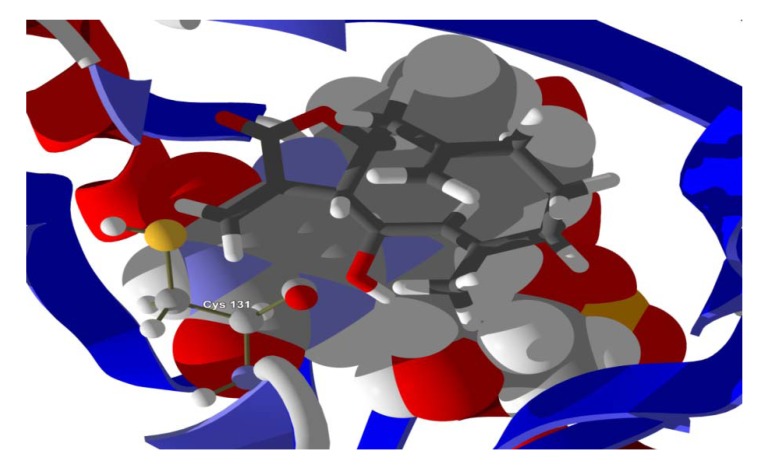
Lowest-energy docked pose of tatridin A with *L. major* dihydroorotate dehydrogenase (LmajDHODH, PDB 3mhu). The cofactor, riboflavin monophosphate, is shown as a space-filling structure.

**Figure 7:**
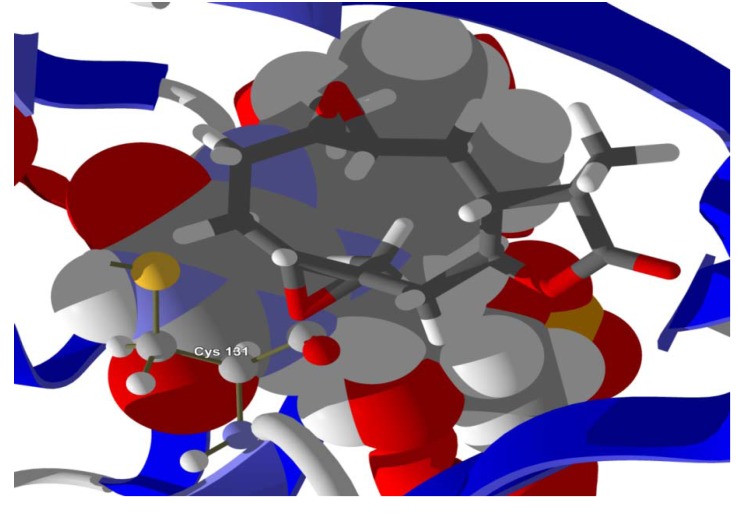
Lowest-energy docked pose of 11-epi-ivaxillin with *L. major* dihydroorotate dehydrogenase (LmajDHODH, PDB 3mhu). The cofactor, riboflavin monophosphate, is shown as a space-filling structure.

**Figure 8:**
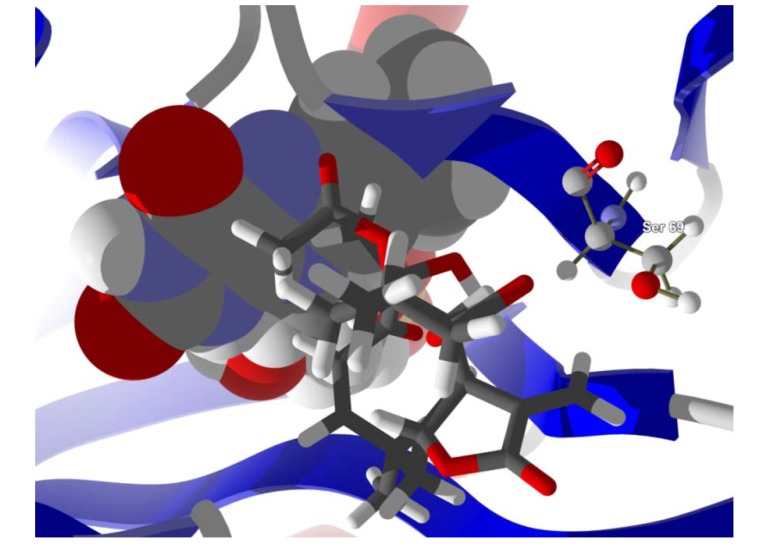
Lowest-energy docked pose of neurolenin B with *L. major* dihydroorotate dehydrogenase (LmajDHODH, PDB 3mhu). The cofactor, riboflavin monophosphate, is shown as a space-filling structure.

**Figure 9:**
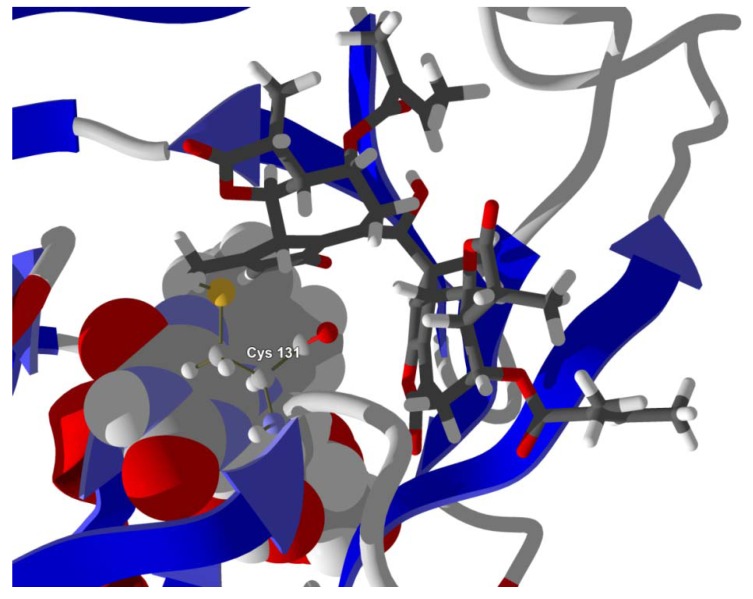
Lowest-energy docked pose of diguaiaperfolin with *L. major* dihydroorotate dehydrogenase (LmajDHODH, PDB 3mhu). The cofactor, riboflavin monophosphate, is shown as a space-filling structure.

Several electrophilic sesquiterpenoids have exhibited antiprotozoal activity [5] and many of these showed docking selectivity to LmajDHODH. The active site of this protein has some potential nucleophilic residues, namely Ser 69, Ser 196, and Cys 131. Suitably oriented electrophilic ligands could form covalent bonds with these nucleophiles and thus inhibit the enzyme. Thus, for example, the germacranolide tatridin A docked preferentially to LmajDHODH, and the lowest-energy docked pose oriented the electrophilic carbon of the α-methylene lactone moiety close to the sulfur atom of Cys 131 (see Figure 6). Similarly, the lowest-energy docked orientation of 11-*epi*-ivaxillin places one of the epoxide groups near to the sulfur atom of Cys 131 (Figure 7). Conversely, the lowest-energy docked pose of neurolenin B is such that the electrophilic carbon of the α-methylene lactone group of the ligand is near the hydroxyl group of Ser 69 (Figure 8). The ligand with the lowest docking energy to LmajDHODH was the guaianolide dimer diguaiaperfolin (−154.5 kJ/mol). The lowest-energy pose for this ligand placed the cyclopentenone moiety near the sulfur atom of Cys 131 (Figure 9).

### 2.3. Diterpenoid Docking

**Figure 10:**
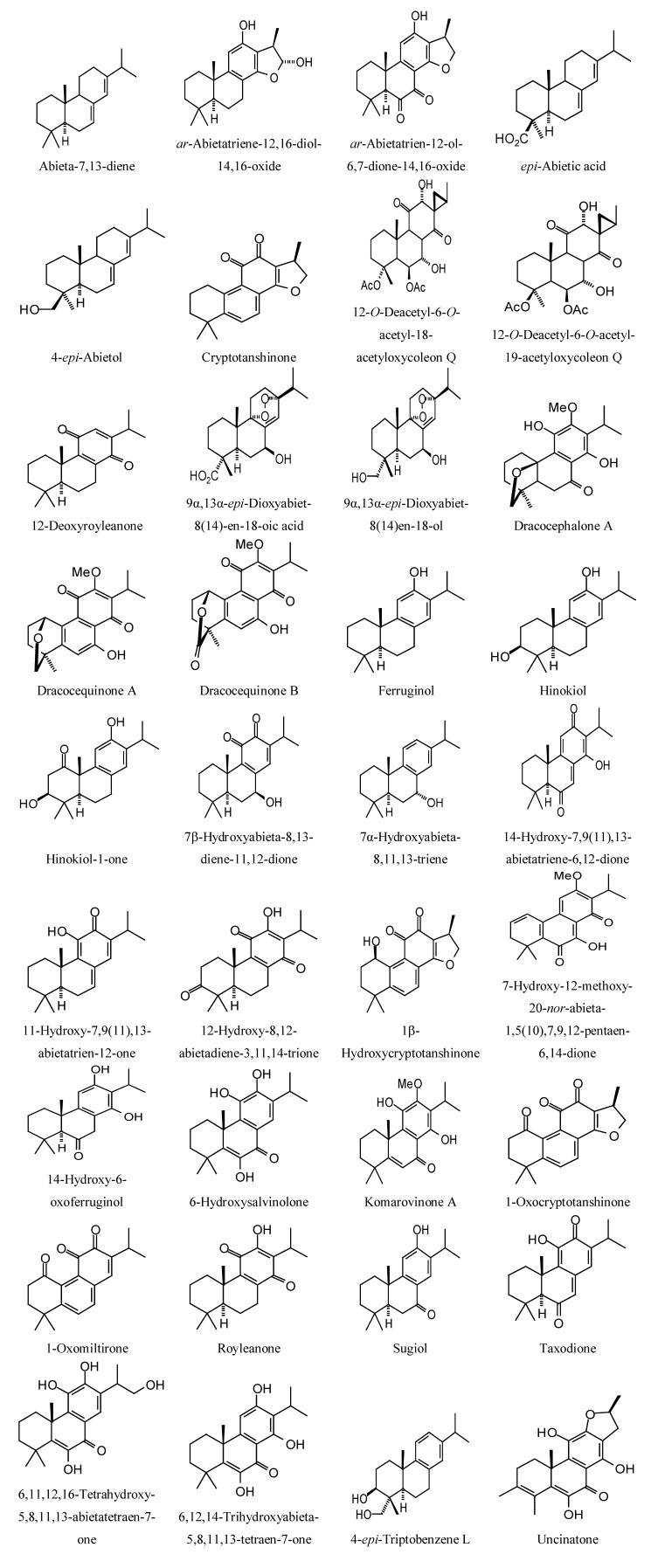
Abietane diterpenoids examined in this study.

**Figure 11:**
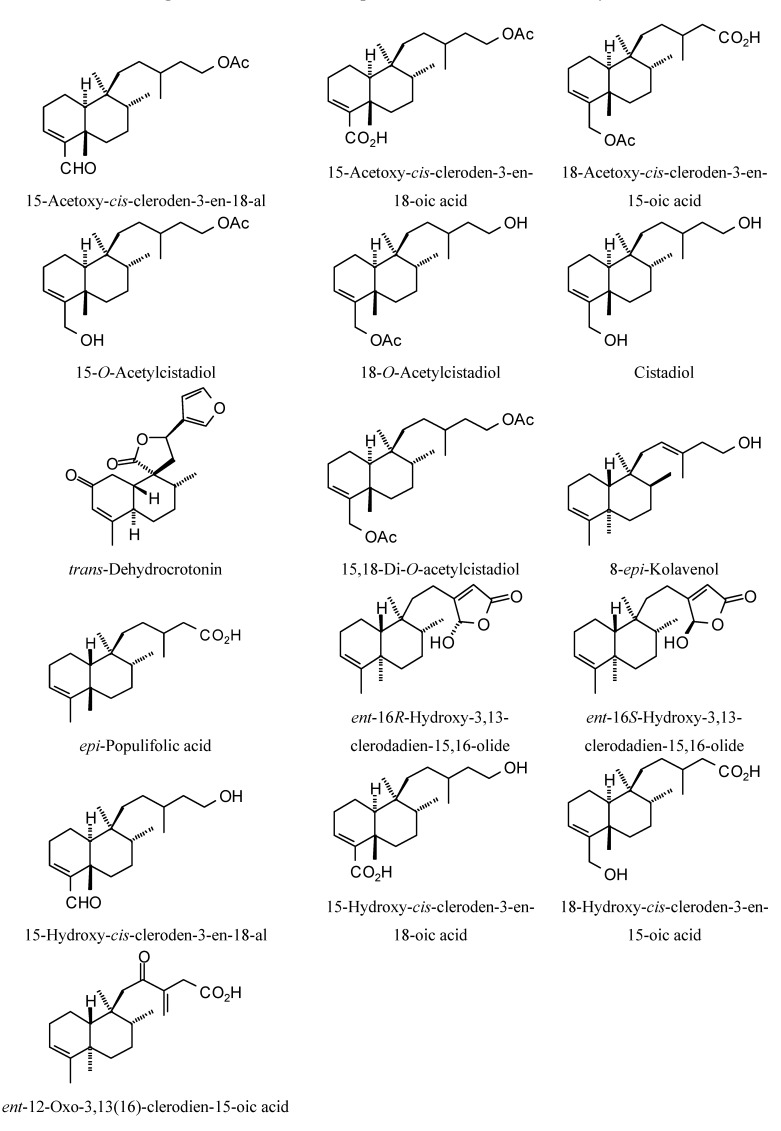
Clerodane diterpenoids examined in this study.

**Figure 12:**
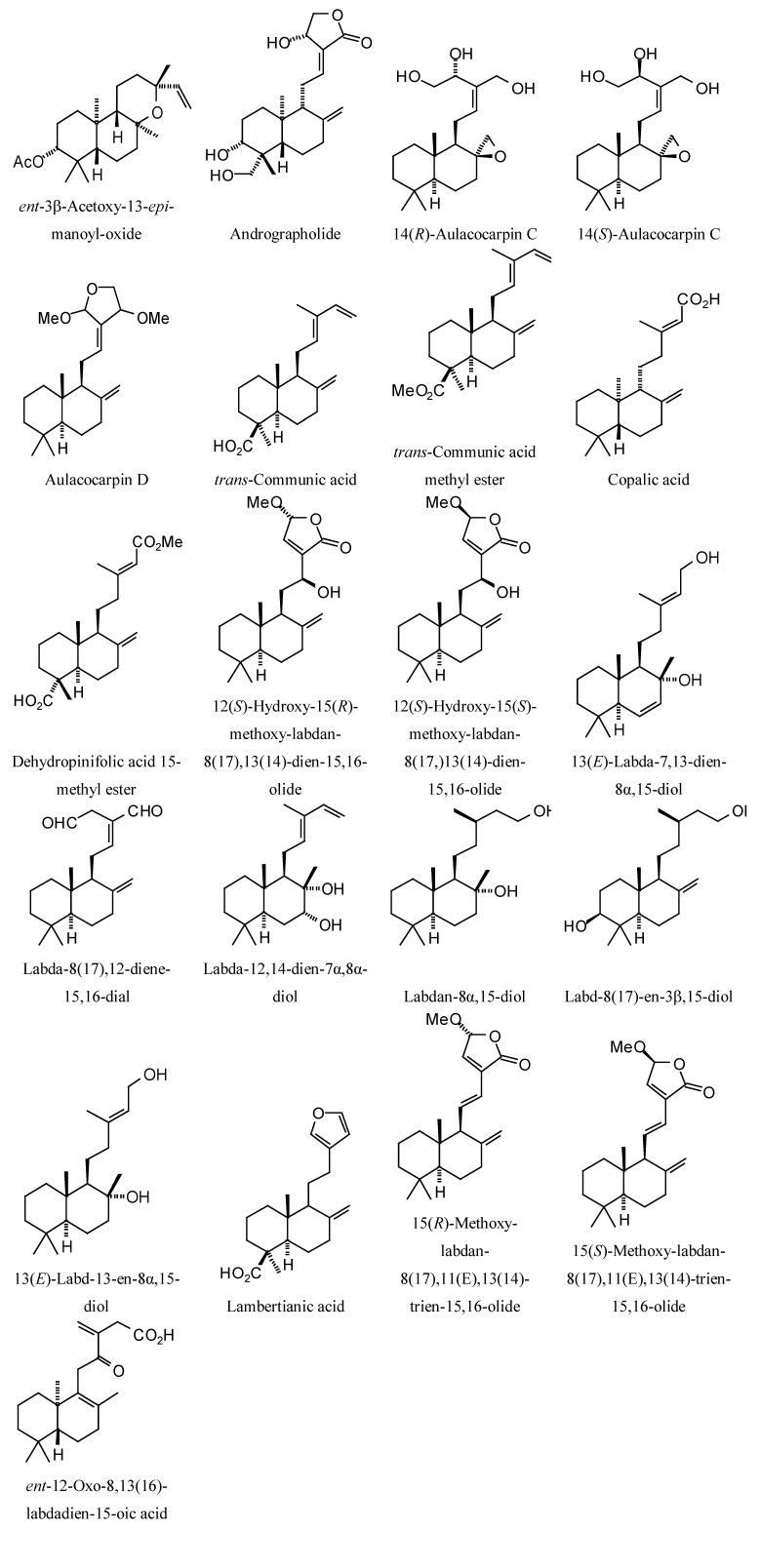
Labdane diterpenoids examined in this study.

**Figure 13:**
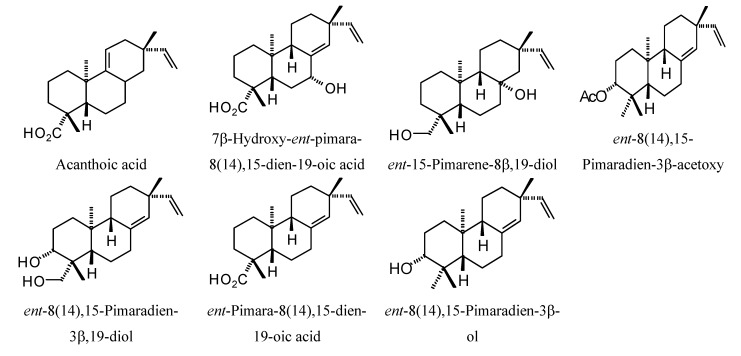
Kaurane diterpenoids examined in this study.

**Figure 14:**
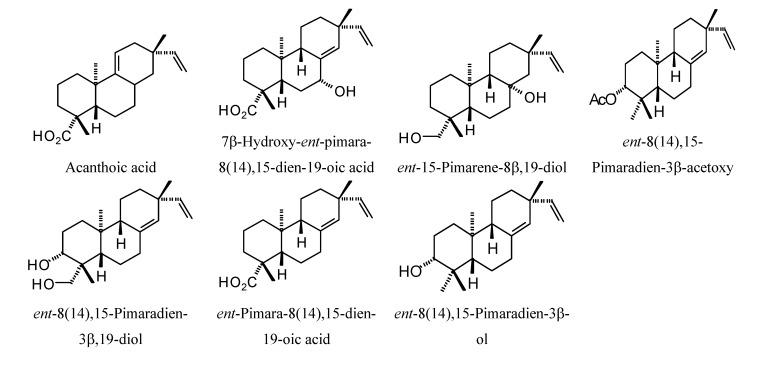
Pimarane diterpenoids examined in this study.

**Figure 15:**
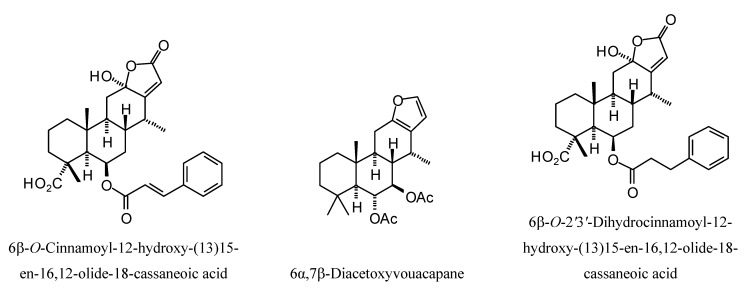
Cassane diterpenoids examined in this study.

**Figure 16:**
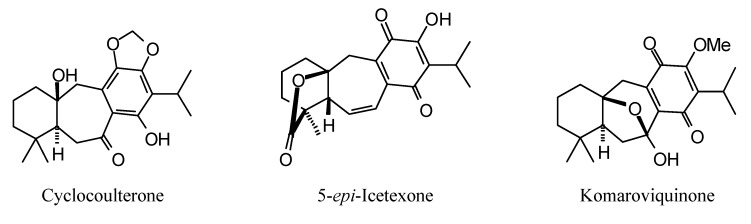
Icetaxane diterpenoids examined in this study.

**Figure 17:**
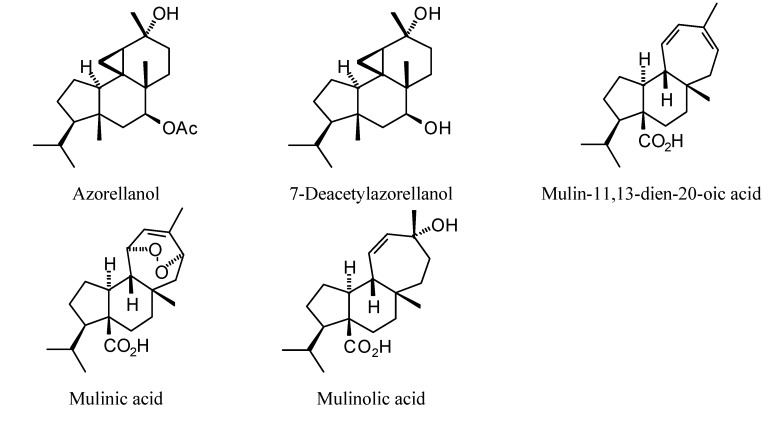
Mulinane diterpenoids examined in this study.

**Figure 18:**
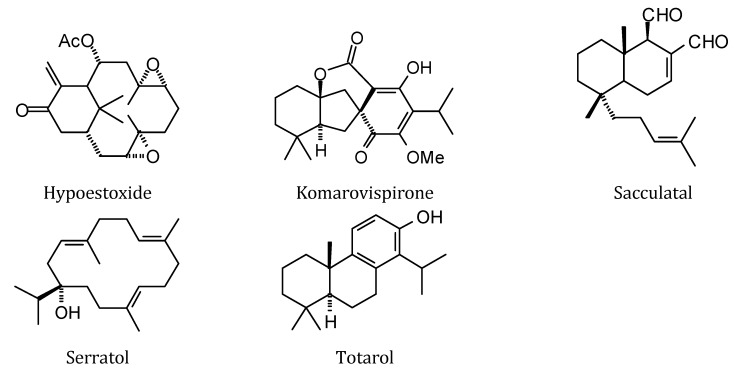
Miscellaneoous diterpenoids examined in this study.

Structures of diterpenoids are shown in Figure 10, Figure 11, Figure 12, Figure 13, Figure 14, Figure 15, Figure 16, Figure 17 and Figure 18. Docking energies of the diterpenoids are assembled in Table 16, Table 17, Table 18, Table 19, Table 20, Table 21, Table 22, Table 23, Table 24, Table 25, Table 26, Table 27 and Table 28. The diterpenoids ligands generally favored docking to *L. mexicana* glycerol-3-phosphate dehydrogenase (LmexGPDH). In particular, the kaurane diterpenoids strongly docked to this target. In addition to LmexGPDH, labdane diterpenoids showed docking preferences for LmajMetRS and LmajDHODH. The strongest-docking ligands were the cinnamoyl cassanes 6β-*O*-cinnamoyl-12-hydroxy-(13)15-en-16,12-olide-18-cassaneoic acid and 6β-O-2*'*,3*'*-dihydro-cinnamoyl-12-hydroxy-(13)15-en-16,12-olide-18-cassaneoic acid. These two ligands showed significant docking preference to LmajMetRS and LmexPMM.

**Table 16:** MolDock docking energies (kJ/mol) of abietane diterpenoids with *Leishmania major* protein targets.

Abietane diterpenoids	LmajCatB	LmajDHODH	LmajdUTPase	LmajNDKb	LmajNH	LdonNMT	LmajOPB	LmajPDE1	LmajPTR1	LmajMetRS	LmajTyrRS	LmajUGPase
Abieta−7,13−diene	−71.5	−80.8	−79.3	−80.3	−76.3	−69.7	−82.2	−77.0	−74.7	−96.9	−78.1	−78.3
*ar*−Abietatriene−12,16−diol−14,16−oxide	−74.0	−97.8	−78.9	−96.2	−78.0	−74.8	−98.2	−83.2	−73.8	−89.0	−84.6	−77.8
*ar*−Abietatrien−12−ol−6,7−dione−14,16−oxide	−66.1	−91.8	−86.1	−79.4	−72.8	−74.2	−98.2	−83.6	−84.5	−92.7	−91.4	−83.4
*epi*−Abietic acid	−75.3	−93.5	−81.8	−74.9	−84.0	−78.1	−86.1	−80.7	−82.1	−99.8	−87.3	−84.7
4− *epi*−Abietol	−71.9	−87.6	−81.8	−80.4	−80.9	−69.3	−83.4	−74.8	−78.1	−96.3	−81.0	−82.3
Cryptotanshinone	−69.2	−93.7	−74.6	−81.3	−59.6	−78.3	−91.6	−83.4	−81.2	−105.2	−86.5	−78.6
12− *O*−Deacetyl−6−*O*−acetyl−18−acetyloxycoleon Q	−81.2	−89.0	−95.0	−89.7	−99.9	−79.5	−95.5	−111.8	−93.9	−94.2	−88.9	−114.2
12− *O*−Deacetyl−6−*O*−acetyl−19−acetyloxycoleon Q	−76.3	−104.3	−82.9	−93.8	−101.2	−75.4	−100.7	−107.4	−84.3	−99.0	−92.0	−96.9
12−Deoxyroyleanone	−78.2	−79.2	−74.3	−74.8	−72.9	−73.1	−76.5	−83.6	−86.4	−85.9	−77.6	−77.4
9α,13α− *epi*−Dioxyabiet−8(14)−en−18−oic acid	−65.5	−88.2	−79.9	−82.1	−80.1	−64.0	−89.6	−79.4	−71.2	−89.5	−88.2	−81.6
9α,13α− *epi*−Dioxyabiet−8(14)en−18−ol	−47.3	−86.6	−76.2	−78.7	−72.3	−65.3	−74.4	−76.8	−72.7	−81.5	−74.0	−71.2
Dracocephalone A	−66.5	−82.6	−70.8	−76.6	−71.9	−66.7	−93.0	−83.2	−87.4	−83.4	−72.9	−78.7
Dracocequinone A	−66.6	−84.4	−74.5	−76.0	−74.1	−75.4	−94.8	−83.5	−73.8	−87.6	−76.4	−82.6
Dracocequinone B	−74.5	−89.1	−71.5	−78.4	−78.4	−76.6	−83.4	−81.9	−76.0	−88.7	−77.3	−88.2
Ferruginol	−68.7	−90.3	−81.0	−81.7	−76.0	−71.3	−85.3	−82.2	−83.7	−99.3	−83.4	−81.2
Hinokiol	−77.9	−88.2	−85.2	−75.8	−79.1	−62.3	−84.5	−74.9	−77.9	−92.4	−77.7	−74.0
Hinokiol−1−one	−79.9	−87.2	−87.6	−80.6	−81.8	−70.4	−86.5	−76.7	−81.6	−97.4	−82.2	−77.5
7β−Hydroxyabieta−8,13−diene−11,12−dione	−76.2	−77.8	−94.0	−83.9	−79.9	−76.5	−91.4	−84.1	−81.3	−95.2	−86.2	−83.9
7α−Hydroxyabieta−8,11,13−triene	−76.8	−85.3	−84.5	−75.4	−69.1	−66.7	−83.0	−81.3	−77.8	−90.9	−78.7	−82.0
14−Hydroxy−7,9(11),13−abietatriene−6,12−dione	−74.8	−83.5	−71.0	−82.1	−68.6	−76.5	−88.3	−76.2	−78.6	−94.8	−84.7	−85.5
11−Hydroxy−7,9(11),13−abietatrien−12−one	−72.5	−82.5	−80.1	−82.3	−75.3	−72.1	−83.7	−73.6	−82.4	−95.9	−74.1	−80.4
12−Hydroxy−8,12−abietadiene−3,11,14−trione	−75.9	−86.0	−75.2	−79.9	−83.7	−73.9	−90.3	−84.7	−94.2	−85.3	−79.6	−86.0
1β−Hydroxycryptotanshinone	−74.7	−96.0	−70.0	−80.8	−70.1	−79.2	−99.1	−83.8	−84.0	−104.4	−90.0	−78.5
7−Hydroxy−12−methoxy−20− *nor*−abieta−1,5(10),7,9,12−pentaen−6,14−dione	−66.6	−91.6	−75.4	−80.9	−72.5	−79.2	−92.5	−82.4	−83.6	−94.2	−83.7	−81.5
14−Hydroxy−6−oxoferruginol	−56.1	−91.1	−72.3	−85.5	−75.7	−73.9	−87.0	−79.5	−83.6	−90.3	−75.2	−79.3
6−Hydroxysalvinolone	−77.1	−83.3	−76.6	−76.2	−77.6	−72.4	−82.3	−81.2	−82.7	−93.7	−87.1	−86.5
Komarovinone A	−82.0	−82.4	−79.2	−73.1	−72.7	−67.1	−96.1	−83.5	−84.6	−100.0	−77.6	−84.0
1−Oxocryptotanshinone	−75.8	−102.9	−74.7	−77.9	−70.6	−75.4	−96.6	−79.8	−82.4	−114.6	−89.3	−78.0
1−Oxomiltirone	−71.6	−84.7	−78.4	−77.8	−71.6	−76.8	−90.3	−82.8	−79.8	−104.9	−82.4	−79.1
Royleanone	−73.5	−84.0	−72.2	−81.9	−79.6	−72.3	−90.5	−82.7	−91.0	−87.9	−80.5	−83.9
Sugiol	−67.6	−90.5	−82.4	−73.2	−81.5	−53.1	−76.2	−84.0	−77.2	−97.3	−84.0	−84.4
Taxodione	−79.0	−87.2	−77.8	−83.5	−75.7	−72.3	−85.6	−78.3	−69.0	−90.7	−82.0	−80.4
6,11,12,16−Tetrahydroxy−5,8,11,13−abietatetraen−7−one	−78.2	−88.6	−81.5	−82.9	−80.2	−73.3	−95.1	−87.8	−88.3	−93.8	−83.4	−90.7
6,12,14−Trihydroxyabieta−5,8,11,13−tetraen−7−one	−69.6	−86.1	−70.6	−78.7	−73.9	−80.8	−89.4	−84.6	−81.5	−97.4	−85.9	−83.5
4− *epi*−Triptobenzene L	−68.9	−94.9	−89.9	−81.7	−79.6	−76.6	−87.6	−85.9	−83.3	−88.2	−82.4	−82.4
Uncinatone	−84.3	−81.8	−81.6	−85.9	−62.1	−74.5	−86.5	−86.5	−108.8	−107.7	−95.6	−77.6

**Table 17:** MolDock docking energies (kJ/mol) of abietane diterpenoids with *Leishmania donovani* and *L. mexicana* protein targets.

Abietane diterpenoids	LdonCatB	LdonCyp	LdonDHODH	LdonNMT	LmexGAPDH	LmexGPDH	LmexPGI	LmexPMM	LmexPYK	LmexPYK	LmexPYK	LmexTIM
									Site 1	Site 2	Site 3	
Abieta−7,13−diene	−73.0	−84.2	−70.8	−69.7	−69.1	−92.6	−62.7	−76.6	−81.3	−71.3	−74.2	−62.9
*ar*−Abietatriene−12,16−diol−14,16−oxide	−77.9	−84.2	−70.5	−74.8	−73.7	−104.7	−74.6	−82.6	−104.4	−83.5	−84.9	−73.1
*ar*−Abietatrien−12−ol−6,7−dione−14,16−oxide	−76.3	−90.1	−75.9	−74.2	−69.1	−102.3	−68.9	−93.5	−99.2	−87.3	−90.1	−72.1
*epi−*Abietic acid	−77.4	−86.4	−67.6	−78.1	−60.9	−97.6	−67.8	−81.5	−84.0	−74.5	−91.0	−68.6
4− *epi*−Abietol	−77.2	−88.0	−69.7	−69.3	−68.1	−94.0	−66.3	−78.8	−78.0	−75.2	−72.6	−65.9
Cryptotanshinone	−77.5	−80.5	−84.5	−78.3	−66.6	−101.9	−71.1	−84.4	−95.4	−75.3	−71.7	−65.3
12− *O*−Deacetyl−6−*O*−acetyl−18−acetyloxycoleon Q	−78.6	−93.4	−41.3	−79.5	−86.6	−108.4	−87.4	−109.1	−102.6	−110.1	−102.8	−86.7
12− *O*−Deacetyl−6−*O*−acetyl−19−acetyloxycoleon Q	−99.6	−80.4	−45.8	−75.4	−90.6	−105.1	−87.4	−109.7	−101.1	−96.2	−88.7	−73.6
12−Deoxyroyleanone	−80.4	−86.7	−72.1	−73.1	−68.2	−99.6	−62.7	−82.7	−84.0	−73.0	−87.4	−68.4
9α,13α− *epi*−Dioxyabiet−8(14)−en−18−oic acid	−59.0	−88.1	−51.5	−64.0	−71.2	−100.3	−69.3	−86.9	−82.4	−77.1	−85.3	−76.1
9α,13α− *epi*−Dioxyabiet−8(14)en−18−ol	−55.8	−76.8	−58.2	−65.3	−63.2	−96.6	−66.9	−81.2	−79.7	−71.6	−74.1	−70.9
Dracocephalone A	−74.3	−81.2	−73.4	−66.7	−69.5	−103.5	−64.4	−82.2	−84.9	−74.8	−81.2	−68.1
Dracocequinone A	−71.2	−83.6	−77.7	−75.4	−66.1	−104.7	−66.2	−82.5	−84.7	−72.0	−79.9	−68.9
Dracocequinone B	−75.7	−81.6	−72.0	−76.6	−67.4	−107.2	−67.7	−83.7	−82.8	−72.2	−77.3	−76.7
Ferruginol	−70.5	−91.1	−75.4	−71.3	−71.2	−96.7	−75.1	−83.7	−82.9	−75.3	−76.1	−69.8
Hinokiol	−77.1	−87.8	−72.0	−62.3	−71.1	−95.9	−65.9	−79.3	−81.4	−71.9	−73.5	−63.7
Hinokiol−1−one	−77.0	−86.1	−72.7	−70.4	−70.9	−99.6	−63.7	−80.2	−79.7	−69.7	−77.2	−67.6
7β−Hydroxyabieta−8,13−diene−11,12−dione	−83.8	−79.8	−80.7	−76.5	−69.0	−100.8	−69.4	−89.6	−91.6	−77.9	−83.9	−76.8
7α−Hydroxyabieta−8,11,13−triene	−75.4	−76.3	−73.1	−66.7	−68.1	−96.5	−70.5	−78.2	−82.5	−74.8	−82.9	−67.0
14−Hydroxy−7,9(11),13−abietatriene−6,12−dione	−74.5	−89.5	−81.6	−76.5	−72.7	−96.0	−68.6	−86.8	−85.3	−76.6	−75.3	−67.9
11−Hydroxy−7,9(11),13−abietatrien−12−one	−79.6	−87.1	−76.1	−72.1	−61.8	−97.7	−68.7	−83.9	−78.1	−76.3	−78.1	−69.9
12−Hydroxy−8,12−abietadiene−3,11,14−trione	−79.7	−82.0	−75.6	−73.9	−66.2	−109.4	−68.9	−92.5	−88.8	−75.3	−84.8	−71.0
1b−Hydroxycryptotanshinone	−78.4	−84.0	−80.5	−79.2	−68.3	−98.5	−71.1	−87.5	−94.5	−77.8	−74.5	−70.8
7−hydroxy−12−methoxy−20− *nor*−abieta−1,5(10),7,9,12−pentaen−6,14−dione	−75.6	−87.7	−69.1	−79.2	−67.5	−102.0	−68.2	−87.1	−88.6	−68.1	−79.9	−65.2
14−Hydroxy−6−oxoferruginol	−66.5	−93.7	−78.2	−73.9	−71.7	−96.9	−61.8	−87.2	−88.7	−80.8	−70.6	−68.5
6−Hydroxysalvinolone	−78.8	−78.9	−77.6	−72.4	−62.2	−94.7	−66.0	−89.9	−94.9	−84.3	−75.8	−69.0
Komarovinone A	−80.3	−81.2	−81.2	−67.1	−65.0	−98.3	−69.2	−90.1	−86.5	−80.4	−70.8	−73.4
1−Oxocryptotanshinone	−76.1	−80.4	−71.9	−75.4	−66.9	−95.0	−69.1	−85.7	−89.6	−79.8	−77.1	−65.4
1−Oxomiltirone	−74.3	−71.4	−78.1	−76.8	−61.6	−92.9	−65.3	−81.5	−84.5	−76.6	−71.2	−66.5
Royleanone	−80.1	−85.8	−75.6	−72.3	−61.3	−102.2	−65.3	−87.9	−90.8	−77.4	−73.0	−69.1
Sugiol	−73.9	−78.4	−77.3	−53.1	−65.1	−95.8	−66.2	−85.3	−86.3	−79.1	−71.1	−71.8
Taxodione	−80.6	−94.1	−77.9	−72.3	−68.0	−97.3	−70.5	−89.2	−81.6	−79.0	−75.8	−49.5
6,11,12,16−Tetrahydroxy−5,8,11,13−abietatetra−en−7−one	−79.0	−86.0	−84.9	−73.3	−66.9	−96.1	−65.9	−95.6	−105.1	−87.5	−77.6	−75.5
6,12,14−Trihydroxyabieta−5,8,11,13−tetraen−7−one	−72.7	−84.9	−81.2	−80.8	−72.6	−97.7	−62.5	−89.1	−89.8	−89.2	−77.1	−62.8
4− *epi*−Triptobenzene L	−76.3	−89.7	−76.3	−76.6	−72.3	−101.5	−70.0	−85.1	−85.7	−77.6	−77.9	−72.5
Uncinatone	−85.4	−73.3	−72.1	−74.5	−69.8	−89.0	−65.3	−84.4	−94.8	−84.1	−89.2	−64.4

**Table 18:** MolDock docking energies (kJ/mol) of abietane diterpenoids with *Leishmania infantum* protein targets.

Abietane diterpenoids	LinfCYP51	LinfGLO2	LinfPnC1	LinfTDR1	LinfTR
Abieta−7,13−diene	−76.4	−71.1	no dock	−65.7	−72.6
*ar*−Abietatriene−12,16−diol−14,16−oxide	−87.3	−76.4	no dock	−74.3	−78.4
*ar*−Abietatrien−12−ol−6,7−dione−14,16−oxide	−84.7	−72.7	no dock	−76.8	−83.5
*epi*−Abietic acid	−83.8	−67.7	no dock	−66.4	−74.5
4− *epi*−Abietol	−78.5	−68.9	no dock	−64.6	−72.4
Cryptotanshinone	−83.6	−67.0	no dock	−71.5	−84.1
12− *O*−Deacetyl−6−*O*−acetyl−18−acetyloxycoleon Q	−106.3	−80.7	no dock	−88.6	−103.4
12− *O*−Deacetyl−6−*O*−acetyl−19−acetyloxycoleon Q	−105.1	−85.8	no dock	−87.3	−98.2
12−Deoxyroyleanone	−81.7	−72.8	no dock	−67.2	−77.2
9α,13α− *epi*−Dioxyabiet−8(14)−en−18−oic acid	−90.4	−73.1	no dock	−68.7	−77.1
9α,13α− *epi*−Dioxyabiet−8(14)en−18−ol	−80.3	−70.1	no dock	−65.9	−72.3
Dracocephalone A	−83.2	−64.1	no dock	−76.6	−82.5
Dracocequinone A	−82.5	−63.6	no dock	−77.7	−74.9
Dracocequinone B	−85.6	−69.1	no dock	−76.4	−75.5
Ferruginol	−78.9	−71.0	no dock	−73.2	−79.0
Hinokiol	−77.7	−68.5	no dock	−62.4	−70.0
Hinokiol−1−one	−84.6	−69.6	no dock	−72.9	−72.4
7β−Hydroxyabieta−8,13−diene−11,12−dione	−85.1	−71.6	no dock	−73.0	−85.6
7α−Hydroxyabieta−8,11,13−triene	−79.5	−68.9	no dock	−67.6	−77.1
14−Hydroxy−7,9(11),13−abietatriene−6,12−dione	−82.9	−71.7	no dock	−71.5	−84.5
11−Hydroxy−7,9(11),13−abietatrien−12−one	−81.1	−68.0	no dock	−69.6	−73.3
12−Hydroxy−8,12−abietadiene−3,11,14−trione	−83.4	−76.4	no dock	−73.8	−86.9
1β−Hydroxycryptotanshinone	−84.1	−66.5	no dock	−75.5	−88.2
7−Hydroxy−12−methoxy−20− *nor*−abieta−1,5(10),7,9,12−pentaen−6,14−dione	−84.6	−67.7	no dock	−73.0	−78.3
14−Hydroxy−6−oxoferruginol	−85.2	−73.0	no dock	−71.7	−79.3
6−Hydroxysalvinolone	−81.2	−68.6	no dock	−80.7	−85.5
Komarovinone A	−86.9	−66.3	no dock	−74.9	−84.7
1−Oxocryptotanshinone	−83.9	−66.9	no dock	−72.6	−84.1
1−Oxomiltirone	−85.5	−64.4	no dock	−69.7	−78.6
Royleanone	−82.6	−72.0	no dock	−72.8	−82.1
Sugiol	−81.8	−70.4	no dock	−72.6	−78.7
Taxodione	−81.2	−68.2	no dock	−71.1	−75.8
6,11,12,16−Tetrahydroxy−5,8,11,13−abietatetraen−7−one	−88.5	−66.5	no dock	−85.7	−93.8
6,12,14−Trihydroxyabieta−5,8,11,13−tetraen−7−one	−83.5	−73.4	no dock	−75.9	−81.9
4− *epi*−Triptobenzene L	−83.0	−72.3	no dock	−65.2	−76.3
Uncinatone	−93.0	−73.3	no dock	−82.5	−85.5

**Table 19:** MolDock docking energies (kJ/mol) of clerodane diterpenoids with *Leishmania major* protein targets.

Clerodane diterpenoids	LmajCatB	LmajDHODH	LmajdUTPase	LmajNDKb	LmajNH	LdonNMT	LmajOPB	LmajPDE1	LmajPTR1	LmajMetRS	LmajTyrRS	LmajUGPase
15−Acetoxy−*cis*−cleroden−3−en−18−al	−68.6	−94.1	−85.0	−97.9	−96.0	−87.3	−90.4	−94.9	−94.7	−107.1	−92.6	−106.9
15−Acetoxy−*cis*−cleroden−3−en−18−oic acid	−75.0	−103.1	−88.3	−97.2	−92.3	−87.0	−91.6	−95.3	−93.2	−97.7	−94.4	−97.3
18−Acetoxy−*cis*−cleroden−3−en−15−oic acid	−76.9	−103.7	−82.7	−96.7	−88.8	−80.7	−97.3	−96.8	−89.3	−104.2	−97.5	−98.2
15−*O*−Acetylcistadiol	−68.0	−94.7	−87.4	−97.3	−94.7	−80.4	−96.8	−89.2	−92.9	−106.7	−95.0	−95.3
18−*O*−Acetylcistadiol	−70.5	−103.4	−88.1	−91.7	−93.2	−85.0	−94.0	−93.8	−82.3	−101.2	−95.9	−100.3
Cistadiol	−64.1	−93.4	−79.7	−81.6	−81.9	−69.0	−77.1	−85.2	−79.2	−78.9	−80.7	−92.5
*trans*−Dehydrocrotonin	−78.6	−114.9	−90.2	−91.8	−84.5	−75.9	−89.0	−89.1	−99.2	−115.1	−97.7	−92.1
15,18−Di−*O*−acetylcistadiol	−77.8	−100.5	−92.8	−109.4	−94.8	−84.2	−90.5	−107.0	−91.6	−117.5	−100.6	−110.4
8−*epi*−Kolavenol	−71.5	−94.5	−79.2	−80.4	−85.0	−72.3	−78.7	−80.6	−82.8	−92.9	−85.3	−95.3
*epi*−Populifolic acid	−69.9	−85.5	−72.7	−82.3	−83.4	−71.7	−76.3	−82.2	−78.3	−85.0	−78.2	−89.9
*ent*−16*R*−Hydroxy−3,13−clerodadien−15,16−olide	−77.7	−108.7	−86.4	−88.2	−87.7	−88.2	−90.8	−87.5	−101.9	−105.9	−104.9	−97.4
*ent*−16*S*−Hydroxy−3,13−clerodadien−15,16−olide	−81.9	−101.9	−84.6	−91.0	−83.9	−78.4	−93.4	−82.2	−100.3	−110.7	−99.4	−105.1
15−Hydroxy−*cis*−cleroden−3−en−18−al	−66.5	−91.8	−77.4	−81.8	−86.8	−76.7	−76.8	−83.7	−77.5	−92.8	−80.1	−91.4
15−Hydroxy−*cis*−cleroden−3−en−18−oic acid	−69.0	−95.8	−90.1	−83.5	−86.9	−80.5	−82.1	−86.0	−80.4	−95.9	−84.3	−95.2
18−Hydroxy−*cis*−cleroden−3−en−15−oic acid	−70.0	−93.4	−76.1	−86.3	−86.8	−76.2	−79.7	−87.2	−81.1	−96.1	−82.9	−97.4
*ent*−12−Oxo−3,13(16)−clerodien−15−oic acid	−75.0	−112.7	−86.3	−90.0	−93.2	−88.2	−87.7	−83.0	−96.5	−107.2	−87.6	−110.5

**Table 20:** MolDock docking energies (kJ/mol) of clerodane diterpenoids with *Leishmania donovani* and *L. mexicana* protein targets.

Clerodane diterpenoids	LdonCatB	LdonCyp	LdonDHODH	LdonNMT	LmexGAPDH	LmexGPDH	LmexPGI	LmexPMM	LmexPYK	LmexPYK	LmexPYK	LmexTIM
									Site 1	Site 2	Site 3	
15−Acetoxy−*cis*−cleroden−3−en−18−al	−80.4	−84.1	−75.1	−87.3	−76.7	−107.2	−75.4	−101.2	−96.3	−96.5	−89.3	−84.6
15−Acetoxy−*cis*−cleroden−3−en−18−oic acid	−83.5	−85.0	−75.2	−87.0	−86.3	−93.6	−84.4	−104.8	−100.2	−92.9	−90.4	−80.9
18−Acetoxy−*cis*−cleroden−3−en−15−oic acid	−82.9	−90.6	−85.1	−80.7	−77.3	−109.2	−77.6	−97.4	−95.9	−90.8	−89.5	−95.5
15−O−Acetylcistadiol	−82.8	−79.6	−72.7	−80.4	−77.4	−102.7	−77.8	−98.9	−96.3	−96.8	−87.7	−89.7
18−O−Acetylcistadiol	−79.8	−92.7	−66.9	−85.0	−73.2	−105.6	−78.9	−97.1	−92.5	−96.8	−92.6	−98.6
Cistadiol	−66.6	−81.0	−58.3	−69.0	−70.5	−91.1	−70.4	−88.0	−90.4	−82.8	−82.5	−80.2
*trans*−Dehydrocrotonin	−92.8	−98.7	−51.3	−75.9	−73.0	−100.5	−77.1	−94.2	−107.8	−87.6	−92.4	−95.2
15,18−Di−*O*−acetylcistadiol	−82.2	−82.4	−94.8	−84.2	−85.2	−122.2	−86.7	−104.1	−102.9	−101.7	−97.4	−99.1
8−*epi*−Kolavenol	−75.5	−83.0	−77.8	−72.3	−71.7	−100.8	−70.6	−85.6	−92.1	−84.2	−94.6	−78.9
*epi*−Populifolic acid	−68.3	−75.8	−63.4	−71.7	−73.8	−89.4	−69.7	−88.7	−91.1	−79.3	−85.0	−82.4
*ent*−16*R*−Hydroxy−3,13−clerodadien−15,16−olide	−74.7	−89.1	−82.9	−88.2	−85.4	−105.8	−79.8	−96.5	−103.1	−89.0	−95.5	−83.1
*ent*−16*S*−Hydroxy−3,13−clerodadien−15,16−olide	−88.6	−91.8	−82.7	−78.4	−83.9	−100.1	−81.3	−97.2	−102.0	−92.3	−90.3	−85.8
15−Hydroxy−*cis*−cleroden−3−en−18−al	−77.5	−87.9	−67.4	−76.7	−74.1	−97.2	−70.9	−87.6	−96.8	−86.4	−85.4	−82.4
15−Hydroxy−*cis*−cleroden−3−en−18−oic acid	−71.4	−86.4	−66.4	−80.5	−74.9	−99.5	−73.3	−85.9	−99.3	−88.1	−88.5	−80.5
18−Hydroxy−*cis*−cleroden−3−en−15−oic acid	−72.5	−84.3	−59.0	−76.2	−78.2	−95.4	−73.2	−90.2	−89.2	−83.9	−90.5	−88.0
*ent*−12−Oxo−3,13(16)−clerodien−15−oic acid	−84.3	−95.2	−72.0	−88.2	−85.3	−88.0	−76.6	−95.9	−108.8	−88.3	−91.6	−86.9

**Table 21:** MolDock docking energies (kJ/mol) of clerodane and labdane diterpenoids with *Leishmania infantum* protein targets.

Clerodane diterpenoids	LinfCYP51	LinfGLO2	LinfPnC1	LinfTDR1	LinfTR
15−Acetoxy−*cis*−cleroden−3−en−18−al	−96.8	−78.2	no dock	−90.4	−91.8
15−Acetoxy−*cis*−cleroden−3−en−18−oic acid	−93.0	−82.7	no dock	−87.6	−91.7
18−Acetoxy−*cis*−cleroden−3−en−15−oic acid	−98.2	−78.1	no dock	−87.6	−97.6
15−O−Acetylcistadiol	−101.7	−89.8	no dock	−88.4	−94.3
18−O−Acetylcistadiol	−95.8	−78.3	no dock	−88.4	−93.8
Cistadiol	−86.8	−68.7	no dock	−78.3	−82.2
*trans*−Dehydrocrotonin	−89.6	−74.5	no dock	−77.6	−88.9
15,18−Di−*O*−acetylcistadiol	−95.6	−85.8	no dock	−89.2	−103.5
8−*epi*−Kolavenol	−83.6	−72.3	−18.2	−80.5	−78.8
*epi*−Populifolic acid	−83.8	−71.4	no dock	−75.9	−77.0
*ent*−16*R*−Hydroxy−3,13−clerodadien−15,16−olide	−87.9	−75.1	−22.6	−83.9	−87.5
*ent*−16*S*−Hydroxy−3,13−clerodadien−15,16−olide	−88.4	−67.5	no dock	−79.6	−78.8
15−Hydroxy−*cis*−cleroden−3−en−18−al	−83.0	−68.9	no dock	−81.0	−81.2
15−Hydroxy−*cis*−cleroden−3−en−18−oic acid	−89.4	−66.8	no dock	−78.4	−78.1
18−Hydroxy−*cis*−cleroden−3−en−15−oic acid	−87.5	−69.6	−31.7	−82.8	−81.3
Labdane diterpenoids					
*ent*−3β−Acetoxy−13−*epi*−manoyl−oxide	−94.3	−72.4	no dock	−67.4	−88.0
Andrographolide	−97.9	−80.4	no dock	−81.1	−91.9
14(*R*)−Aulacocarpin C					
14(*S*)−Aulacocarpin C	−93.1	−81.7	no dock	−81.7	−92.8
Aulacocarpin D	−92.7	−74.5	no dock	−85.8	−93.5
*trans*−Communic acid	−90.8	−75.0	no dock	−82.6	−91.6
*trans*−Communic acid methyl ester	−84.3	−77.2	no dock	−76.0	−82.7
Copalic acid	−89.8	−74.8	no dock	−72.8	−86.8
Dehydropinifolic acid 15−methyl ester	−93.9	−84.1	no dock	−78.1	−83.6
12(*S*)−Hydroxy−15(*R*)−methoxy−labdan−8(17),13(14)−dien−15,16−olide	−93.3	−84.8	no dock	−89.8	−93.5
12(*S*)−Hydroxy−15(*S*)−methoxy−labdan−8(17,)13(14)−dien−15,16−olide	−99.3	−88.5	no dock	−88.8	−95.1
Labda−8(17),12−diene−15,16−dial	−94.2	−84.8	no dock	−89.2	−100.2
13(*E*)−Labda−7,13−dien−8α,15−diol	−87.7	−76.3	no dock	−81.7	−86.6
Labda−12,14−dien−7α,8α−diol	−89.1	−77.8	no dock	−79.8	−88.4
Labdan−8α,15−diol	−85.9	−77.1	no dock	−76.1	−87.2
Labd−8(17)−en−3β,15−diol	−85.8	−77.4	no dock	−82.0	−90.2
13(*E*)−Labd−13−en−8α,15−diol	−84.2	−76.9	no dock	−79.7	−87.2
Lambertianic acid	−87.0	−75.4	no dock	−78.4	−83.4
15(*R*)−Methoxy−labdan−8(17),11(E),13(14)−trien−15,16−olide	−90.0	−73.6	no dock	−72.5	−89.3
15(*S*)−Methoxy−labdan−8(17),11(E),13(14)−trien−15,16−olide	−94.2	−87.8	no dock	−88.3	−92.3
*ent*−12−Oxo−8,13(16)−labdadien−15−oic acid	−96.0	−86.3	no dock	−85.3	−96.8
*ent*−3β−Acetoxy−13−*epi*−manoyl−oxide	−94.1	−77.8	no dock	−78.5	−88.9

**Table 22:** MolDock docking energies (kJ/mol) of labdane diterpenoids with *Leishmania major* protein targets.

Labdane diterpenoids	LmajCatB	LmajDHODH	LmajdUTPase	LmajNDKb	LmajNH	LdonNMT	LmajOPB	LmajPDE1	LmajPTR1	LmajMetRS	LmajTyrRS	LmajUGPase
*ent*−3β−Acetoxy−13−*epi*−manoyl−oxide	−74.9	−96.4	−76.3	−82.2	−82.7	−78.7	−90.7	−82.0	−89.0	−85.9	−80.1	−91.2
Andrographolide	−86.7	−114.6	−90.5	−92.1	−96.5	−101.8	−98.5	−94.4	−101.6	−114.1	−97.7	−95.9
14(*R*)−Aulacocarpin C	−74.4	−116.1	−85.2	−91.7	−97.7	−99.7	−95.7	−85.2	−98.1	−107.0	−91.8	−103.3
14(*S*)−Aulacocarpin C	−83.6	−114.7	−88.3	−90.3	−91.7	−97.4	−90.6	−83.1	−99.9	−107.9	−94.0	−100.3
Aulacocarpin D	−80.8	−108.3	−89.0	−87.3	−87.3	−86.0	−91.8	−98.6	−92.9	−108.3	−93.8	−98.9
*trans*−Communic acid	−69.5	−109.4	−86.3	−86.7	−87.5	−89.7	−83.7	−84.0	−88.4	−97.1	−88.2	−88.3
*trans*−Communic acid methyl ester	−81.3	−103.5	−84.7	−86.3	−85.6	−84.1	−85.7	−83.0	−92.3	−99.6	−89.8	−88.7
Copalic acid	−69.5	−102.7	−88.4	−89.5	−83.2	−83.9	−86.0	−96.2	−90.1	−105.1	−93.9	−104.6
Dehydropinifolic acid 15−methyl ester	−87.3	−116.3	−102.2	−90.1	−87.9	−91.3	−101.0	−89.8	−89.7	−104.1	−99.8	−103.3
12(*S*)−Hydroxy−15(*R*)−methoxy−labdan−8(17),13(14)−dien−15,16−olide	−85.8	−114.5	−87.2	−97.5	−88.7	−93.8	−96.4	−88.0	−96.1	−108.9	−103.0	−110.1
12(*S*)−Hydroxy−15(*S*)−methoxy−labdan−8(17,)13(14)−dien−15,16−olide	−90.2	−112.6	−89.4	−98.7	−98.8	−84.0	−107.3	−89.1	−91.2	−110.4	−103.6	−111.4
Labda−8(17),12−diene−15,16−dial	−77.5	−107.2	−87.2	−82.9	−93.0	−95.8	−82.8	−89.0	−97.5	−103.2	−95.5	−102.8
13(*E*)−Labda−7,13−dien−8α,15−diol	−76.9	−100.6	−85.8	−87.0	−86.3	−89.7	−100.7	−85.8	−87.9	−107.5	−91.0	−90.8
Labda−12,14−dien−7α,8α−diol	−71.9	−99.4	−76.2	−75.8	−89.8	−89.8	−90.5	−85.1	−81.5	−101.3	−87.8	−94.7
Labdan−8α,15−diol	−78.9	−101.4	−84.6	−81.5	−80.3	−92.1	−93.7	−82.7	−86.0	−108.6	−83.9	−92.3
Labd−8(17)−en−3β,15−diol	−75.5	−106.7	−82.2	−82.9	−80.6	−71.0	−88.0	−81.0	−93.9	−104.9	−85.9	−90.8
13(*E*)−Labd−13−en−8α,15−diol	−69.4	−105.0	−82.1	−83.3	−85.1	−89.1	−88.5	−85.8	−88.9	−107.8	−89.8	−92.2
Lambertianic acid	−69.8	−110.5	−86.3	−82.0	−84.0	−79.8	−81.8	−83.9	−93.5	−100.7	−87.7	−88.9
15(*R*)−Methoxy−labdan−8(17),11(E),13(14)−trien−15,16−olide	−88.3	−111.4	−84.5	−97.3	−91.3	−91.7	−104.9	−94.4	−91.8	−101.5	−95.0	−98.9
15(*S*)−Methoxy−labdan−8(17),11(E),13(14)−trien−15,16−olide	−83.1	−104.7	−84.8	−89.0	−95.4	−88.5	−109.2	−98.3	−99.0	−106.2	−98.3	−97.3
*ent*−12−Oxo−8,13(16)−labdadien−15−oic acid	−78.0	−107.7	−93.4	−92.3	−88.2	−92.1	−90.6	−90.0	−94.1	−100.0	−97.0	−100.4

**Table 23:** MolDock docking energies (kJ/mol) of labdane diterpenoids with *Leishmania donovani* and *L. mexicana* protein targets.

	LdonCatB	LdonCyp	LdonDHODH	LdonNMT	LmexGAPDH	LmexGPDH	LmexPGI	LmexPMM	LmexPYK	LmexPYK	LmexPYK	LmexTIM
Labdane diterpenoids									Site 1	Site 2	Site 3	
*ent*−3β−Acetoxy−13−*epi*−manoyl−oxide	−83.3	−77.6	−60.4	−78.7	−67.9	−94.4	−69.1	−86.9	−90.2	−84.0	−77.7	−78.3
Andrographolide	−88.9	−93.2	−94.9	−101.8	−86.1	−114.0	−79.6	−100.7	−110.3	−100.3	−107.0	−95.5
14(*R*)−Aulacocarpin C	−87.0	−98.3	−92.5	−99.7	−78.4	−103.3	−76.8	−95.7	−99.6	−91.7	−114.0	−84.2
14(*S*)−Aulacocarpin C	−85.6	−95.6	−90.3	−97.4	−75.0	−100.7	−77.8	−90.6	−97.1	−95.8	−109.0	−86.7
Aulacocarpin D	−96.6	−88.1	−75.2	−86.0	−87.6	−99.9	−73.6	−87.5	−95.9	−89.8	−88.3	−87.8
*trans*−Communic acid	−77.9	−87.9	−79.6	−89.7	−70.6	−100.8	−75.8	−88.3	−92.3	−82.9	−98.3	−83.1
*trans*−Communic acid methyl ester	−85.8	−90.8	−81.9	−84.1	−73.5	−104.8	−72.2	−88.1	−95.4	−82.4	−90.0	−85.2
Copalic acid	−89.0	−92.6	−85.1	−83.9	−73.8	−90.3	−76.9	−92.0	−94.8	−88.0	−92.4	−97.5
Dehydropinifolic acid 15−methyl ester	−85.8	−95.2	−88.4	−91.3	−84.5	−123.2	−85.7	−100.6	−111.6	−93.2	−104.2	−99.0
12(*S*)−Hydroxy−15(*R*)−methoxy−labdan−8(17),13(14)−dien−15,16−olide	−92.1	−93.8	−90.8	−93.8	−75.2	−117.2	−79.4	−101.2	−117.2	−96.5	−104.7	−93.8
12(*S*)−Hydroxy−15(*S*)−methoxy−labdan−8(17,)13(14)−dien−15,16−olide	−95.4	−92.4	−82.7	−84.0	−88.1	−116.2	−91.2	−103.1	−117.1	−92.4	−109.7	−93.1
Labda−8(17),12−diene−15,16−dial	−90.6	−93.4	−85.7	−95.8	−75.4	−97.3	−72.3	−93.0	−97.0	−87.4	−99.5	−85.4
13(*E*)−Labda−7,13−dien−8α,15−diol	−88.4	−82.3	−80.9	−89.7	−76.3	−109.7	−73.5	−87.1	−100.2	−82.1	−93.7	−88.8
Labda−12,14−dien−7α,8α−diol	−86.9	−83.8	−80.5	−89.8	−77.9	−86.3	−78.7	−89.2	−100.4	−85.5	−91.2	−91.6
Labdan−8α,15−diol	−86.8	−82.0	−82.5	−92.1	−73.2	−94.9	−76.4	−95.9	−93.7	−86.0	−90.8	−88.8
Labd−8(17)−en−3β,15−diol	−78.5	−85.4	−86.9	−71.0	−71.0	−87.8	−72.0	−93.5	−97.3	−81.6	−93.8	−86.2
13(*E*)−Labd−13−en−8α,15−diol	−81.4	−82.1	−77.1	−89.1	−71.9	−104.1	−83.4	−88.7	−99.2	−84.5	−92.7	−92.0
Lambertianic acid	−82.2	−82.9	−81.6	−79.8	−72.9	−109.5	−75.1	−95.1	−96.2	−85.6	−98.4	−80.3
15(*R*)−Methoxy−labdan−8(17),11(E),13(14)−trien−15,16−olide	−106.6	−91.2	−92.5	−91.7	−79.3	−121.2	−76.7	−97.1	−105.9	−94.3	−95.6	−91.2
15(*S*)−Methoxy−labdan−8(17),11(E),13(14)−trien−15,16−olide	−92.3	−92.2	−93.3	−88.5	−87.3	−115.8	−78.5	−94.5	−101.8	−97.0	−89.2	−91.5
*ent*−12−Oxo−8,13(16)−labdadien−15−oic acid	−76.2	−84.3	−84.0	−92.1	−89.2	−99.0	−72.6	−102.2	−105.2	−91.1	−103.5	−91.8

**Table 24:** MolDock docking energies (kJ/mol) of kaurane and pimarane diterpenoids with *Leishmania major* protein targets.

Kaurane diterpenoids	LmajCatB	LmajDHODH	LmajdUTPase	LmajNDKb	LmajNH	LdonNMT	LmajOPB	LmajPDE1	LmajPTR1	LmajMetRS	LmajTyrRS	LmajUGPase
15−Angeloyl−4α,15β−kaur−16−en−18−oic acid	−88.6	−116.3	−75.8	−97.5	−97.3	−78.2	−85.5	−98.6	−97.4	−116.0	−91.4	−106.3
*ent*−11α−Hydroxy−16−kauren−15−one	−64.3	−98.8	−69.7	−76.7	−83.3	−64.7	−87.8	−83.0	−74.0	−85.7	−75.1	−81.0
Kaurenoic acid	−70.4	−95.2	−77.8	−76.3	−81.4	−58.4	−86.5	−80.6	−89.7	−97.5	−75.5	−83.2
Perymenic acid	−97.6	−115.4	−75.2	−95.4	−103.0	−81.2	−88.9	−99.6	−98.5	−118.4	−91.2	−103.7
*ent*−15β−Senecioyloxy−16,17−epoxy−kauran−18−oic acid	−97.3	−120.5	−76.6	−93.6	−102.4	−87.8	−100.7	−100.8	−86.6	−111.7	−93.3	−104.6
**Pimarane diterpenoids**												
Acanthoic acid	−66.0	−97.0	−73.1	−84.0	−86.6	−72.3	−87.9	−81.1	−78.7	−87.9	−72.7	−82.5
7β−Hydroxy− *ent*−pimara−8(14),15−dien−19−oic acid	−74.0	−93.7	−79.3	−84.3	−87.3	−71.7	−92.6	−87.7	−76.7	−87.6	−78.9	−81.2
*ent*−15−Pimarene−8β,19−diol	−72.1	−97.1	−69.0	−78.0	−86.3	−66.1	−87.4	−79.1	−77.5	−84.4	−74.3	−85.5
*ent*−8(14),15−Pimaradien−3β−acetoxy	−72.5	−85.3	−83.9	−90.3	−89.4	−71.7	−88.6	−82.1	−83.7	−99.7	−88.9	−92.1
*ent*−8(14),15−Pimaradien−3β,19−diol	−66.9	−90.8	−66.2	−78.3	−91.3	−71.5	−83.3	−80.0	−75.0	−86.6	−80.9	−81.6
*ent*−Pimara−8(14),15−dien−19−oic acid	−65.8	−91.7	−69.9	−81.3	−87.1	−70.6	−89.3	−80.2	−81.5	−85.7	−75.7	−77.4
*ent*−8(14),15−Pimaradien−3β−ol	−64.7	−89.0	−66.5	−77.7	−86.2	−66.6	−82.3	−76.4	−73.8	−79.4	−81.8	−76.4

**Table 25:** MolDock docking energies (kJ/mol) of kaurane and pimarane diterpenoids with *Leishmania donovani* and *L. mexicana* protein targets.

	LdonCatB	LdonCyp	LdonDHODH	LdonNMT	LmexGAPDH	LmexGPDH	LmexPGI	LmexPMM	LmexPYK	LmexPYK	LmexPYK	LmexTIM
Kaurane diterpenoids									Site 1	Site 2	Site 3	
15−Angeloyl−4α,15β−kaur−16−en−18−oic acid	−97.4	−78.7	−27.8	−78.2	−91.8	−121.4	−75.9	−96.9	−104.0	−89.2	−92.8	−77.3
*ent*−11α−Hydroxy−16−kauren−15−one	−66.2	−82.3	no dock	−64.7	−61.9	−84.4	−63.0	−81.1	−79.6	−73.8	−70.1	−80.5
Kaurenoic acid	−69.6	−73.9	−21.6	−58.4	−64.6	−88.8	−64.5	−80.3	−93.9	−77.4	−81.3	−78.1
Perymenic acid	−100.6	−92.3	−45.0	−81.2	−85.7	−128.3	−76.8	−100.4	−99.7	−95.7	−91.9	−80.3
*ent*−15β−Senecioyloxy−16,17−epoxy−kauran−18−oic acid	−99.1	−88.1	−38.0	−87.8	−90.1	−128.9	−77.2	−96.5	−99.3	−92.9	−98.0	−81.1
**Pimarane diterpenoids**												
Acanthoic_acid	−69.3	−79.7	−62.5	−72.3	−59.8	−90.8	−69.0	−81.5	−92.4	−79.2	−78.3	−79.4
7β−Hydroxy− *ent*−pimara−8(14),15−dien−19−oic acid	−71.2	−78.8	−13.1	−71.7	−71.7	−101.0	−68.7	−88.0	−96.6	−83.5	−76.9	−71.3
*ent*−15−Pimarene−8β,19−diol	−75.9	−77.9	−63.4	−66.1	−72.6	−85.2	−68.6	−79.0	−79.7	−80.1	−71.6	−79.0
*ent*−8(14),15−Pimaradien−3β−acetoxy	−79.6	−80.5	−36.8	−71.7	−71.4	−90.2	−63.2	−83.7	−88.7	−84.4	−73.5	−76.4
*ent*−8(14),15−Pimaradien−3β,19−diol	−67.6	−73.6	−31.7	−71.5	−65.3	−90.2	−69.2	−83.6	−95.0	−79.6	−73.6	−82.7
*ent*−Pimara−8(14),15−dien−19−oic acid	−66.6	−77.7	−53.0	−70.6	−64.0	−94.0	−66.0	−85.4	−88.0	−79.7	−70.8	−77.1
*ent*−8(14),15−Pimaradien−3b−ol	−69.3	−71.6	−26.5	−66.6	−63.1	−88.7	−64.5	−80.7	−94.3	−77.1	−72.6	−79.9

**Table 26:** MolDock docking energies (kJ/mol) of miscellaneous diterpenoids with *Leishmania major* protein targets.

	LmajCatB	LmajDHODH	LmajdUTPase	LmajNDKb	LmajNH	LdonNMT	LmajOPB	LmajPDE1	LmajPTR1	LmajMetRS	LmajTyrRS	LmajUGPase
Cassane diterpenoids												
6β− *O*−Cinnamoyl−12−hydroxy−(13)15−en−16,12−olide−18−cassaneoic acid	−78.8	−123.0	−110.4	−111.0	−119.5	−108.1	−107.4	−108.1	−120.0	−134.4	−115.8	−116.4
6α,7β−Diacetoxyvouacapane	−77.8	−118.7	−80.7	−91.0	−92.6	−74.7	−85.6	−99.8	−86.5	−90.4	−88.3	−88.0
6β− *O*−2*'*3*'*−Dihydrocinnamoyl−12−hydroxy−(13)15−en−16,12−olide−18−cassaneoic acid	−107.0	−125.0	−97.1	−107.3	−121.6	−101.3	−109.0	−109.0	−117.3	−135.2	−115.1	−114.3
**Icetaxane diterpenoids**												
Cyclocoulterone	−70.3	−92.1	−83.7	−88.6	−78.7	−81.3	−86.7	−96.1	−91.6	−100.2	−91.8	−92.0
5− *epi*−Icetexone	−80.9	−91.0	−74.2	−81.6	−88.3	−27.2	−95.7	−88.5	−88.5	−96.9	−98.8	−85.9
Komaroviquinone	−75.7	−83.0	−87.6	−84.9	−75.6	−76.5	−90.9	−98.1	−97.8	−105.2	−87.8	−87.0
**Mulinane diterpenoids**												
Azorellanol	−64.7	−107.8	−74.6	−79.1	−98.7	−80.0	−94.5	−99.7	−85.3	−98.6	−88.5	−91.9
7−Deacetylazorellanol	−72.5	−98.4	−72.3	−80.2	−79.0	−72.4	−88.9	−91.8	−76.2	−94.0	−90.1	−78.7
Mulin−11,13−dien−20−oic acid	−83.1	−103.0	−78.0	−84.3	−80.7	−71.5	−87.5	−83.5	−87.0	−100.5	−88.1	−88.3
Mulinic acid	−69.4	−98.9	−71.9	−82.4	−80.7	−82.3	−83.6	−85.7	−74.6	−93.7	−78.3	−97.0
Mulinolic acid	−75.6	−94.5	−87.3	−82.5	−84.5	−85.1	−84.9	−90.2	−81.1	−102.3	−92.2	−88.7
**Miscellaneous diterpenoids**												
Hypoestoxide	−74.1	−117.7	−88.3	−80.1	−87.8	−85.2	−94.0	−97.0	−78.1	−81.1	−99.5	−99.2
Komarovispirone	−74.1	−92.9	−75.8	−88.9	−90.5	−75.5	−85.4	−86.4	−75.3	−79.9	−94.0	−91.4
Sacculatal	−69.5	−112.7	−85.2	−87.1	−86.1	−82.4	−88.0	−85.9	−104.4	−103.5	−95.2	−100.4
Serratol	−69.2	−90.5	−75.7	−77.2	−78.8	−77.4	−82.1	−89.7	−79.1	−93.2	−90.3	−88.6
Totarol	−62.7	−96.6	−64.9	−85.1	−76.4	−69.8	−89.2	−80.7	−74.6	−97.7	−77.8	−93.1

**Table 27:** MolDock docking energies (kJ/mol) of miscellaneous diterpenoids with *Leishmania donovani* and *L. mexicana* protein targets.

Cassane diterpenoids	LdonCatB	LdonCyp	LdonDHODH	LdonNMT	LmexGAPDH	LmexGPDH	LmexPGI	LmexPMM	LmexPYK	LmexPYK	LmexPYK	LmexTIM
									Site 1	Site 2	Site 3	
6β− *O*−Cinnamoyl−12−hydroxy−(13)15−en−16,12−olide−18−cassaneoic acid	−107.7	−94.7	−104.3	−108.1	−103.7	−126.5	−103.9	−136.7	−133.4	−117.8	−110.2	−91.8
6α,7β−Diacetoxyvouacapane	−83.2	−82.4	−78.8	−74.7	−77.3	−99.7	−86.4	−103.6	−102.4	−86.7	−85.6	−81.5
6β− *O*−2*'*3*'*−Dihydrocinnamoyl−12−hydroxy−(13)15−en−16,12−olide−18−cassaneoic acid	−108.6	−100.4	−52.6	−101.3	−97.7	−125.8	−92.8	−140.1	−130.8	−118.5	−109.0	−102.6
**Icetaxane diterpenoids**												
Cyclocoulterone	−89.0	−82.5	−71.2	−81.3	−78.1	−117.3	−70.7	−87.7	−98.4	−87.3	−83.5	−79.3
5− *epi*−Icetexone	−90.5	−80.3	−85.1	−27.2	−75.6	−99.1	−68.4	−89.5	−88.3	−78.6	−80.3	−80.3
Komaroviquinone	−75.5	−81.9	−77.6	−76.5	−72.6	−105.7	−73.9	−90.4	−92.8	−82.4	−81.2	−69.5
**Mulinane diterpenoids**												
Azorellanol	−80.6	−82.6	−87.2	−80.0	−71.4	−100.2	−77.7	−95.1	−100.2	−89.9	−94.0	−84.8
7−Deacetylazorellanol	−73.7	−90.1	−81.0	−72.4	−75.4	−103.4	−72.2	−91.8	−88.7	−82.8	−89.1	−72.3
Mulin−11,13−dien−20−oic acid	−84.7	−95.2	−85.0	−71.5	−71.3	−97.5	−72.5	−98.2	−99.2	−80.6	−92.2	−86.8
Mulinic acid	−63.1	−93.4	−72.3	−82.3	−70.2	−98.9	−63.7	−85.5	−94.4	−77.2	−87.1	−83.0
Mulinolic acid	−76.7	−95.1	−77.5	−85.1	−78.7	−105.7	−68.8	−94.1	−94.7	−80.4	−87.4	−82.6
**Miscellaneous diterpenoids**												
Hypoestoxide	−80.6	−92.1	−67.1	−85.2	−78.1	−94.9	−85.7	−114.8	−102.4	−79.6	−87.9	−72.7
Komarovispirone	−77.7	−80.5	−67.4	−75.5	−70.3	−101.0	−77.5	−90.5	−92.3	−86.1	−80.8	−79.0
Sacculatal	−86.4	−84.1	−84.0	−82.4	−77.2	−103.5	−78.1	−92.5	−104.3	−87.9	−98.7	−79.1
Serratol	−67.7	−78.7	−59.5	−77.4	−67.0	−89.9	−71.4	−94.3	−92.3	−82.9	−90.1	−70.1
Totarol	−63.9	−70.3	−59.4	−69.8	−60.5	−88.0	−68.6	−84.0	−81.5	−72.2	−90.4	−65.7

**Table 28:** MolDock docking energies (kJ/mol) of miscellaneous diterpenoids with *Leishmania infantum* protein targets.

Kaurane diterpenoids	LinfCYP51	LinfGLO2	LinfPnC1	LinfTDR1	LinfTR
15−Angeloyl−4α,15β−kaur−16−en−18−oic acid	−97.4	−64.6	no dock	−82.4	−95.6
*ent*−11α−Hydroxy−16−kauren−15−one	−82.8	−66.5	no dock	−66.5	−76.6
Kaurenoic acid	−79.9	−65.8	no dock	−77.4	−82.6
Perymenic acid	−98.3	−77.0	no dock	−83.2	−97.0
*ent*−15β−Senecioyloxy−16,17−epoxy−kauran−18−oic acid	−99.6	−77.9	no dock	−85.4	−102.2
**Pimarane diterpenoids**					
Acanthoic_acid	−83.6	−64.6	no dock	−72.4	−82.6
7β−Hydroxy− *ent*−pimara−8(14),15−dien−19−oic acid	−90.4	−67.2	no dock	−75.0	−79.2
*ent*−15−Pimarene−8β,19−diol	−84.0	−63.8	no dock	−69.9	−77.4
*ent*−8(14),15−Pimaradien−3β−acetoxy	−90.6	−73.3	no dock	−70.5	−92.1
*ent*−8(14),15−Pimaradien−3β,19−diol	−82.2	−69.2	no dock	−67.0	−78.9
*ent*−Pimara−8(14),15−dien−19−oic acid	−84.2	−69.0	no dock	−70.8	−80.3
*ent*−8(14),15−Pimaradien−3b−ol	−78.3	−67.2	no dock	−66.8	−79.1
**Cassane diterpenoids**					
6β− *O*−Cinnamoyl−12−hydroxy−(13)15−en−16,12−olide−18−cassaneoic acid	−125.4	−91.1	no dock	−105.3	−119.8
6α,7β−Diacetoxyvouacapane	−93.8	−77.7	no dock	−83.7	−93.9
6β− *O*−2*'*3*'*−Dihydrocinnamoyl−12−hydroxy−(13)15−en−16,12−olide−18−cassaneoic acid	−124.7	−88.7	no dock	−99.0	−114.1
**Icetaxane diterpenoids**					
Cyclocoulterone	−90.3	−68.7	no dock	−76.9	−90.0
5− *epi*−Icetexone	−99.1	−75.8	no dock	−81.0	−81.6
Komaroviquinone	−96.8	−72.4	no dock	−75.6	−88.7
**Mulinane diterpenoids**					
Azorellanol	−99.1	−72.4	no dock	−78.2	−91.2
7−Deacetylazorellanol	−88.5	−71.4	no dock	−76.5	−85.9
Mulin−11,13−dien−20−oic acid	−87.5	−72.9	no dock	−79.1	−78.6
Mulinic acid	−88.3	−70.6	no dock	−73.8	−84.3
Mulinolic acid	−89.0	−78.1	no dock	−78.6	−85.8
**Miscellaneous diterpenoids**					
Hypoestoxide	−97.2	−82.2	no dock	−73.6	−91.2
Komarovispirone	−83.1	−72.0	no dock	−74.4	−79.9
Sacculatal	−93.5	−77.7	no dock	−84.4	−88.1
Serratol	−81.7	−77.1	no dock	−78.5	−78.5
Totarol	−76.4	−67.7	no dock	−67.9	−73.4

**Figure 19:**
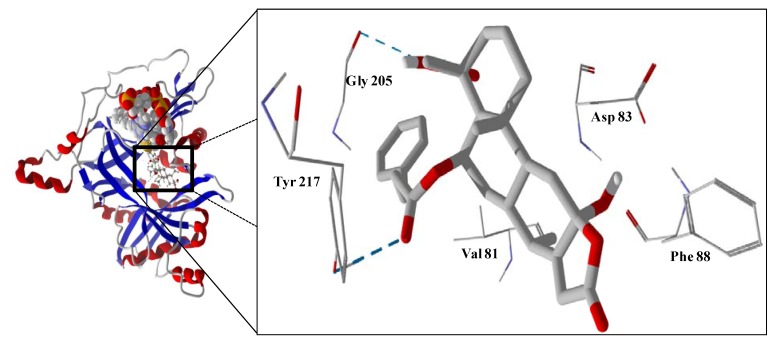
The lowest-energy pose of 6β-*O*-2*'*3*'*-dihydrocinnamoyl-12-hydroxy-(13)15-en-16,12-olide-18-cassaneoic acid and LmajNMT. The hydrogen bonding interactions between the ligand and the protein (Gly 205 and Tyr 217) is shown as blue dash lines. Val 81, Asp 83 and Phe 88 were also predicted to have very strong steric interactions with the ligand.

Over 100 antiparasitic diterpenoids were docked into the protein targets in this study. These include abietane-, clerodane-, kaurane-, labdane-, pimarane-, cassane- and mulinane-like diterpenoids. In general, diterpenoids preferentially dock to LmajMetRS, LmajDHODH and LmexGPDH. 12-*O*-deacetyl-6-*O*-acetyl-18-acetyloxycoleon Q, the antiplasmodial diterpene isolated from the tropical Asia plant *Anisochilus harmandii* (Lamiaceae) [64] has a stronger docking energy (−111.8 kJ/mol) for LmajPDE1 than the co-crystallized competitive phosphodiesterase inhibitor, 3-isobutyl-1-methylxanthine (docking energy = −78.1 kJ/mol). 14(*R*)-Aulacocarpin C and 15(*S*)-methoxy-labdan-8(17),11(*E*),13(14)-trien-15,16-olide are also selective for LmexPYK and LmajOPB, respectively. The cassane-like antileishmanial diterpenes 6β-*O*-2*'*3*'*-dihydrocinnamoyl-12-hydroxy-(13)15-en-16,12-olide-18-cassaneoic acid and 6β-*O*-cinnamoyl-12-hydroxy-(13)15-en-16,12-olide-18-cassaneoic acid have stronger docking energies than any of the other docked diterpenoids for most of the protein targets. These higher docking energies correlate with the fact they have higher molecular weights that the other diterpenoids. Despite the molecular weight correlation, 6β-*O*-2*'*3*'*-dihydrocinnamoyl-12-hydroxy-(13)15-en-16,12-olide-18-cassaneoic acid (MW: 496.592) is selective for LmajNMT. The compound’s docking score for LmajNMT is comparable to that of the protein’s co-crystallized pyrazole sulfonamide inhibitor (PDB ID: 4a30; MW: 495.425; docking energy = −121.1 kJ/mol). The lowest energy pose of 6β-*O*-2*'*3*'*-dihydrocinnamoyl-12-hydroxy-(13)15-en-16,12-olide-18-cassaneoic acid is predicted to have extensive interactions with residues Ala 204, Asp 83, Asp 84, Glu 82, Gly 205, Phe 88, Phe 90, Tyr 217, Tyr 345, Val 81 and Val 206. The cassane diterpenoid is predicted to have hydrogen bonding interactions through its carboxylic acid moiety and the ester bond of its cinnamoyl substituent to the backbone carbonyl group of Gly 205, and the side chain phenolic residue of Tyr 217, respectively (Figure 19). The icetaxane diterpenoids 5-*epi-*icetaxone showed preferential docking to *L. infantum* sterol 14α-demethylase (LinfCYP51) with a lower docking energy than the co-crystallized ligand, fluconazole (−99.1 and −91.3 kJ/mol, respectively).

### 2.4. Triterpenoid Docking

**Figure 20:**
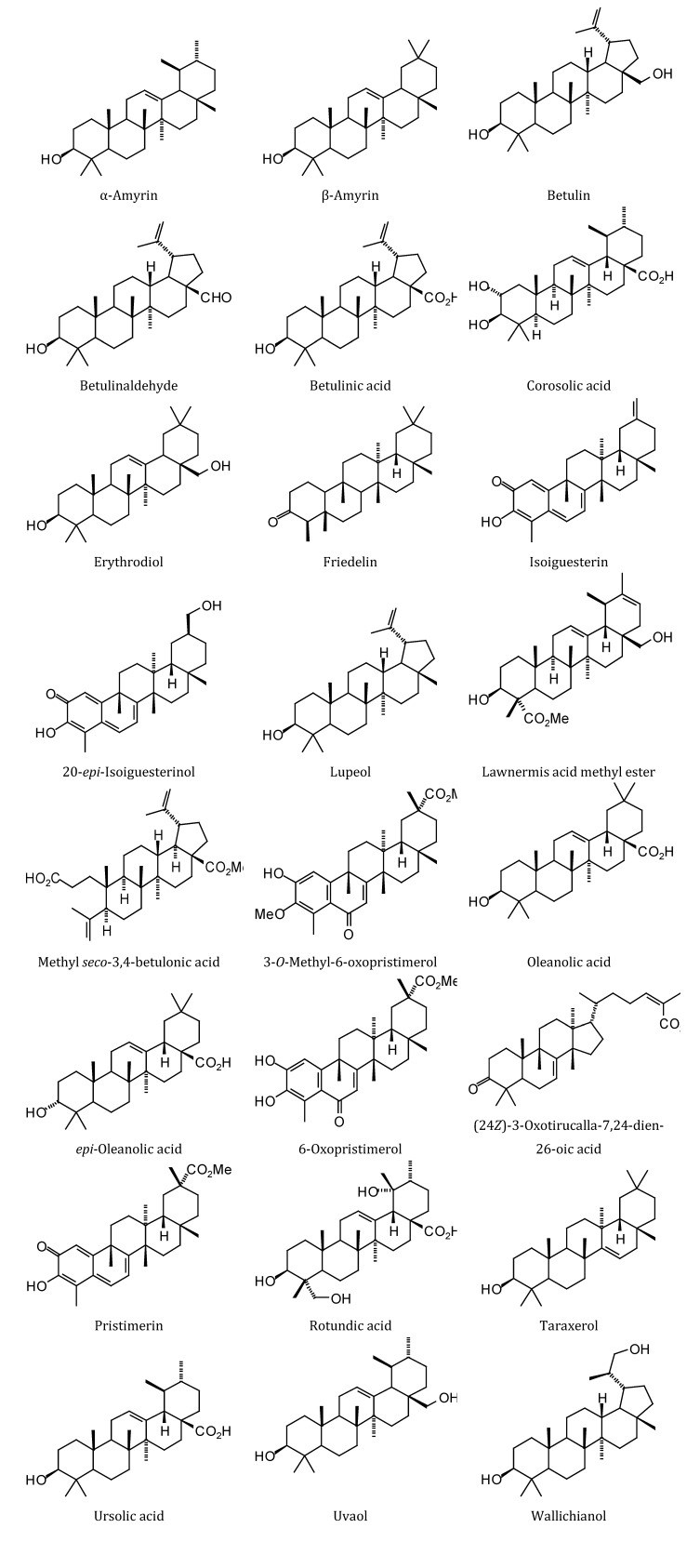
Triterpenoids examined in this work.

**Figure 21:**
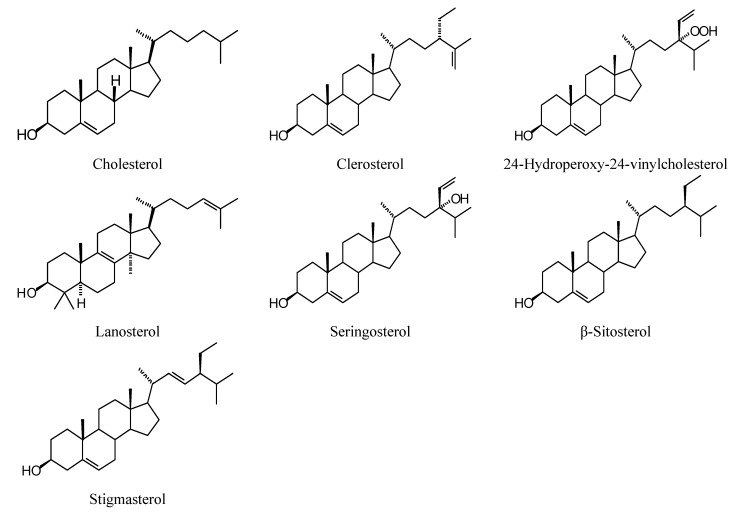
Steroids examined in this work.

**Figure 22:**
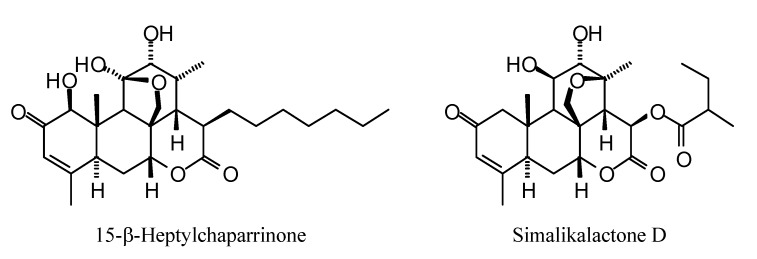
Quassinoids examined in this work.

**Figure 23:**
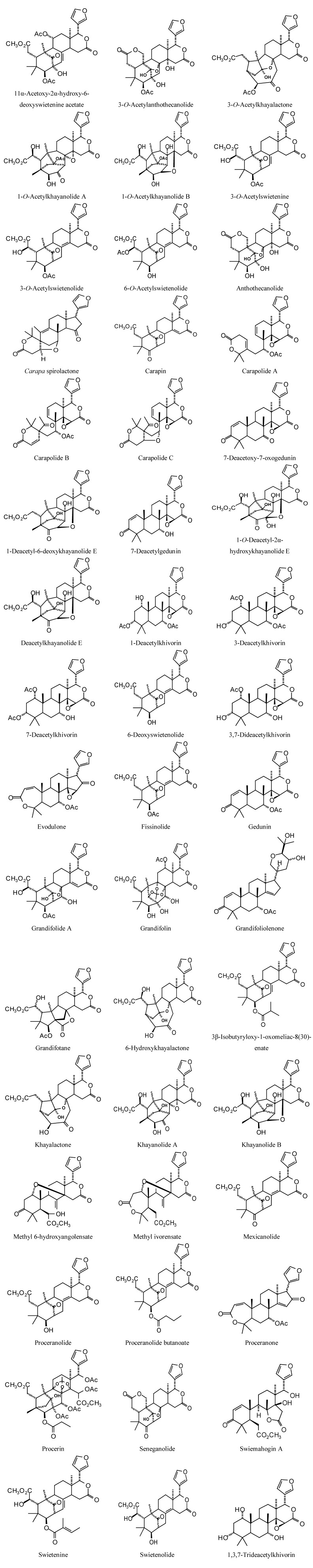
Limonoids examined in this work.

**Figure 24:**
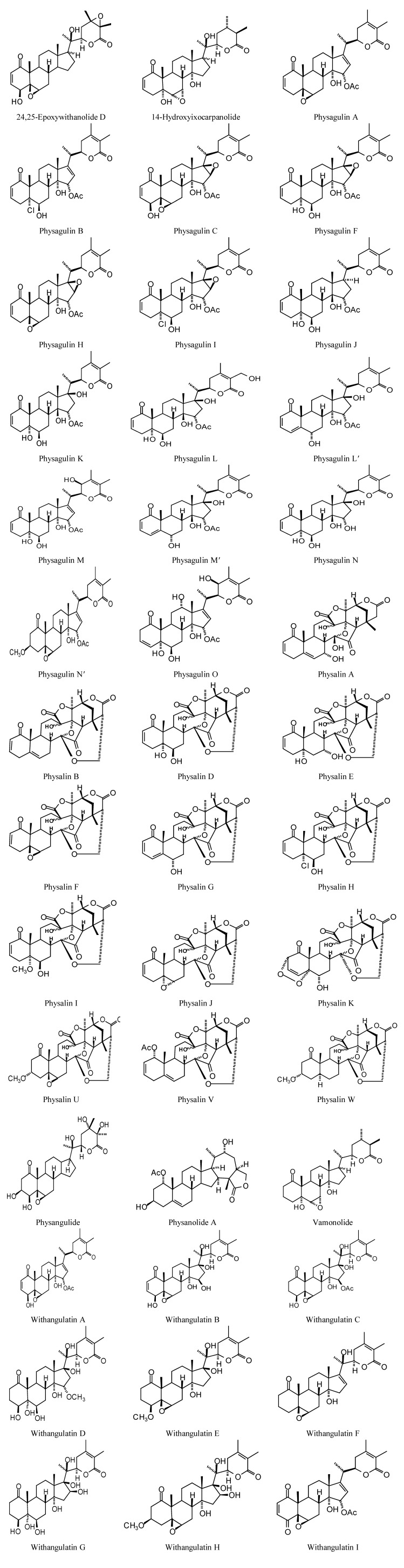
Withanolides examined in this work.

Triterpenoids, including limonoids, withanolides, quassinoids, and steroids are shown in Figure 20, Figure 21, Figure 22, Figure 23, and Figure 24. The docking energies for the triterpenoid-based ligands are compiled in Table 29, Table 30, Table 31, Table 32, Table 33, Table 34, Table 35, Table 36, Table 37, Table 38, Table 39 and Table 40. The triterpenoids ligands with the strongest docking energies were the limonoids carapolide A and khayanolide A, and the withanolides 24,25-epoxywithanolide D and withangulatin A. Carapolide A showed significant docking to LmajDHODH (docking energy = −140.0 kJ/mol). Khayanolide A preferentially docked with LmajMetRS and LmexGPDH (docking energies = −138.5 and −137.8 kJ/mol, respectively). The limonoid with the strongest docking was, however, grandifotane with LmajDHODH with a docking energy of −154.4 kJ/mol. 6-*O*-Acetylswietenolide docked strongly to LmexGPDH (docking energy = −142.7 kJ/mol). The withanolide with the strongest docking was physagulin F with LmajMetRS, which had a docking energy of −133.2 kJ/mol. As a class, the steroids showed the most target selectivity with six of the seven steroids significantly docking more strongly to LmajMetRS. In general, limonoids showed some selectivity for LmexGPDH and LmajDHODH, while withanolides docked more selectively with LmajUGPase.

**Figure 25:**
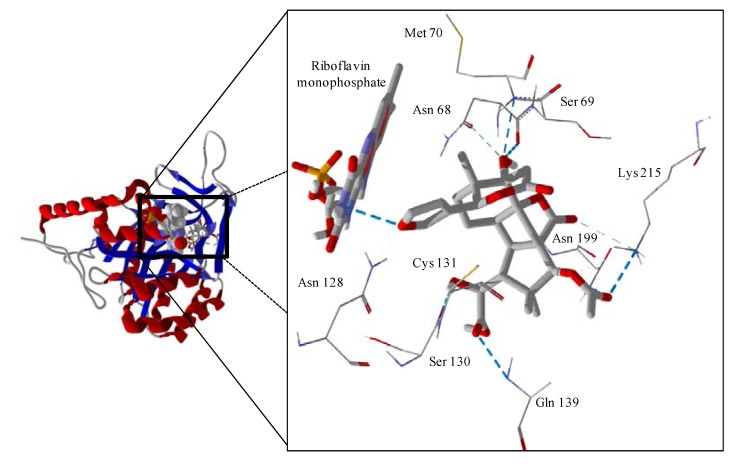
Lowest-energy docked pose of grandifontane with *L. major* dihydroorotate dehydrogenase (LmajDHODH, PDB 3mhu) showing key interactions with Cys 131, Asn 199, Asn 68, Ser 69, and Gln 139. Hydrogen-bonds are shown as blue dashed lines.

**Figure 26:**
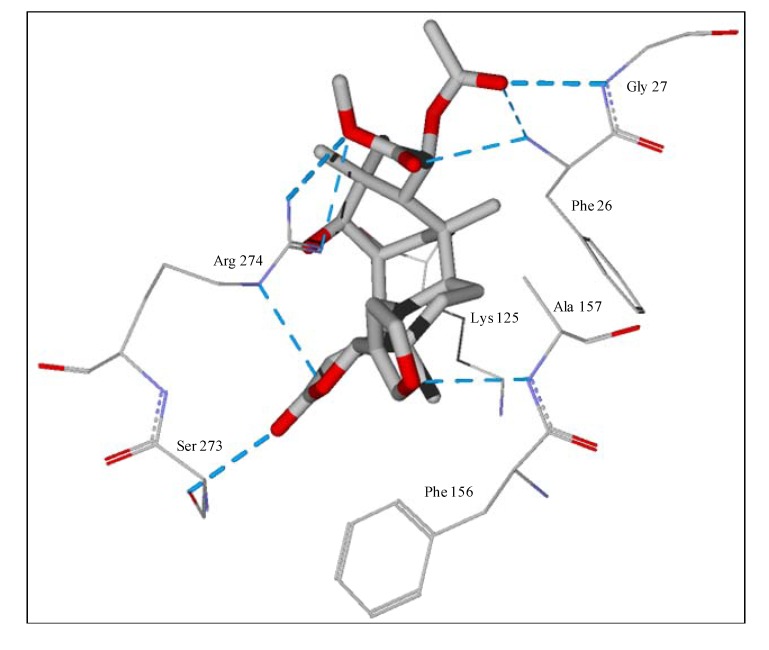
Lowest-energy docked pose of 6-*O*-acetylswietenolide with *L. mexicana* glycerol-3-phosphate dehydrogenase (LmexGPDH, PDB 1n1e) showing key interactions with Arg 274, Ser 293, Phe 26 and Ala 157, and Gln 139. Hydrogen-bonds are shown as blue dashed lines.

**Figure 27:**
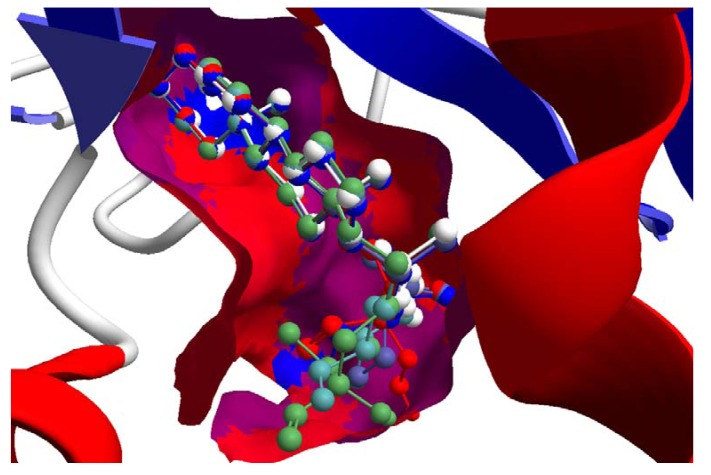
Lowest-energy poses of steroids, cholesterol (white), clerosterol (purple), saringosterol (green), stigmasterol (cyan), β-sitosterol (blue), and 24-hydroperoxy-24-vinylcholesterol (red), in the hydropobic pocket of *L. major* methionyl t-RNA synthetase (LmajMetRS, PDB 3kfl).

Grandifotane showed selective docking to LmajDHODH with a docking energy of −154.4 kJ/mol. The lowest-energy pose of the ligand placed the compound at the binding site of the co-crystallized ligand, 5-nitroorotic acid (Figure 25). The furan ring of the ligand is sandwiched between the riboflavin monophosphate cofactor and Cys 131. There are hydrogen-bonding interactions between the docked ligand and residues Asn 199, Asn 68, Ser 69, and Ser 130. Grandifotane, isolated from *Khaya grandifoliola* [65], has apparently not been reported to be antiparasitic. The bark and seeds of *K. grandifoliola*, however, have shown significant antimalarial activity [66]. 6-*O*-Acetylswietenolide, also isolated from *K. grandifoliola*, has shown antiplasmodial activity [67], and this compound showed preferential docking to LmexGPDH. The lowest-energy docking pose of 6-*O*-acetylswietenolide with LmexGPDH (Figure 26) lies in a cavity surrounded by Arg 274, Phe 26, Lys 125, Phe 156, Val 298, Lys 210, and Ala 157. Lys 125 and Lys 210 have been identified as critical to catalytic activity of this enzyme [15]. With the exception of lanosterol, the steroid ligands showed preferential docking to LmajMetRS. The lowest-energy poses for these steroids show them all occupying the methionyl adenylate binding site (Figure 27). The tetracyclic steroidal structures all occupy the same position in a hydrophobic pocket surrounded by Asp 486, Trp 515, Lys 522, His 228, Gly 227, His 513, and Tyr 218, and hydrogen-bonded by way of the 3-hydroxyl group of the steroid to Trp 516 and Gly 514. Clerosterol has shown *in vitro* antileishmanial activity [68], while saringosterol, stigmasterol, and 24-hydroperoxy-24-vinylcholesterol, in addition to clerosterol, have shown *in vitro* antitrypanosomal activity [69]. β-Sitosterol has shown modest antitrypanosomal activity [70].

**Table 29:** MolDock docking energies (kJ/mol) of limonoids with *Leishmania major* protein targets.

Limonoids	LmajCatB	LmajDHODH	LmajdUTPase	LmajNDKb	LmajNH	LmajNMT	LmajOPB	LmajPDE1	LmajPTR1	LmajMetRS	LmajTyrRS	LmajUGPase
11α−Acetoxy−2α−hydroxy−6−deoxyswietenine_acetate	−77.4	−100.5	−112.6	−84.0	−112.9	−113.7	−79.4	−90.7	−99.9	−85.3	−106.7	−103.7
3− *O*−Acetylanthothecanolide	−102.6	−129.9	−114.6	−91.2	−111.4	−121.1	−103.5	−96.4	−102.1	−103.7	−106.8	−107.6
3− *O−*Acetylkhayalactone	−70.4	−103.4	−96.4	−98.9	−115.9	−123.4	−96.9	−109.5	−109.5	−114.5	−106.6	−123.7
1− *O*−Acetylkhayanolide A	−87.7	−103.2	−85.4	−86.3	−96.5	−114.1	−92.2	−93.2	−88.7	−81.1	−105.0	−106.8
1− *O*−Acetylkhayanolide B	−80.0	−86.6	−87.0	−87.1	−80.7	−105.0	−105.5	−74.5	−72.8	−91.8	−95.5	−94.5
3− *O*−Acetylswietenine	−79.2	−90.6	−95.8	−69.9	−102.9	−104.3	−82.6	−84.6	−82.4	−92.3	−95.5	−121.8
3− *O*−Acetylswietenolide	−91.9	−115.0	−92.7	−82.9	−98.6	−115.0	−74.1	−81.5	−86.8	−80.1	−93.6	−100.2
6− *O−*Acetylswietenolide	−87.9	−107.6	−80.0	−86.6	−97.4	−102.0	−81.6	−98.4	−85.7	−96.3	−107.5	−108.7
Anthothecanolide	−103.7	−133.3	−85.5	−101.6	−93.7	−112.3	−98.4	−94.8	−104.4	−97.4	−96.8	−114.1
Carapa spirolactone	−92.7	−91.4	−87.0	−89.7	−99.1	−90.6	−93.9	−104.0	−71.8	−92.1	−95.7	−86.5
Carapin	−79.9	−109.2	−89.9	−95.0	−92.8	−95.0	−84.8	−92.6	−86.4	−88.3	−98.9	−110.5
Carapolide A	−107.8	−140.0	−101.1	−114.9	−111.7	−119.2	−98.5	−113.5	−92.4	−125.5	−107.1	−123.5
Carapolide B	−104.9	−128.3	−88.1	−117.3	−111.7	−117.4	−124.7	−106.0	−82.3	−98.6	−107.9	−107.4
Carapolide C	−94.5	−105.1	−82.7	−106.2	−99.2	−110.1	−118.5	−109.8	−76.3	−95.5	−104.4	−107.8
7−Deacetoxy−7−oxogedunin	−51.6	−95.8	−82.2	−90.1	−86.0	−93.6	−100.1	−100.2	−91.2	−79.0	−91.3	−86.5
1− *O*−Deacetyl−6−deoxykhayanolide E	−104.2	−81.4	−75.8	−79.7	−96.9	−94.3	−88.0	−72.9	−85.6	−96.6	−94.1	−102.8
7−Deacetylgedunin	−84.7	−86.9	−78.5	−84.8	−85.2	−87.5	−96.9	−99.7	−89.9	−83.2	−91.5	−90.3
1− *O*−Deacetyl−2α−hydroxykhayanolide E	−96.4	−105.3	−77.1	−74.1	−91.7	−101.1	−93.7	−81.9	−85.9	−97.6	−109.8	−108.7
Deacetylkhayanolide E	−104.7	−85.6	−76.5	−80.6	−88.0	−97.2	−87.0	−72.6	−83.1	−98.8	−110.8	−102.9
1−Deacetylkhivorin	−60.5	−92.7	−96.1	−78.3	−96.3	−107.5	−58.1	−84.0	−81.8	−97.0	−98.5	−97.7
3−Deacetylkhivorin	−73.2	−95.7	−90.4	−71.8	−96.0	−110.6	−99.1	−99.4	−81.4	−82.4	−98.7	−108.2
7−Deacetylkhivorin	−77.8	−94.0	−99.2	−85.0	−88.2	−96.3	−93.5	−79.1	−87.1	−83.7	−89.9	−95.6
6−Deoxyswietenolide	−84.5	−100.2	−79.3	−84.0	−97.3	−108.8	−90.1	−84.6	−93.8	−90.9	−98.2	−100.5
3,7−Dideacetylkhivorin	−81.5	−93.0	−83.4	−64.8	−105.3	−96.3	−93.9	−109.3	−108.1	−76.6	−104.4	−99.6
Evodulone	−83.0	−113.4	−87.5	−83.7	−103.1	−103.3	−96.8	−114.6	−87.5	−80.9	−96.0	−101.9
Fissinolide	−89.4	−112.8	−93.5	−80.4	−99.0	−121.6	−99.6	−85.5	−94.3	−110.6	−88.8	−97.1
Gedunin	−74.5	−106.8	−81.4	−70.2	−89.5	−102.1	−93.5	−102.8	−96.4	−87.1	−93.1	−97.7
Grandifolide A	−75.5	−113.8	−93.3	−101.7	−115.1	−128.4	−96.9	−94.9	−95.7	−98.4	−108.3	−102.9
Grandifolin	−91.5	−110.0	−99.7	−92.9	−96.2	−100.3	−94.8	−94.1	−114.7	−95.8	−108.9	−100.0
Grandifoliolenone	−84.6	−88.5	−89.0	−88.7	−103.9	−106.0	−102.8	−97.8	−89.6	−86.1	−94.2	−119.8
Grandifotane	−102.5	−154.5	−94.6	−80.1	−99.5	−106.1	−90.4	−107.9	−96.4	−96.5	−104.0	−112.7
6−Hydroxykhayalactone	−112.4	−105.3	−89.5	−111.0	−106.7	−116.6	−92.8	−88.3	−98.9	−101.2	−111.5	−126.4
3β−Isobutyryloxy−1−oxomeliac−8(30)−enate	−95.6	−119.9	−109.6	−89.9	−101.4	−111.4	−105.5	−81.3	−98.8	−86.9	−109.1	−110.0
Khayalactone	−107.6	−110.0	−90.3	−103.4	−102.6	−115.8	−92.1	−96.1	−104.7	−101.0	−113.2	−126.4
Khayanolide A	−110.8	−123.5	−101.3	−104.4	−111.1	−115.5	−98.4	−105.9	−107.9	−138.5	−127.4	−123.9
Khayanolide B	−104.1	−93.6	−77.9	−86.4	−93.6	−99.7	−94.8	−59.4	−86.1	−97.9	−99.8	−105.4
Khivorin	−75.6	−98.7	−99.2	−91.1	−99.1	−101.7	−97.5	−77.8	−92.1	−89.3	−101.3	−109.6
Methyl acetoxyangolensate	−90.2	−102.1	−75.7	−99.5	−102.2	−107.2	−82.9	−93.5	−80.3	−108.2	−98.2	−94.5
Methyl angolensate	−89.2	−104.2	−82.9	−100.1	−92.6	−109.9	−85.1	−90.8	−79.7	−110.5	−113.2	−108.6
Methyl hydroxyangolensate	−82.9	−91.4	−73.0	−102.8	−87.6	−109.2	−73.0	−84.1	−79.5	−100.2	−108.8	−101.4
Methyl ivorensate	−79.5	−106.7	−95.7	−102.4	−98.9	−99.8	−84.7	−76.1	−87.2	−95.8	−106.5	−108.2
Mexicanolide	−86.9	−103.0	−82.7	−79.6	−95.4	−111.6	−90.8	−90.2	−95.6	−100.5	−93.9	−103.4
Proceranolide	−86.5	−110.3	−77.8	−96.1	−97.0	−108.7	−76.0	−91.5	−94.3	−91.7	−102.2	−102.0
Proceranolide butanoate	−94.0	−119.7	−87.9	−93.8	−97.5	−119.2	−95.9	−103.9	−89.2	−90.9	−107.1	−106.6
Proceranone	−84.4	−103.0	−93.9	−97.8	−101.8	−106.0	−88.2	−115.6	−91.4	−82.7	−89.5	−113.2
Procerin	−71.9	−98.4	−75.8	−19.8	−76.4	−96.8	−67.9	−46.1	−70.5	−58.8	−88.9	−78.6
Seneganolide	−101.7	−122.0	−94.5	−106.6	−96.4	−110.3	−92.6	−92.4	−104.9	−106.9	−105.8	−104.6
Swiemahogin A	−92.4	−125.6	−91.5	−115.5	−116.8	−119.1	−103.7	−122.0	−81.1	−111.1	−106.4	−123.7
Swietenine	−93.7	−114.2	−99.4	−84.6	−113.1	−107.1	−88.8	−87.7	−87.6	−91.1	−106.8	−110.9
Swietenolide	−85.7	−108.1	−80.4	−83.7	−97.4	−109.0	−71.8	−82.8	−84.3	−102.3	−97.4	−98.9
1,3,7−Trideacetylkhivorin	−86.0	−95.7	−79.2	−76.7	−94.8	−88.5	−101.1	−97.0	−61.9	−81.2	−97.1	−84.1

**Table 30:** MolDock docking energies (kJ/mol) of limonoids with *Leishmania donovani* and *L. mexicana* protein targets.

Limonoids	LdonCatB	LdonCyp	LdonDHODH	LdonNMT	LmexGAPDH	LmexGPDH	LmexPGI	LmexPMM	LmexPYK	LmexPYK	LmexPYK	LmexTIM
									Site 1	Site 2	Site 3	
11α−Acetoxy−2α−hydroxy−6−deoxyswietenine acetate	−92.2	−85.4	−46.3	−82.9	−94.0	−99.6	−88.0	−121.3	−95.5	−103.8	−103.9	−81.9
3−*O*−Acetylanthothecanolide	−100.8	−85.9	−54.7	−70.9	−108.1	−100.9	−94.3	−117.9	−110.2	−97.0	−94.7	−97.5
3−*O−*Acetylkhayalactone	−77.2	−66.9	−90.3	−100.7	−108.8	−113.1	−104.3	−110.2	−104.4	−111.4	−107.4	−107.8
1−*O*−Acetylkhayanolide A	−80.2	−47.6	−38.3	−92.4	−96.6	−115.5	−103.5	−110.9	−116.4	−101.2	−90.2	−84.7
1−*O*−Acetylkhayanolide B	−81.3	−68.4	−65.9	−72.5	−83.2	−106.6	−82.6	−88.0	−94.9	−84.9	−91.4	−82.7
3−*O*−Acetylswietenine	−75.2	−78.2	−57.4	−106.0	−74.8	−118.6	−81.1	−106.8	−101.8	−108.3	−103.5	−61.4
3−*O*−Acetylswietenolide	−88.0	−81.2	−37.9	−106.8	−72.1	−103.8	−83.5	−89.0	−98.3	−95.5	−96.0	−68.2
6−*O−*Acetylswietenolide	−86.6	−69.6	−58.4	−91.1	−82.4	−142.7	−92.8	−85.9	−110.1	−97.9	−95.8	−67.7
Anthothecanolide	−97.5	−66.6	−69.0	−92.0	−95.0	−99.1	−86.8	−118.1	−100.7	−98.3	−91.0	−91.5
Carapa spirolactone	−89.9	−75.5	−65.2	−61.7	−88.6	−94.8	−78.3	−96.5	−91.4	−94.8	−85.9	−78.6
Carapin	−71.7	−102.8	no dock	−94.8	−84.6	−106.0	−78.4	−99.7	−104.6	−94.8	−82.9	−78.9
Carapolide A	−113.0	−101.2	−88.5	−97.5	−88.6	−118.9	−94.1	−112.6	−114.2	−115.6	−106.5	−100.6
Carapolide B	−96.9	−82.5	−21.9	−93.1	−90.7	−107.7	−90.1	−98.7	−107.0	−109.9	−99.4	−91.4
Carapolide C	−102.9	−99.2	−58.0	−78.9	−83.1	−112.1	−88.5	−115.3	−107.4	−113.3	−98.4	−94.4
7−Deacetoxy−7−oxogedunin	−68.4	−66.9	−14.8	−79.7	−79.6	−92.1	−73.6	−83.5	−111.5	−101.0	−95.5	−83.9
1−*O*−Deacetyl−6−deoxykhayanolide E	−101.8	−87.2	−70.1	−73.5	−82.3	−99.9	−82.4	−75.6	−97.4	−99.5	−91.7	−71.8
7−Deacetylgedunin	−52.7	−81.4	−11.3	−82.6	−84.8	−91.5	−74.1	−82.5	−98.3	−93.9	−93.3	−80.6
1−*O*−Deacetyl−2α−hydroxykhayanolide E	−98.1	−58.4	−48.5	−64.2	−79.0	−111.8	−87.0	−84.9	−100.4	−104.5	−86.2	−66.2
Deacetylkhayanolide E	−96.2	−69.1	−56.0	−58.8	−84.2	−92.6	−85.0	−77.8	−97.5	−101.8	−88.6	−64.2
1−Deacetylkhivorin	−52.5	−89.2	no dock	−74.9	−76.6	−99.4	−83.8	−84.7	−98.4	−106.4	−93.5	−66.4
3−Deacetylkhivorin	−68.8	−92.2	−66.2	−84.2	−96.9	−100.5	−99.7	−85.4	−98.4	−91.3	−79.4	−71.6
7−Deacetylkhivorin	−68.1	−68.6	−36.1	−94.8	−88.0	−99.8	−83.1	−85.5	−105.3	−104.6	−94.1	−75.9
6−Deoxyswietenolide	−90.3	−75.0	no dock	−94.3	−74.2	−114.6	−84.8	−105.7	−90.4	−86.9	−94.5	−73.0
3,7−Dideacetylkhivorin	−62.6	−69.3	−63.2	−87.3	−92.3	−96.2	−88.0	−78.6	−113.5	−85.2	−98.5	−73.1
Evodulone	−80.2	−84.8	−74.3	−90.6	−79.0	−98.7	−86.6	−103.2	−103.4	−105.3	−93.2	−88.4
Fissinolide	−83.6	−80.3	−54.6	−102.4	−64.2	−95.1	−85.1	−111.9	−90.4	−95.2	−92.9	−78.1
Gedunin	−84.0	−88.2	no dock	−88.3	−85.0	−98.3	−80.4	−82.0	−88.4	−99.1	−95.6	−88.9
Grandifolide A	−77.1	−96.3	no dock	−98.1	−91.2	−103.8	−90.9	−93.4	−104.5	−115.5	−87.3	−85.8
Grandifolin	−53.7	−94.9	−67.0	−73.2	−83.7	−109.1	−89.4	−95.4	−105.1	−98.9	−104.0	−99.3
Grandifoliolenone	−93.7	−93.8	−68.9	−77.3	−78.7	−115.1	−80.0	−101.4	−115.4	−103.6	−100.1	−87.3
Grandifotane	−99.7	−70.8	−40.6	−99.4	−107.8	−113.7	−87.5	−117.2	−100.5	−100.6	−106.8	−29.1
6−Hydroxykhayalactone	−106.7	−80.2	−88.2	−96.4	−94.0	−106.1	−97.3	−98.6	−103.4	−110.1	−91.2	−95.9
3β−Isobutyryloxy−1−oxomeliac−8(30)−enate	−92.4	−82.3	no dock	−99.8	−87.6	−99.2	−87.8	−89.7	−95.3	−117.6	−92.1	−60.0
Khayalactone	−107.0	−68.1	−55.1	−97.7	−94.6	−116.0	−95.9	−102.0	−100.2	−100.8	−95.6	−94.2
Khayanolide A	−110.9	−98.7	−18.3	−97.8	−92.5	−137.8	−98.6	−112.1	−115.3	−107.8	−107.3	−89.9
Khayanolide B	−102.5	−72.5	−39.5	−70.4	−90.8	−94.6	−88.3	−77.6	−88.2	−105.2	−94.7	−71.2
Khivorin	−64.6	−89.4	−72.0	−30.2	−92.4	−107.4	−82.0	−84.0	−93.9	−108.2	−85.6	−78.8
Methyl acetoxyangolensate	−89.1	−58.8	−59.5	−88.8	−98.0	−94.6	−72.7	−81.8	−108.9	−98.4	−94.8	−41.3
Methyl angolensate	−89.5	−52.0	−74.5	−27.42	−98.6	−112.9	−75.3	−109.7	−99.1	−95.9	−87.2	−79.6
Methyl hydroxyangolensate	−91.0	−73.9	−74.7	−75.6	−94.5	−115.7	−76.6	−112.5	−93.1	−93.7	−87.6	−78.9
Methyl ivorensate	−86.9	−75.4	−63.9	−82.2	−102.0	−119.7	−77.1	−80.8	−104.0	−91.3	−96.2	−70.8
Mexicanolide	−85.4	−67.6	−33.9	−84.3	−67.9	−112.8	−92.2	−105.5	−97.3	−90.4	−98.3	−59.4
Proceranolide	−90.2	−77.6	no dock	−94.7	−66.8	−121.6	−87.2	−106.0	−90.0	−88.8	−94.6	−73.9
Proceranolide butanoate	−94.7	−81.0	−70.3	−111.8	−76.2	−92.9	−89.1	−95.1	−100.9	−106.8	−107.4	−63.0
Proceranone	−87.7	−89.0	no dock	−84.6	−75.0	−99.3	−86.0	−109.9	−98.0	−108.4	−87.7	−98.6
Procerin	−77.5	−22.4	−59.7	−107.5	−94.8	−111.3	−108.8	−84.5	−93.9	−86.6	−70.7	−64.8
Seneganolide	−99.5	−92.0	−51.9	−88.4	−89.9	−98.8	−86.6	−109.8	−95.9	−92.5	−92.5	−83.5
Swiemahogin A	−69.7	−94.9	−52.2	−81.4	−79.9	−125.4	−92.8	−117.4	−120.1	−106.2	−110.0	−91.4
Swietenine	−90.7	−67.4	no dock	−99.3	−85.0	−104.1	−86.3	−91.6	−99.6	−122.3	−100.7	−78.0
Swietenolide	−87.3	−72.3	no dock	−93.6	−72.3	−121.0	−88.9	−94.4	−99.2	−91.2	−87.7	−68.3
1,3,7−Trideacetylkhivorin	−53.7	−80.5	no dock	−76.2	−87.1	−97.2	−81.2	−71.5	−96.9	−93.0	−82.8	−83.2

**Table 31:** MolDock docking energies (kJ/mol) of limonoids with *Leishmania infantum* protein targets.

Limonoids	LinfCYP51	LinfGLO2	LinfPnC1	LinfTDR1	LinfTR
11α−Acetoxy−2α−hydroxy−6−deoxyswietenine acetate	−98.0	−75.1	no dock	−93.6	−89.4
3−*O*−Acetylanthothecanolide	−102.5	−77.7	no dock	−82.5	−101.6
3−*O−*Acetylkhayalactone	−116.2	−99.5	no dock	−103.7	−113.2
1−*O*−Acetylkhayanolide A	−102.4	−89.1	no dock	−87.9	−108.3
1−*O*−Acetylkhayanolide B	−114.3	−82.0	no dock	−80.6	−84.0
3−*O*−Acetylswietenine	−95.3	−74.9	no dock	−76.4	−84.2
3−*O*−Acetylswietenolide	−105.4	−80.3	no dock	−79.5	−95.4
6−*O−*Acetylswietenolide	−103.1	−77.4	no dock	−91.1	−92.6
Anthothecanolide	−107.9	−74.6	−57.2	−82.9	−94.3
Carapa spirolactone	−108.8	−69.7	no dock	−68.3	−87.3
Carapin	−94.3	−80.2	no dock	−80.9	−86.3
Carapolide A	−111.8	−89.1	no dock	−98.1	−98.3
Carapolide B	−103.1	−71.6	no dock	−91.8	−92.9
Carapolide C	−106.9	−80.4	no dock	−79.4	−92.5
7−Deacetoxy−7−oxogedunin	−104.3	−71.9	no dock	−70.0	−84.6
1− *O*−Deacetyl−6−deoxykhayanolide E	−98.0	−77.3	no dock	−78.6	−90.9
7−Deacetylgedunin	−91.2	−72.8	no dock	−72.6	−80.4
1− *O*−Deacetyl−2α−hydroxykhayanolide E	−110.6	−75.1	no dock	−83.8	−88.6
Deacetylkhayanolide E	−103.4	−81.0	−45.4	−83.0	−84.9
1−Deacetylkhivorin	−101.2	−72.0	no dock	−75.8	−87.7
3−Deacetylkhivorin	−106.0	−76.8	no dock	−76.0	−98.3
7−Deacetylkhivorin	−94.5	−83.8	no dock	−87.0	−73.7
6−Deoxyswietenolide	−95.9	−76.5	no dock	−83.0	−92.4
3,7−Dideacetylkhivorin	−95.9	−74.8	no dock	−76.3	−103.3
Evodulone	−105.3	−81.6	no dock	−88.6	−87.5
Fissinolide	−99.2	−78.0	no dock	−78.8	−99.0
Gedunin	−107.4	−75.9	no dock	−82.2	−78.4
Grandifolide A	−109.3	−89.6	no dock	−90.2	−99.8
Grandifolin	−105.8	−76.1	no dock	−75.8	−96.8
Grandifoliolenone	−98.7	−90.7	−43.1	−75.9	−92.7
Grandifotane	−111.5	−80.8	no dock	−89.8	−90.0
6−Hydroxykhayalactone	−116.0	−83.1	no dock	−100.2	−98.6
3β−Isobutyryloxy−1−oxomeliac−8(30)−enate	−102.7	−78.4	no dock	−82.5	−103.4
Khayalactone	−101.0	−90.6	no dock	−100.3	−97.5
Khayanolide A	−104.5	−83.4	no dock	−84.3	−97.4
Khayanolide B	−110.0	−77.4	−44.1	−97.2	−103.1
Khivorin	−100.9	−71.4	no dock	−82.0	−93.8
Methyl acetoxyangolensate	−99.6	−66.4	no dock	−84.9	−82.8
Methyl angolensate	−90.5	−71.0	no dock	−77.8	−85.6
Methyl hydroxyangolensate	−95.8	−65.2	−48.5	−78.2	−85.4
Methyl ivorensate	−95.5	−77.8	no dock	−85.2	−92.5
Mexicanolide	−90.4	−78.8	−27.5	−75.8	−92.3
Proceranolide	−95.7	−78.9	no dock	−83.1	−91.5
Proceranolide butanoate	−100.6	−82.1	no dock	−89.5	−93.5
Proceranone	−107.9	−89.7	no dock	−90.7	−94.6
Procerin	−94.9	−70.1	no dock	−68.8	−81.8
Seneganolide	−101.8	−76.9	no dock	−82.0	−86.4
Swiemahogin A	−113.3	−84.7	no dock	−93.4	−97.4
Swietenine	−108.3	−79.6	no dock	−83.2	−87.1
Swietenolide	−97.0	−72.7	no dock	−86.3	−93.3
1,3,7−Trideacetylkhivorin	−92.0	−73.6	no dock	−62.4	−86.6

**Table 32:** MolDock docking energies (kJ/mol) of withanolides with *Leishmania major* protein targets.

Withanolides	LmajCatB	LmajDHODH	LmajdUTPase	LmajNDKb	LmajNH	LmajNMT	LmajOPB	LmajPDE1	LmajPTR1	LmajMetRS	LmajTyrRS	LmajUGPase
24,25−Epoxywithanolide D	−92.9	−121.0	−109.3	−104.7	−104.2	−124.1	−102.7	−114.1	−114.2	−98.2	−119.5	−108.2
14−Hydroxyixocarpanolide	−77.9	−104.9	−89.2	−77.9	−100.9	−98.2	−90.0	−113.0	−81.8	−99.0	−99.6	−106.6
Physagulin A	−102.7	−123.7	−98.3	−103.1	−108.2	−113.7	−100.6	−115.8	−110.0	−98.4	−104.0	−110.6
Physagulin B	−97.0	−107.6	−89.8	−105.1	−104.5	−119.6	−95.6	−103.5	−93.8	−114.8	−106.0	−111.0
Physagulin C	−93.4	−99.5	−89.6	−105.5	−106.5	−113.8	−95.9	−118.3	−93.8	−127.3	−105.0	−122.7
Physagulin F	−92.8	−88.2	−88.0	−96.5	−106.1	−102.6	−96.9	−107.5	−90.9	−133.2	−98.8	−98.5
Physagulin H	−91.8	−112.0	−88.3	−92.9	−105.4	−111.2	−103.2	−111.8	−95.5	−126.1	−104.1	−112.8
Physagulin I	−94.2	−107.1	−92.3	−99.1	−107.6	−103.0	−97.3	−110.6	−92.1	−123.8	−103.8	−106.2
Physagulin J	−96.8	−105.4	−87.2	−90.6	−96.5	−106.7	−94.5	−117.6	−91.2	−105.6	−100.1	−110.1
Physagulin K	−97.5	−118.5	−81.7	−69.4	−111.2	−112.6	−108.9	−108.9	−95.5	−101.7	−97.3	−104.1
Physagulin L	−98.8	−114.8	−88.0	−69.9	−111.2	−112.1	−110.3	−97.3	−106.0	−103.0	−99.2	−107.6
Physagulin L*'*	−107.0	−109.0	−104.2	−99.2	−99.7	−114.2	−108.2	−111.9	−108.2	−115.0	−103.5	−101.3
Physagulin M	−72.5	−95.0	−90.9	−83.2	−104.8	−110.0	−88.3	−95.8	−89.1	−92.9	−103.3	−115.4
Physagulin M*'*	−88.5	−115.0	−93.6	−99.3	−98.2	−112.8	−92.4	−106.9	−97.1	−120.2	−100.5	−101.0
Physagulin N	−93.1	−102.8	−92.3	−93.1	−101.9	−115.2	−94.7	−108.5	−84.4	−110.6	−106.6	−118.1
Physagulin N*'*	−94.4	−102.5	−72.7	−94.6	−99.7	−99.0	−96.1	−116.1	−90.0	−110.4	−90.2	−91.9
Physagulin O	−87.3	−94.3	−81.5	−91.4	−100.8	−103.6	−95.0	−98.0	−87.0	−88.9	−95.5	−108.1
Physalin A	−86.5	−92.6	−71.2	−86.8	−87.3	−92.0	−110.7	−70.2	−75.0	−84.2	−90.0	−101.2
Physalin B	−76.9	−92.3	−80.8	−62.9	−90.0	−100.2	−86.9	−95.8	−76.7	−78.1	−88.3	−101.7
Physalin D	−83.9	−87.6	−85.1	−65.4	−101.7	−99.7	−86.9	−85.4	−68.4	−83.2	−83.8	−96.5
Physalin E	−82.1	−91.5	−85.3	−65.5	−88.5	−100.6	−94.1	−76.3	−66.7	−83.2	−82.2	−99.1
Physalin F	−81.2	−90.1	−80.7	−66.0	−93.0	−101.4	−83.5	−96.3	−73.2	−78.0	−96.7	−101.7
Physalin G	−95.6	−103.9	−80.1	−82.3	−96.3	−89.6	−91.7	−91.6	−88.0	−89.8	−103.5	−103.8
Physalin H	−82.8	−89.5	−80.4	−65.0	−98.4	−95.8	−86.2	−86.6	−67.2	−80.8	−87.1	−93.0
Physalin I	−78.8	−85.4	−82.6	−71.4	−85.9	−93.0	−87.3	−75.2	−60.5	−79.5	−82.5	−98.4
Physalin J	−80.2	−91.0	−85.1	−74.9	−96.4	−103.1	−86.6	−91.8	−73.9	−77.7	−84.1	−94.1
Physalin K	−83.1	−106.9	−73.7	−81.7	−87.5	−88.7	−66.7	−68.5	−71.5	−77.6	−81.3	−105.3
Physalin U	−81.2	−96.3	−83.0	−50.7	−93.4	−93.0	−90.4	−99.2	−74.5	−85.2	−91.9	−103.1
Physalin V	−88.4	−101.1	−83.0	−83.5	−103.5	−100.9	−96.4	−95.5	−79.4	−84.8	−86.9	−107.5
Physalin W	−93.4	−95.0	−85.1	−75.9	−91.0	−92.0	−78.6	−100.4	−80.2	−84.0	−91.7	−110.3
Physangulide	−101.9	−88.9	−104.0	−97.8	−101.8	−109.1	−92.9	−117.4	−110.6	−104.4	−96.4	−101.8
Physanolide A	−85.7	−102.9	−84.6	−69.0	−97.6	−107.9	−93.8	−103.5	−100.5	−94.5	−88.4	−101.1
Vamonolide	−83.4	−96.7	−87.8	−109.9	−95.7	−103.8	−96.5	−109.0	−84.4	−92.2	−106.7	−97.4
Withangulatin A	−92.4	−121.9	−97.8	−108.1	−110.6	−124.2	−97.1	−112.9	−110.1	−99.4	−105.1	−112.2
Withangulatin B	−105.4	−89.8	−96.1	−96.6	−95.7	−99.9	−94.3	−107.2	−96.2	−117.9	−104.7	−91.0
Withangulatin C	−85.7	−93.6	−86.6	−100.7	−90.1	−109.7	−106.3	−89.2	−94.1	−125.9	−104.6	−98.5
Withangulatin D	−88.8	−80.3	−89.4	−102.6	−96.6	−107.2	−92.0	−113.3	−86.9	−126.2	−105.2	−104.8
Withangulatin E	−57.3	−94.8	−36.3	−92.5	−85.7	−102.4	−89.1	−95.7	−88.9	−96.0	−112.7	−97.9
Withangulatin F	−85.1	−96.0	−83.8	−103.9	−97.7	−98.9	−87.9	−120.8	−85.8	−96.0	−102.4	−96.9
Withangulatin G	−91.5	−122.3	−76.0	−100.4	−95.2	−100.1	−93.7	−99.7	−87.6	−97.7	−105.8	−92.5
Withangulatin H	−91.6	−105.5	−89.1	−96.8	−81.0	−94.8	−105.1	−88.8	−71.0	−115.3	−102.2	−106.9
Withangulatin I	−98.8	−112.4	−90.0	−91.3	−106.6	−113.1	−88.1	−105.6	−106.0	−98.2	−102.3	−110.7

**Table 33:** MolDock docking energies (kJ/mol) of withanolides with *Leishmania donovani* and *L. mexicana* protein targets.

	LdonCatB	LdonCyp	LdonDHODH	LdonNMT	LmexGAPDH	LmexGPDH	LmexPGI	LmexPMM	LmexPYK	LmexPYK	LmexPYK	LmexTIM
Withanolides									Site 1	Site 2	Site 3	
24,25−Epoxywithanolide D	−95.7	−101.5	−63.4	−103.0	−97.7	−118.1	−110.4	−125.7	−119.7	−106.2	−113.6	−95.4
14−Hydroxyixocarpanolide	−88.0	−92.8	−30.7	−52.2	−80.7	−102.3	−77.5	−95.6	−103.6	−92.2	−100.3	−74.3
Physagulin A	−97.4	−103.3	−82.8	−91.9	−91.4	−116.2	−100.5	−119.2	−111.6	−104.4	−106.5	−85.0
Physagulin B	−105.4	−104.2	−85.9	−89.9	−94.2	−107.6	−96.3	−114.4	−110.3	−109.3	−103.8	−82.9
Physagulin C	−98.7	−86.0	−80.6	−104.7	−91.6	−108.7	−98.2	−124.7	−103.4	−100.7	−103.8	−85.9
Physagulin F	−94.8	−88.7	−84.6	−93.6	−88.7	−106.0	−87.9	−107.8	−105.8	−106.3	−91.6	−82.3
Physagulin H	−96.5	−85.2	−83.6	−98.8	−86.0	−101.4	−97.2	−113.0	−106.2	−104.6	−101.7	−79.3
Physagulin I	−95.9	−87.5	−77.2	−94.7	−90.2	−99.9	−91.5	−102.3	−101.2	−103.5	−95.0	−77.7
Physagulin J	−94.4	−84.6	−60.7	−92.5	−88.5	−108.7	−91.2	−100.6	−98.5	−111.2	−100.6	−86.6
Physagulin K	−85.1	−95.8	no dock	−92.5	−94.2	−109.1	−90.1	−92.6	−112.2	−106.4	−97.1	−76.0
Physagulin L	−90.6	−83.3	−67.6	−97.6	−96.2	−112.3	−98.2	−104.5	−117.3	−103.6	−92.0	−68.1
Physagulin L*'*	−108.8	−104.3	−102.2	−94.7	−94.2	−110.7	−87.0	−109.4	−111.5	−98.2	−105.4	−101.2
Physagulin M	−91.2	−91.3	−70.6	−81.4	−96.1	−101.8	−78.6	−87.4	−105.6	−111.5	−101.9	−75.9
Physagulin M*'*	−84.3	−94.8	−30.7	−90.9	−89.8	−106.2	−88.9	−88.5	−110.5	−103.7	−96.3	−106.7
Physagulin N	−94.9	−99.8	−89.5	−95.8	−95.0	−106.2	−80.7	−115.1	−105.1	−112.2	−95.4	−82.5
Physagulin N*'*	−93.2	−83.6	−65.4	−85.2	−86.2	−112.3	−83.4	−103.2	−107.5	−103.8	−85.1	−55.3
Physagulin O	−90.3	−88.8	−88.3	−93.9	−93.4	−103.5	−84.5	−94.6	−99.4	−102.4	−106.3	−72.0
Physalin A	−85.6	−71.4	−41.9	−82.3	−83.6	−93.8	−82.0	−102.9	−88.1	−100.4	−91.4	−81.7
Physalin B	−84.9	−71.7	−48.5	−81.8	−78.4	−88.5	−84.8	−92.6	−92.0	−89.4	−84.8	−73.9
Physalin D	−82.6	−79.1	−54.4	−80.5	−79.6	−91.8	−84.5	−85.0	−89.8	−95.0	−79.9	−71.7
Physalin E	−83.4	−63.3	−30.0	−75.9	−81.3	−83.5	−74.5	−83.7	−96.2	−93.5	−81.5	−76.0
Physalin F	−83.7	−73.8	−57.4	−83.2	−80.7	−92.8	−92.4	−85.1	−90.9	−91.0	−76.6	−74.9
Physalin G	−93.6	−71.0	−81.2	−92.5	−76.9	−78.4	−87.9	−97.9	−100.6	−90.4	−93.0	−69.2
Physalin H	−85.2	−60.7	no dock	−73.0	−81.0	−92.5	−86.0	−81.7	−92.6	−89.6	−81.2	−70.1
Physalin I	−84.2	−51.6	no dock	−68.3	−82.5	−96.2	−87.4	−79.0	−84.0	−86.5	−82.9	−61.6
Physalin J	−83.1	−80.1	−55.5	−79.5	−82.2	−92.4	−78.2	−97.8	−97.0	−94.9	−88.0	−72.1
Physalin K	−82.6	−74.7	−65.5	−84.2	−70.4	−90.2	−77.6	−76.5	−96.7	−87.4	−94.2	−60.7
Physalin U	−83.0	−65.1	−68.5	−94.0	−85.9	−97.6	−93.2	−85.0	−97.0	−93.2	−85.9	−81.6
Physalin V	−83.2	−83.6	−27.1	−84.3	−81.2	−93.9	−93.1	−82.8	−95.0	−92.7	−90.5	−52.8
Physalin W	−93.9	−67.5	−77.2	−89.7	−84.0	−101.5	−92.1	−91.4	−98.1	−99.0	−86.9	−80.2
Physangulide	−103.2	−99.4	−71.3	−106.0	−89.9	−105.2	−83.7	−111.5	−114.6	−108.0	−103.3	−82.8
Physanolide A	−93.4	−96.3	−22.9	−103.0	−84.0	−99.6	−81.4	−106.3	−97.4	−96.3	−86.2	−44.5
Vamonolide	−83.4	−89.7	−52.6	−90.2	−75.4	−96.2	−81.5	−107.1	−101.6	−100.9	−90.8	−77.4
Withangulatin A	−106.1	−102.6	−39.9	−98.6	−96.7	−119.8	−105.4	−122.0	−109.2	−102.9	−107.5	−87.8
Withangulatin B	−95.7	−90.0	no dock	−94.6	−87.5	−99.4	−85.5	−94.2	−99.6	−106.1	−112.4	−92.4
Withangulatin C	−91.7	−87.1	−27.3	−90.6	−88.3	−110.5	−87.0	−99.0	−106.2	−101.2	−113.8	−78.8
Withangulatin D	−101.2	−92.4	no dock	−93.6	−84.6	−105.1	−86.5	−96.2	−92.3	−104.8	−108.2	−86.5
Withangulatin E	−81.0	−77.9	−25.9	−95.5	−76.9	−105.7	−85.8	−99.9	−102.7	−101.4	−100.8	−87.8
Withangulatin F	−54.3	−94.3	−58.6	−83.3	−88.7	−108.4	−88.3	−100.2	−106.2	−97.7	−104.7	−99.5
Withangulatin G	−91.5	−87.6	no dock	−96.1	−74.2	−100.9	−85.5	−100.5	−99.8	−103.2	−95.7	−64.3
Withangulatin H	−95.2	−85.5	−65.1	−85.9	−80.3	−102.5	−75.5	−98.8	−95.8	−106.9	−97.6	−80.7
Withangulatin I	−93.5	−95.3	−82.6	−93.6	−95.5	−113.7	−89.3	−114.2	−116.1	−105.4	−94.5	−71.1

**Table 34:** MolDock docking energies (kJ/mol) of withanolides with *Leishmania infantum* protein targets.

Withanolides	LinfCYP51	LinfGLO2	LinfPnC1	LinfTDR1	LinfTR
24,25−Epoxywithanolide D	−128.1	−102.8	no dock	−103.8	−98.4
14−Hydroxyixocarpanolide	−111.7	−81.1	no dock	−85.3	−94.4
Physagulin A	−106.7	−88.5	no dock	−103.6	−102.7
Physagulin B	−107.7	−82.8	no dock	−99.0	−95.1
Physagulin C	−122.5	−98.8	no dock	−99.7	−94.2
Physagulin F	−108.2	−70.6	no dock	−80.8	−96.1
Physagulin H	−114.9	−92.6	no dock	−96.8	−91.9
Physagulin I	−102.9	−75.1	no dock	−84.5	−97.7
Physagulin J	−115.4	−79.4	no dock	−87.5	−93.2
Physagulin K	−105.4	−76.4	no dock	−88.5	−95.0
Physagulin L	−109.4	−83.5	no dock	−90.1	−94.7
Physagulin L *'*	−107.4	−90.3	no dock	−102.4	−101.4
Physagulin M	−106.5	−78.5	no dock	−95.7	−94.4
Physagulin M *'*	−113.3	−81.6	no dock	−94.8	−91.5
Physagulin N	−114.0	−91.4	no dock	−92.6	−95.2
Physagulin N *'*	−100.5	−87.7	no dock	−85.7	−106.2
Physagulin O	−108.2	−77.9	no dock	−94.4	−92.2
Physalin A	−105.0	−75.2	no dock	−81.4	−73.9
Physalin B	−110.4	−72.6	no dock	−86.0	−93.8
Physalin D	−113.5	−86.0	no dock	−69.0	−93.0
Physalin E	−115.4	−72.5	no dock	−82.9	−92.6
Physalin F	−111.9	−73.6	no dock	−76.2	−93.0
Physalin G	−99.1	−69.1	no dock	−77.5	−91.0
Physalin H	−112.4	−83.7	no dock	−70.2	−94.5
Physalin I	−101.3	−72.0	no dock	−72.8	−94.2
Physalin J	−111.1	−83.9	−34.5	−91.4	−93.0
Physalin K	−93.9	−57.4	no dock	−70.4	−89.5
Physalin U	−99.8	−80.0	−37.3	−78.6	−94.1
Physalin V	−114.5	−67.3	−50.4	−76.0	−102.3
Physalin W	−98.6	−76.3	no dock	−71.1	−81.1
Physangulide	−116.5	−94.1	no dock	−103.3	−103.2
Physanolide A	−104.8	−90.5	no dock	−88.1	−95.2
Vamonolide	−105.8	−78.1	no dock	−88.6	−97.3
Withangulatin A	−114.5	−92.1	no dock	−98.6	−94.2
Withangulatin B	−112.3	−83.6	no dock	−93.2	−90.6
Withangulatin C	−112.1	−77.3	no dock	−89.5	−87.7
Withangulatin D	−106.6	−75.7	no dock	−79.5	−96.3
Withangulatin E	−120.7	−82.1	no dock	−89.9	−85.6
Withangulatin F	−111.9	−80.4	no dock	−93.8	−92.6
Withangulatin G	−115.5	−75.1	no dock	−77.0	−97.7
Withangulatin H	−119.1	−74.0	−40.1	−103.9	−89.9
Withangulatin I	−118.5	−92.6	no dock	−98.5	−97.6

**Table 35:** MolDock docking energies (kJ/mol) of triterpenoids with *Leishmania major* protein targets.

Triterpenoids	LmajCatB	LmajDHODH	LmajdUTPase	LmajNDKb	LmajNH	LmajNMT	LmajOPB	LmajPDE1	LmajPTR1	LmajMetRS	LmajTyrRS	LmajUGPase
α−Amyrin	−74.4	−81.7	−52.9	−55.1	−73.4	−84.8	−75.6	−100.9	−72.3	−71.8	−84.5	−82.7
β−Amyrin	−43.0	−78.8	−66.1	−59.1	−76.8	−87.7	−84.4	−83.8	−65.7	−72.6	−84.0	−82.4
Betulin	−71.9	−85.3	−75.8	−61.5	−99.0	−93.6	−94.4	−83.0	−71.2	−80.8	−99.1	−85.5
Betulinaldehyde	−74.6	−81.7	−75.7	−71.9	−98.9	−93.5	−94.2	−88.4	−76.0	−78.5	−99.1	−89.4
Betulinic acid	−74.2	−88.5	−66.7	−61.3	−96.3	−104.6	−89.1	−82.6	−73.5	−82.0	−103.3	−88.7
Corosolic acid	−80.2	−74.7	−81.7	−63.1	−91.3	−107.7	−84.5	−106.4	−81.6	−79.3	−86.4	−81.4
Erythrodiol	−34.6	−84.6	−64.6	−64.9	−79.5	−92.7	−87.0	−84.9	−67.2	−74.7	−87.6	−83.5
Friedelin	−57.3	−72.5	−74.6	−53.9	−84.9	−75.9	−78.0	−79.6	−75.9	−71.3	−102.4	−76.5
Isoiguesterin	−67.3	−79.4	−64.7	−73.5	−80.5	−95.5	−81.0	−92.2	−77.3	−77.2	−88.2	−85.2
20− *epi*−Isoiguesterinol	−69.7	−74.0	−64.7	−83.9	−85.3	−96.4	−98.2	−96.2	−84.4	−81.4	−83.6	−78.6
Lawnermis acid methyl ester	−75.8	−95.9	−81.6	−70.3	−76.5	−91.4	−81.6	−98.3	−66.7	−78.8	−92.2	−88.3
Lupeol	−73.2	−84.6	−76.7	−53.1	−99.0	−87.5	−91.5	−85.5	−69.9	−74.3	−96.2	−75.9
Methyl *seco*−3,4−betulonic acid	−87.2	−93.8	−84.8	−76.1	−101.2	−113.5	−96.5	−106.3	−97.0	−79.2	−90.1	−110.1
3− *O*−Methyl−6−oxopristimerol	−91.4	−92.8	−75.9	−77.1	−99.5	−102.5	−88.4	−95.2	−79.1	−94.0	−99.9	−96.1
Oleanolic acid	−82.5	−90.6	−80.2	−66.3	−72.7	−97.7	−79.8	−95.3	−66.6	−69.7	−88.4	−76.2
*epi*−Oleanolic acid	−75.9	−76.7	−77.2	−54.2	−72.4	−83.7	−88.7	−98.5	−70.3	−84.2	−82.3	−95.6
6−Oxopristimerol	−87.2	−91.5	−72.9	−76.4	−98.8	−103.6	−90.2	−91.8	−82.5	−93.2	−93.8	−101.2
(24 *Z*)−3−Oxotirucalla−7,24−dien−26−oic acid	−96.4	−95.4	−86.1	−129.2	−104.4	−95.7	−99.7	−106.1	−90.3	−91.9	−102.2	−98.9
Pristimerin	−69.3	−87.8	−72.3	−77.6	−88.1	−112.9	−86.3	−86.4	−87.8	−86.6	−102.0	−88.6
Rotundic acid	−78.5	−85.1	−74.2	−49.1	−55.9	−85.7	−86.8	−98.4	−65.6	−80.8	−78.5	−83.4
Taraxerol	−22.5	−83.4	−60.2	−48.0	−70.1	−82.4	−88.0	−78.8	−85.5	−85.1	−84.9	−88.1
Ursolic acid	−70.8	−80.0	−74.0	−59.1	−72.0	−92.4	−80.4	−73.7	−71.9	−82.7	−84.3	−89.9
Uvaol	−74.9	−82.4	−67.9	−67.8	−75.2	−88.3	−85.4	−89.9	−75.2	−74.9	−86.4	−87.0
Wallichianol	−81.9	−93.2	−71.1	−74.6	−87.2	−102.7	−92.0	−75.2	−86.2	−80.1	−80.6	−89.4

**Table 36:** MolDock docking energies (kJ/mol) of triterpenoids with *Leishmania donovani* and *L. mexicana* protein targets.

	LdonCatB	LdonCyp	LdonDHODH	LdonNMT	LmexGAPDH	LmexGPDH	LmexPGI	LmexPMM	LmexPYK	LmexPYK	LmexPYK	LmexTIM
Triterpenoids									Site 1	Site 2	Site 3	
α−Amyrin	−79.7	−79.4	−19.0	−53.4	−73.7	−88.0	−73.5	−71.7	−70.4	−87.4	−85.4	−54.4
β−Amyrin	−65.3	−75.6	no dock	−64.7	−73.9	−77.2	−65.3	−71.0	−79.9	−94.4	−75.2	−35.5
Betulin	−80.1	−78.2	no dock	−77.8	−81.6	−101.1	−79.1	−82.6	−87.1	−98.0	−83.7	−70.3
Betulinaldehyde	−82.8	−78.6	no dock	−67.2	−75.9	−96.9	−83.5	−84.5	−87.5	−96.6	−82.5	−75.0
Betulinic acid	−86.3	−65.2	no dock	−80.7	−77.7	−97.3	−81.0	−87.8	−89.5	−97.8	−85.4	−76.3
Corosolic acid	−80.8	−80.2	no dock	−78.2	−78.0	−93.2	−81.9	−75.8	−80.7	−89.6	−73.0	−60.9
Erythrodiol	−57.7	−75.8	−30.0	−69.5	−76.1	−80.8	−69.9	−73.0	−79.2	−96.6	−75.4	−39.3
Friedelin	−65.9	−53.9	−48.8	−63.2	−72.7	−83.8	−64.8	−77.1	−73.6	−86.1	−76.7	−70.1
Isoiguesterin	−69.9	−70.0	−48.8	−70.4	−76.6	−80.9	−68.8	−83.0	−80.7	−87.9	−77.0	−65.9
20− *epi*−Isoiguesterinol	−70.5	−83.0	−55.8	−74.8	−75.9	−93.0	−73.1	−91.0	−88.9	−88.7	−76.3	−58.3
Lawnermis acid methyl ester	−73.7	−77.4	−44.5	−73.4	−84.7	−97.0	−80.1	−77.6	−81.5	−96.3	−98.1	−57.7
Lupeol	−80.7	−76.5	no dock	−71.9	−88.5	−93.0	−72.1	−81.3	−82.0	−96.0	−79.6	−69.2
Methyl *seco*−3,4−betulonic acid	−91.6	−83.9	no dock	−92.0	−87.8	−102.4	−89.7	−98.0	−109.4	−102.6	−86.7	−82.2
3− *O*−Methyl−6−oxopristimerol	−90.8	−83.7	no dock	−85.0	−76.0	−90.2	−78.6	−92.9	−109.5	−96.4	−83.2	−61.4
Oleanolic acid	−82.4	−74.2	no dock	−69.7	−79.0	−86.9	−69.9	−80.8	−77.5	−90.4	−79.9	−74.9
*epi*−Oleanolic acid	−61.1	−73.7	−50.7	−69.1	−81.2	−88.8	−66.3	−78.6	−79.6	−91.9	−74.8	−65.7
6−Oxopristimerol	−90.6	−84.3	−60.9	−82.9	−80.8	−90.0	−79.1	−85.5	−98.4	−95.2	−94.1	−68.0
(24 *Z*)−3−Oxotirucalla−7,24−dien−26−oic acid	−100.0	−88.1	−82.2	−89.0	−94.3	−108.5	−99.2	−104.0	−122.1	−104.4	−100.1	−91.2
Pristimerin	−71.3	−78.4	−46.7	−85.9	−93.1	−90.7	−74.4	−102.4	−96.4	−97.4	−80.4	−63.0
Rotundic acid	−71.6	−78.2	−36.3	−77.0	−75.8	−100.3	−71.4	−75.5	−80.8	−93.7	−82.5	−58.0
Taraxerol	−51.7	−78.0	−29.6	−61.4	−69.4	−82.8	−68.2	−90.4	−81.4	−90.1	−69.8	−81.4
Ursolic acid	−69.6	−78.7	no dock	−71.7	−76.5	−97.3	−80.3	−75.5	−71.8	−93.0	−75.4	−46.5
Uvaol	−81.4	−79.1	no dock	−51.7	−75.9	−95.0	−78.0	−74.3	−76.4	−92.0	−82.8	−64.9
Wallichianol	−81.5	−70.6	−58.4	−85.4	−83.8	−87.6	−80.4	−91.9	−96.8	−93.8	−82.3	−77.4

**Table 37:** MolDock docking energies (kJ/mol) of triterpenoids with *Leishmania infantum* protein targets.

Triterpenoids	LinfCYP51	LinfGLO2	LinfPnC1	LinfTDR1	LinfTR
α−Amyrin	−99.3	−66.0	no dock	−66.7	−71.9
β−Amyrin	−98.9	−72.6	−43.9	−73.5	−68.4
Betulin	−101.6	−73.8	−30.4	−79.1	−79.0
Betulinaldehyde	−104.8	−74.8	no dock	−74.1	−85.8
Betulinic acid	−95.5	−75.2	no dock	−80.5	−85.8
Corosolic acid	−111.8	−64.5	no dock	−74.2	−82.2
Erythrodiol	−100.4	−71.2	no dock	−76.7	−78.9
Friedelin	−92.7	−64.4	−40.3	−71.0	−75.4
Isoiguesterin	−83.7	−70.6	no dock	−81.9	−77.2
20− *epi*−Isoiguesterinol	−81.1	−71.1	no dock	−80.4	−77.1
Lawnermis acid methyl ester	−97.9	−72.9	−33.4	−72.3	−81.2
Lupeol	−97.9	−70.7	−35.1	−77.1	−81.0
Methyl *seco*−3,4−betulonic acid	−94.7	−67.1	no dock	−88.7	−91.9
3− *O*−Methyl−6−oxopristimerol	−98.6	−73.3	−12.0	−84.9	−92.2
Oleanolic acid	−105.6	−70.7	no dock	−79.2	−74.9
*epi*−Oleanolic acid	−105.5	−74.4	no dock	−81.6	−80.5
6−Oxopristimerol	−95.8	−66.4	no dock	−77.6	−94.9
(24 *Z*)−3−Oxotirucalla−7,24−dien−26−oic acid	−112.4	−99.4	no dock	−83.9	−97.0
Pristimerin	−97.5	−78.3	no dock	−82.4	−84.9
Rotundic acid	−107.8	−60.5	−33.1	−76.5	−78.6
Taraxerol	−91.5	−74.2	−39.1	−74.8	−76.9
Ursolic acid	−102.3	−62.5	no dock	−69.4	−76.3
Uvaol	−101.1	−65.3	no dock	−69.4	−77.4

**Table 38:** MolDock docking energies (kJ/mol) of quassinoids and steroids with *Leishmania major* protein targets.

	LmajCatB	LmajDHODH	LmajdUTPase	LmajNDKb	LmajNH	LmajNMT	LmajOPB	LmajPDE1	LmajPTR1	LmajMetRS	LmajTyrRS	LmajUGPase
Quassinoids												
15−β−Heptylchaparrinone	−92.7	−121.0	−89.4	−118.4	−101.9	−112.4	−98.2	−109.3	−96.2	−120.5	−106.5	−101.0
Simalikalactone D	−88.4	−106.2	−80.5	−99.3	−84.9	−98.5	−63.6	−105.2	−91.8	−111.7	−105.5	−111.1
**Steroids**												
Cholesterol	−90.5	−95.2	−93.3	−106.4	−101.7	−98.6	−111.4	−109.2	−109.5	−115.2	−99.3	−97.7
Clerosterol	−97.9	−100.6	−95.5	−110.6	−104.8	−102.4	−91.6	−113.3	−110.8	−121.3	−101.0	−101.9
24−Hydroperoxy−24−vinylcholesterol	−81.0	−99.9	−92.1	−111.0	−103.4	−105.0	−115.9	−117.7	−109.8	−127.0	−100.2	−112.4
Lanosterol	−85.0	−85.4	−81.3	−94.1	−103.2	−96.3	−111.4	−108.4	−87.2	−88.8	−97.2	−99.3
Saringosterol	−88.4	−103.8	−89.8	−108.1	−105.9	−101.6	−109.5	−115.5	−107.1	−126.4	−99.0	−106.1
β−Sitosterol	−94.8	−98.7	−94.8	−106.4	−105.6	−102.1	−101.7	−111.9	−110.0	−121.5	−102.2	−111.6
Stigmasterol	−87.8	−102.3	−94.6	−105.6	−108.1	−101.1	−101.0	−109.8	−109.3	−121.4	−106.4	−102.1

**Table 39:** MolDock docking energies (kJ/mol) of quassinoids and steroids with *Leishmania donovani* and *L. mexicana* protein targets.

	LdonCatB	LdonCyp	LdonDHODH	LdonNMT	LmexGAPDH	LmexGPDH	LmexPGI	LmexPMM	LmexPYK	LmexPYK	LmexPYK	LmexTIM
Quassinoids									Site 1	Site 2	Site 3	
15−β−Heptylchaparrinone	−93.0	−90.0	−88.8	−100.5	−84.9	−111.8	−90.5	−110.0	−123.2	−108.0	−98.8	−92.0
Simalikalactone D	−98.1	−102.9	no dock	−90.9	−89.5	−115.2	−70.9	−103.9	−93.4	−99.6	−107.4	−75.8
**Steroids**												
Cholesterol	−95.4	−99.4	−78.2	−90.9	−91.6	−102.8	−93.8	−104.3	−102.0	−95.5	−100.2	−87.3
Clerosterol	−82.5	−99.9	−89.1	−99.9	−92.8	−104.0	−80.0	−108.3	−106.8	−104.2	−100.1	−96.0
24−Hydroperoxy−24−vinylcholesterol	−97.8	−98.0	−101.7	−89.9	−93.5	−97.2	−77.0	−111.9	−103.8	−104.4	−89.5	−91.6
Lanosterol	−84.1	−88.4	−55.0	−81.9	−81.1	−99.7	−91.5	−98.8	−103.6	−93.5	−103.8	−82.4
Saringosterol	−97.9	−106.5	−98.9	−99.1	−86.7	−113.8	−91.1	−111.0	−106.5	−102.3	−88.7	−95.8
β−Sitosterol	−100.9	−104.5	−86.7	−88.8	−92.5	−100.0	−96.7	−107.8	−105.5	−97.3	−92.6	−95.0
Stigmasterol	−97.0	−98.5	−93.7	−92.3	−90.2	−112.3	−88.5	−107.6	−106.0	−104.3	−97.8	−96.5

**Table 40:** MolDock docking energies (kJ/mol) of quassinoids and steroids with *Leishmania infantum* protein targets.

Quassinoids	LinfCYP51	LinfGLO2	LinfPnC1	LinfTDR1	LinfTR
15−β−Heptylchaparrinone	−107.6	−100.4	no dock	−92.2	−96.8
Simalikalactone D	−98.5	−85.8	no dock	−83.7	−80.2
**Steroids**					
Cholesterol	−110.9	−86.9	no dock	−87.4	−93.0
Clerosterol	−116.3	−94.5	no dock	−91.4	−95.8
24−Hydroperoxy−24−vinylcholesterol	−121.5	−89.9	no dock	−91.4	−96.6
Lanosterol	−116.0	−85.7	no dock	−81.5	−90.4
Saringosterol	−117.2	−90.0	no dock	−97.9	−99.8
β−Sitosterol	−113.8	−87.6	no dock	−94.1	−103.0
Stigmasterol	−116.1	−86.4	no dock	−96.6	−98.4

**Figure 28:**
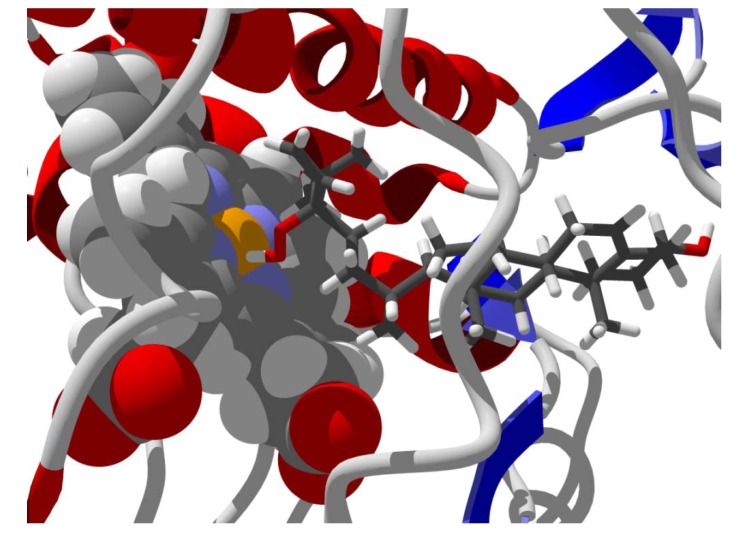
Lowest-energy poses of 24-hydroperoxy-24-vinylcholesterol with *L. infantum* sterol 14α-demethylase (LinfCYP51, PDB 3l4d). Note the proximity of the hydroperoxy group of the ligand with the Fe atom of the heme cofactor.

Not surprisingly, all of the steroids and many of the triterpenoids examined in this study showed significant docking preference for *L. infantum* sterol 14α-demethylase (LinfCYP51). This had been noted previously with *Trypanosoma brucei* sterol 14α-demethylase [71]. In particular, 24-hydroperoxy-24-vinylcholesterol (docking energy = −121.5 kJ/mol) and 24,25-epoxywithanolide D (docking energy = −128.1 kJ/mol) were strongly docking with LinfCYP51. The lowest-energy pose of 24-hydroperoxy-24,25-vinylcholesterol with LinfCYP51 places the hydroperoxy group of the ligand adjacent to the heme Fe (Figure 28); this ligand, then, can presumably oxidize the Fe and render the enzyme inactive.

**Figure 29:**
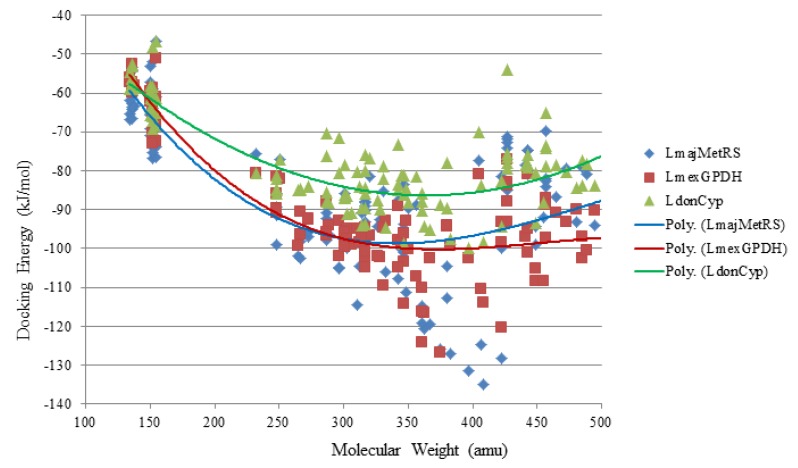
Plots of docking energies *vs.* molecular weights for representative isoprenoids with three *Leishmania* protein targets.

An examination of docking energies with respect to ligand molecular size suggests that for terpenoid ligands there is a threshold where larger size does not correspond to stronger binding to the protein target. A plot of molecular weights of representative terpenoids (monoterpenoids, germacranolide sesquiterpenoids, labdane diterpenoids, and triterpenoids) and docking energies to three different protein targets (LmajMetRS, LmexGPDH, and LdonCyp) (Figure 29) shows that strongest docking energies are terpenoids with molecular weights around 360–430 amu.

## 3. Computational Methods

Protein-ligand docking studies were carried out based on the crystal structures of verified *Leishmania* protein drug targets: *L. major* cathepsin B, LmajCatB (prepared by structural homology to *Trypanosoma brucei* cathepsin B, PDB 3hhi [72]), *L. major* dihydroorotate dehydrogenase, LmajDHODH (PDB 3gye [73], PDB 3mhu, and PDB 3mjy [74]), *L. major* methionyl-tRNA synthetase, LmajMetRS (PDB 3kfl [51]), *L. major* nucleoside diphosphate kinase b, LmajNDKb (PDB 3ngs, PDB 3ngt, and PDB 3ngu [38]), *L. major* nucleoside hydrolase, LmajNH (PDB 1ezr [35]), *L. major N*-myristoyltransferase, LmajNMT (PDB 2wsa, PDB 3h5z [47], and PDB 4a30 [75]), *L. major* oligopeptidase B, LmajOPB (PDB 2xe4 [27]), *L. major* phosphodiesterase 1, LmajPDE1 (PDB 2r8q [40]), *L. major* pteridine reductase 1, LmajPTR1 (PDB 1e7w [43], PDB 1w0c [76], PDB 2bf7 [77], and PDB 3h4v [78]), *L. major* tyrosyl-tRNA synthetase, LmajTyrRS (PDB 3p0h and PDB 3p0j [52]), *L. major* uridine diphosphate-glucose pyrophosphorylase, LmajUGPase (PDB 2oef and PDB 2oeg [10]), *L. major* deoxyuridine triphosphate nucleotidohydrolase, LmajdUTPase (PDB 2yay and PDB 2yb0 [33]), *L. donovani* cathepsin B, LdonCatB (prepared by structural homology to *T. brucei* cathepsin B, PDB 3hhi [72]), *L. donovani* cyclophilin, LdonCyp (PDB 2haq [49] and PDB 3eov [79]), *L. donovani* dihydroorotate dehydrogenase, LdonDHODH (PDB 3c61 [80]), *L. donovani N*-myristoyltransferase, LdonNMT (PDB 2wuu [48]), *L. mexicana* glyceraldehyde-3-phosphate dehydrogenase. LmexGAPDH (PDB 1a7k [11] and PDB 1gyp [81]), *L. mexicana* glycerol-3-phosphate dehydrogenase, LmexGPDH (PDB 1evz [14], PDB 1m66, PDB 1n1e and PDB 1n1g [82]), *L. mexicana* phosphoglucose isomerase, LmexPGI (PDB 1q50 and PDB 1t10 [8]), *L. mexicana* phosphomannomutase, LmexPMM (PDB 2i54 and PDB 2i55 [83]), *L. mexicana* pyruvate kinase, LmexPYK (PDB 1pkl [6], PDB 3hqp [84], and PDB 3pp7 [85]), *L. mexicana* triosephosphate isomerase, LmexTIM (PDB 2vxn [17] and PDB 2y61 [86]), *L. infantum* sterol 14α-demethylase, LinfCYP51 (PDB 3l4d [87]), *L. infantum* glyoxalase II, LinfGLO2 (PDB 2p1e and PDB 2p18 [21]), *L. infantum* nicotinamidase, LinfPnC1 (PDB 3r2j [34]), *L. infantum* thiol-dependent reductase I, LinfTDR1 (PDB 4ags [19]), and *L. infantum* trypanothione reductase, LinfTR (PDB 2yau [88] and PDB 4adw [89] and PDB 4apn [90]) Prior to docking all solvent molecules and the co-crystallized ligands were removed from the structures. Molecular docking calculations for all compounds with each of the proteins were undertaken using Molegro Virtual Docker v. 5.0 [91,92], with a sphere large enough to accommodate the cavity centered on the binding sites of each protein structure in order to allow each ligand to search. If a co-crystallized inhibitor or substrate was present in the structure, then that site was chosen as the binding site. If no co-crystallized ligand was present, then suitably sized cavities were used as potential binding sites. Standard protonation states of the proteins based on neutral pH were used in the docking studies. The protein was used as a rigid model structure; no relaxation of the protein was performed. Assignments of charges on each protein were based on standard templates as part of the Molegro Virtual Docker program; no other charges were necessary to be set. Each ligand structure was built using Spartan ’10 for Windows [93]. The structures were geometry optimized using the MMFF force field [94]. Flexible ligand models were used in the docking and subsequent optimization scheme. As a test of docking accuracy and for docking energy comparison, co-crystallized ligands were re-docked into the protein structures (See Table 41). Different orientations of the ligands were searched and ranked based on their energy scores. The RMSD threshold for multiple cluster poses was set at <1.00 Å. The docking algorithm was set at maximum iterations of 1500 with a simplex evolution population size of 50 and a minimum of 30 runs for each ligand. Each binding site of oligomeric structures was searched with each ligand. The lowest-energy (strongest-docking) poses for each ligand in each protein target are summarized in Table 1, Table 2, Table 3, Table 4, Table 5, Table 6, Table 7, Table 8, Table 9, Table 10, Table 11, Table 12, Table 13, Table 14, Table 15, Table 16, Table 17, Table 18, Table 19, Table 20, Table 21, Table 22, Table 23, Table 24, Table 25, Table 26, Table 27 and Table 28.

The primary sequence of the cathepsin B-like cysteine protease from *T. brucei* (TbCatB, PDB 3 hhi [72]) was compared to the query sequences of the same functional enzyme from *L. donovani* (LdonCatB) and *L. major* (LmajCatB) using the Protein BLAST (Basic Local Alignment Search Tool). Regions of local similarity and identity were found between the query sequences of LdonCatB and LmajCatB when compared to the model sequence of TbCatB. Both LdonCatB and LmajCatB sequences had 52% and 54% identity with that of TbCatB, respectively. The three-dimensional structure of TbCatB has been determined to 1.6 Å (PDB 3hhi [72]) but there is no structural information available for either LdonCatB or LmajCatB. Calculated models for both LdonCatB and LmajCatB were obtained from combining sequence information of the unknown target structures for LdonCatB and LmajCatB with the known model of TbCatB. The alignment between target and model sequences was used to modify the model PDB TbCatB file by pruning non-conserved residues to the last common atoms using the CCP4 chainsaw molecular replacement utility [95,96] leaving conserved residues unchanged. The resulting models were refined with conjugate gradient minimization with no experimental energy terms used in the crystallographic and NMR System (CNS) program suite [97]. The resulting detailed model was refined with conjugate gradient minimization with no experimental energy terms used. All atoms of the molecules were unrestrained and were minimized for 500 steps with a continuous dielectric constant of one.

**Table 41:** MolDock docking energies of co-crystallized ligands and root-mean-squared deviations between the co-crystallized ligand and the re-docked poses of the co-crystallized ligand with *Leishmania* protein crystal structures.

Protein Target	PDB code	Co-crystallized ligand	*E* (kJ/mol)	RMSD (Å)
LmajCatB	homology	none	---	
LmajDHODH	3gye	none	---	
	3mhu	5-nitroorotic acid	−102.2	0.37
	3mjy	5-aminoorotic acid	−91.4	0.47
LmajdUTPase	2yay	2 *'*-deoxyuridine-5*'*-α,β-imido-triphosphate	−117.1	1.36
	2yb0	2 *'*-deoxyuridine	−80.3	0.93
LmajNDKb	3ngs	none	---	
	3ngt	adenosine 5 *'*-monophosphate	−122.5	4.07
	3ngu	adenosine 5 *'*-diphosphate	−143.6	4.29
LmajNH	1ezr	none	---	
LmajNMT	2wsa	2,6-dichloro-4-(2-piperazin-1-ylpyridin-4- yl)- *N*-(1,3,5-trimethyl-1*H*-pyrazol-4-yl)benzenesulfonamide	−121.0	0.88
	3h5z	myristoyl-CoA	−115.4	7.52
	4a30	4-bromo-2,6-dichloro- *N*-(1,3,5-trimethyl-1H-pyrazol-4-yl)benzene-sulfonamide	−84.5	1.80
LmajOPB	2xe4	none	---	
LmajPDE1	2r8q	3-isobutyl-1-methylxanthine	−78.1	3.41
LmajPTR1	1e7w	methotrexate	−147.1	5.63
	1w0c	2,4,6-triaminoquinazoline	−72.7	0.52
	2bf7	7,8-dihydrobiopterin	−93.7	0.61
	3h4v	methyl 1-(4-{[(2,4-diaminopteridin-6-yl)methyl]amino}benzoyl)piperidine-4-carboxylate	−129.9	5.50
LmajMetRS	3kfl	methionyl-adenylate	−172.5	3.35
LmajTyrRS	3p0h	3,7,3 *'*,4*'*-tetrahydroxyflavone	−90.3	0.56
	3p0j	tyrosinol	−77.2	1.42
LmajUGPase	2oef	none	---	
	2oeg	uridine-5 *'*-phosphate-glucose	−143.9	3.69
LdonCatB	homology	none	---	
LdonCyp	2haq	none	---	
	3eov	omitted	---	
LdonDHODH	3c61	orotic acid	−64.2	9.23
LdonNMT	2wuu	none	---	
LmexGAPDH	1a7k	none	---	
	1gyp	none	---	
LmexGPDH	1evz	NAD^+^	−161.6	3.80
	1m66	2-bromo-6-chloropurine	−54.4	5.56
	1n1e	adenosine 5 *'*-(trihydrogendiphosphate) P*'*-5*'*-ester with 3-(aminocarbonyl)-4-(1-hydroxyl-2-oxo-3-phosphonooxypropyl)-1β-d-ribofuranosylpyridinium inner salt	−269.7	3.00
	1n1g	2-bromo-6-chloropurine	−53.8	4.24
LmexPGI	1q50	none	---
	1t10	fructose-6-phosphate	−74.9	2.69
LmexPMM	2i54	citric acid	−73.5	3.66
	2i55	1,6-di- *O*-phosphono-β-d-glucopyranose	−120.5	2.33
LmexPYK				
ATP site	1pkl	none	---	
	3hqp	adenosine-5 *'*-triphosphate	−138.5	7.74
	3pp7	suramin	−123.6	1.07
FDP site	1pkl	none	---
	3hqp	fructose-2,6-diphosphate	−132.8	0.63
LmexTIM	2vxn	phosphoglycolohydroxamic acid	−62.6	0.86
	2y61	glycerol-1-phosphate	−55.9	1.26
LinfCYP51	3l4d	2-(2,4-difluorophenyl)-1,3-di(1 *H*-1,2,4-triazol-1-yl)propan-2-ol	−91.3	1.39
LinfGLO2	2p1e	lactic acid	−57.7	1.88
	2p18	acetic acid	−55.8	1.38
LinfPnC1	3r2j	nicotinic acid	−65.5	1.66
LinfTDR1	4ags	glutathione	−102.7	6.96
LinfTR	2yau	3,4,5-triacetyloxy-6-(acetyloxymethyl)oxane-2-thiol	−90.0	5.54
	4adw	trypanothione	−142.6	10.16
	4apn	4-{[1-(4-ethylphenyl)-2-methyl-5-(4-methylsulfanylphenyl)pyrrol-3-yl]-methyl}thiomorpholine	−105.6	5.51

## 4. Conclusions

Numerous antiparasitic plant-derived natural products have been identified but the molecular target(s) of most of these compounds remain unknown. This gap in knowledge impedes further characterization and optimization of the antiparasitic activity of many of these compounds. In this molecular docking study, we have identified molecular targets in *Leishmania* that preferentially interact with certain classes of antiparasitic isoprenoids from plants. Consequently, *Leishmania* proteins that have structural motifs similar to those identified in this work may be explored as potential drug targets by antileishmanial drug discovery programs. It is important to point out that: (a) there are likely additional *Leishmania* proteins or other biochemical targets that have not yet been identified; (b) some of the antiparasitic terpenoids examined in the study may have poor bioavailability due to limited solubility, membrane permeability, hydrolysis, or other metabolic transformations; (c) the ligands may also target homologous isozymes in humans. Therefore, pharmacokinetic and pharmacodymanic studies as well as structure-based design and optimization studies are needed to resolve issues of bioavailability and selectivity. In summary, this *in-silico* molecular docking study has provided evidence for what classes and structural types of terpenoids may be targeting certain *Leishmania* protein targets and could provide the framework for synthetic modification of antiparasitic terpenoids, *de novo* synthesis of structural designs, and further phytochemical investigations.
